# Tumor-infiltrating macrophage associated lncRNA signature in cutaneous melanoma: implications for diagnosis, prognosis, and immunotherapy

**DOI:** 10.18632/aging.205606

**Published:** 2024-03-13

**Authors:** Qi Wan, Yuhua Deng, Ran Wei, Ke Ma, Jing Tang, Ying-Ping Deng

**Affiliations:** 1 Department of Ophthalmology, West China Hospital of Sichuan University, Chengdu, Sichuan, China; 2 Department of Infection Control, West China Hospital of Sichuan University, Chengdu, Sichuan, China

**Keywords:** macrophage, lncRNA, prognosis, immunotherapy, cutaneous, melanoma

## Abstract

Along with the increasing knowledge of long noncoding RNA, the interaction between the long noncoding RNA (lncRNA) and tumor immune infiltration is increasingly valued. However, there is a lack of understanding of correlation between regulation of specific lncRNAs and tumor-infiltrating macrophages within melanoma. In this research, a macrophage associated lncRNA signature was identified by multiple machine learning algorithms and the robust and effectiveness of signature also validated in other independent datasets. The signature contained six specific lncRNAs (PART1, LINC00968, LINC00954, LINC00944, LINC00518 and C20orf197) was constructed, which could diagnose melanoma and predict the prognosis of patients. Moreover, our signature achieves higher accuracy than the previous well-established markers and regarded as an independent prognostic indicator. The pathway enrichment revealed that these lncRNAs were closely correlated with many immune processes. In addition, the signature was associated with different immune microenvironment and applied to predict response of immune checkpoint inhibitor therapy (low risk of patients well respond to anti-PD-1 therapy and high risk is insensitive to anti-CTLA-4 therapy). Therefore, our finding supplies a more accuracy and effective lncRNA signature for tumor-infiltrating macrophages targeting treatment approaches and affords a new clinical application for predicting the response of immunotherapies in melanomas.

## INTRODUCTION

Malignant melanoma is the most dangerous skin cancer, which originates from melanocytes and possesses a larger proportion of metastases and death [1]. Recent evidence has shown that tumor microenvironment plays a critical role in formation and development of melanoma [2, 3]. Various infiltrating immune cells positively or negatively regulate tumor cell behaviors and finally result in tumor growth and metastasis [4]. Of particular interest, macrophages work as a major component of infiltrating immune cells, which participate to a large degree in melanoma cells’ proliferation, metastasis and resistance to anticancer therapies [5, 6]. They can secrete growth factors, such as vascular endothelial growth factor (VEGF) and epidermal growth factor (EGF), which stimulate tumor angiogenesis and facilitate metastatic spread. Tumor associated macrophages (TAMs) also produce matrix metalloproteinases (MMPs) that degrade extracellular matrix components, allowing melanoma cells to invade surrounding tissues and form distant metastases [7, 8]. Furthermore, TAMs play a critical role in suppressing the anti-tumor immune response. They can inhibit the activation and function of cytotoxic T cells, natural killer cells, and dendritic cells, thereby dampening the immune system’s ability to recognize and eliminate melanoma cells. This immunosuppressive function of TAMs is mediated by the secretion of immunosuppressive cytokines, such as interleukin-10 (IL-10) and transforming growth factor-beta (TGF-β) [9, 10]. Hence, TAMs have been proven as an important therapeutic target to improve the efficacy of immunotherapy.

According to research on mammalian transcriptomes, protein-coding genes account for just 1.5 percent of the human genome. While around 70% of the overall human genome is continually producing a broad array of non-coding RNAs (ncRNAs), which are classified as small ncRNAs (200 nt) and long ncRNAs (lncRNAs, >200 nt) based on transcript length. Increasing evidence suggests that lncRNAs are involved in a wide range of biological activities, including cell proliferation, differentiation, migration, invasion, and death [11–14]. It has been proven that abnormal expression of lncRNAs plays essential jobs in carcinogenesis, such as gastric cancer, prostate cancer, and leukemia [15]. Moreover, previous studies suggested that lncRNAs widely regulated immune reactions such as antigen presentation or release, immune cell activation or infiltration. For instance, Elling et al. demonstrated that lincRNA-COX2 can directly regulate immune response by activating or inhibiting a class of immune genes [16]. Song et al. recently revealed that the NF-κB interacting lncRNA (NKILA) can activate inflammatory responses in the tumor microenvironment. Low NKILA expression has been linked to a poor prognosis of breast cancer [17]. Besides, Krawczyk et al. indicated that P50-associated cyclooxygenase-2 extragenic RNA (PACER) takes a crucial factor in the differentiation of macrophages [18]. Although lncRNAs emerge as a hot research topic in many aspects including cancer immunology, few numbers of macrophage associated lncRNAs have been investigated so far, and a new prognostic macrophage associated lncRNAs signature is needed continue to surprise us.

Therefore, in this research, we identified macrophage-associated lncRNAs from the ImmLnc database and then thoroughly analyzed the differentially expressed macrophage associated lncRNAs between tumor and normal in multiple public datasets. Next, we developed a macrophage lncRNA signature by integrating immune-related lncRNAs and clinical outcomes. We discovered that the expression of macrophage associated lncRNAs signature takes a crucial role in the prognosis of melanoma and displays more effective performance for prediction of immunotherapy response in melanoma patients.

## MATERIALS AND METHODS

### Macrophage associated lncRNA in melanoma

In TIMER2.0 website (http://timer.comp-genomics.org/), we astonished observed that tumor infiltrated macrophage cells were regarded as a prognostic factor and intimately associated with BRAF mutant in cutaneous melanoma patients (Supplementary Figure 1). Thus, the 3346 macrophage associated lncRNAs in cutaneous melanoma (Supplementary Table 1) were retrieved from the ImmLnc database (http://bio-bigdata.hrbmu.edu.cn/ImmLnc), which affords tools to explore the immune associated function of lncRNAs such as the correlation between lncRNA and immune cell types, lncRNA and pathways and cancer related lncRNAs across 33 types of cancer [19].

### Melanomas collection and normal controls

**Figure 1:**
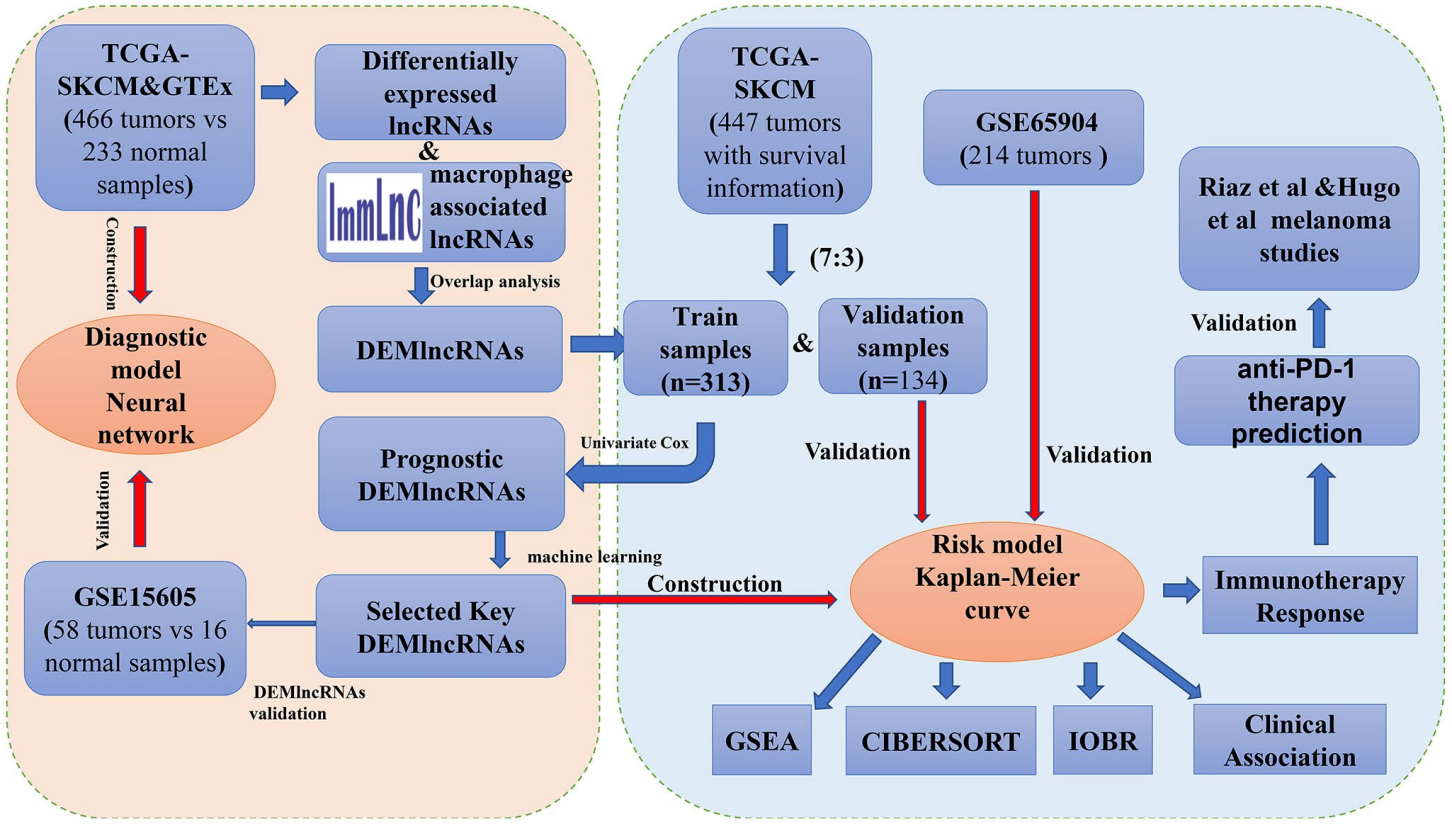
The complete workflow of the analysis in this present study.

The TCGA-SKCM dataset (https://portal.gdc.cancer.gov) was used to obtain the lncRNAs expression and clinical variables of cutaneous melanoma, and the normal healthy skin tissue samples were obtained from the Genotype-Tissue Expression website (GTEx) (https://www.gtexportal.org/) to match the tumor samples. TPMs values were utilized to describe the expression level of lncRNAs for TCGA-SKCM & GTEx. Five independent melanoma patient datasets were acquired from GEO database for outside verification, which including GSE65904, GSE15605, GSE78220 (Hugo et al. study) [20] and GSE91061 (Riaz et al. study) [21]. Furthermore, to predict immunotherapy response, A prior cohort managed with CTLA-4 and PD-1 inhibitors was obtained from a previously published study [22]. The transcriptome profiles (FPKM) of these datasets were processed by applying their corresponding platform and was transformed into TPMs. Firstly, probes were annotated based on the annotation profile supplied by the platform, and non-matched probes were removed. If several probes were mapped to the same gene, the average value would be used to reflect gene expression. Variations in lncRNAs with low expression levels (TPMs <0.01) will then be eliminated due to their low expression level. Finally, the raw data were log2(× + 1) transformed and quantile normalized. Figure 1 depicts a simplified procedure for the current investigation.

### Differential expression analysis of macrophage associated lncRNAs (DEMlncRNAs)

To explore the differentially expressed lncRNAs, samples in TCGA-SKCM & GTEx dataset were divided into melanoma and normal skin groups. The differentially expressed analysis of lncRNAs between melanoma and normal skin was conducted by using ‘limma package’ in R software [23]. The cutoff criterion was the absolute value of log 2-fold change (FC) ≥2 and *p*-values < 0.05. Besides, the overlapping lncRNAs in differentially expressed lncRNAs and macrophage associated lncRNAs were defined as DEMlncRNAs.

### Development of DEMlncRNAs signature using machine learning

To identify the survival-related DEMlncRNAs in melanomas, we randomly categorized the melanoma patients in TCGA-SKCM dataset into train or validation samples with a ratio of 7:3. The survival associated DEMlncRNAs were then selected by using univariate Cox regression methodology in train dataset (*p*-values < 0.05). Next, multiple machine learnings contained Least Absolute Shrinkage and Selection Operator (LASSO), Random Forests Variable Selection (RF-VS), and Support Vector Machine-Recursive Feature Elimination (SVM-RFE) were applied to identify the essential DEMlncRNAs in the training dataset. LASSO screened the important features based on the misclassification error, SVM-RFE identified key factors by 5-fold cross-validation errors and RF-VS picked out significant variables by out of bag (OOB) error [24]. The common important DEMlncRNAs were selected out for further analysis. Firstly, the diagnostic model was built by neural network algorithm with these identified DEMlncRNAs. To assess the model’s efficiency, the confusion matrix and Receiver operating characteristic curves (ROC) were utilized. The prognostic risk model was then built using multivariate Cox regression analysis. The following is how the risk score is calculated:


$$\text{Risk}\;\text{score}\;\text{=}\sum_{i=1}^{\text{N}}(coef_{i}\times expr_{i}),$$

where expri means the DEMlncRNA’s relative expression in the risk model, coefi is stand for the DEMlncRNA’s Cox coefficient, and N represents the number of DEMlncRNA. In the train set, the median value was used as a threshold for the DEMlncRNA signature risk score, and samples were divided into high- and low-risk categories. Kaplan-Meier survival curves and log-rank tests were used to compare the prognoses of the high- and low-risk groups. ROC curves and area under the curve (AUC) measurements were also used to evaluate the model’s 3- and 5-year overall survival prediction accuracy. Furthermore, these DEMlncRNAs signatures were verified in the testing dataset and GSE65904 to establish the robustness of the conclusion.

### Pathway enrichment analysis

To investigate the possible involvement of protein-coding genes co-expressed with DEMlncRNAs, the ENCORI website (http://starbase.sysu.edu.cn/index.php) was used. Second, Metascape (http://metascape.org) was utilized to perform functional enrichment of BP and the KEGG terms. In addition, immune-related pathways of DEMlncRNAs were examined in the ImmLnc database (http://bio-bigdata.hrbmu.edu.cn/ImmLnc). Pathways with *p*-value and adjust *p*-value < 0.05 were screened out and regarded as significance.

### Associations between risk score and other prognostic markers

To calculate the relationships between risk score and other prognostic biomarkers, the correlation of risk score with traditional markers (age, clark level, stage.) and previously established prognostic markers included transcriptomic classification (named “immune”, “keratin” and “MITF-low”), mutation subtype (named “BRAF subtype”, “RAS subtype”, “NF1 subtype” and “Triple Wild-Type subtype”) and BRAF mutant were performed [25, 26]. Moreover, uni- and multi-variate Cox regression were performed in three datasets to analyze the prognostic significance of the risk score and traditional clinical factors to evaluate if the risk score is an independent prognostic factor. Next, to explore the association between risk score and immunological microenvironment, the Immuno-Oncology Biological Research (IOBR) approach was used. The CIBERSORT method was initially used to determine the relative proportions of 22 different kinds of infiltrated immune cells in each patient. Only immune cells have 50% value and patients with *P* < 0.05 were considered eligible for further analysis. Afterwards, previous user-built signatures associated with immune microenvironment were evaluated by ssGSEA, Principal Component Analysis (PCA), and Z-score methods. Finally, the associations for risk score with immune cell infiltration and immune-associated markers were studied further.

### Statistical analysis

Every statistical analyses were carried out by R (version 3.6.0) or Python (version 3.8.0) and installed packages. The “glmnet” package performed the LASSO analysis. The “e1017” package was used to carry out the SVM-RFE approach. The “varSelRF” package was applied to the RF-VS algorithm. The “CIBERSORT” program estimated the CIBERSORT deconvolution. The “survival” and “survivalROC” packages were used to perform Kaplan-Meier (KM) survival analyses. The “IOBR” package estimated the IOBR algorithm. Python’s “sklearn” module was used to run the neural network method. The Spearman algorithm was used to evaluate the correlation test. When comparing two groups, a Wilcoxon test was employed; when comparing more than two groups, Kruskal-Wallis tests was utilized. To investigate the relationship between subgroup and clinicopathological variables, a Chi-square test was performed. The Cox regression analysis yielded hazard ratios (HR) and 95 percent confidence intervals (CI). In all tests, *P* < 0.05 was used to denote statistical significance.

### Availability of data and materials

The datasets used and analyzed during the current study are available from the corresponding author on reasonable request.

## RESULTS

### Differentially expressed lncRNA in melanoma

The normalized matrix data of lncRNA for cutaneous melanoma and normal samples (not sun exposed) were obtained from the TCGA-SKCM & GTEx, which included 466 melanomas and 233 normal samples. According to the selection criterion, 1640 differentially expressed lncRNAs were screened out in TCGA-SKCM & GTEx dataset, where 650 lncRNAs were significant up-regulation and 990 lncRNAs were significant down-regulation (Supplementary Figure 2A).

### Identification of validation of prognostic DEMlncRNAs signature

**Figure 2:**
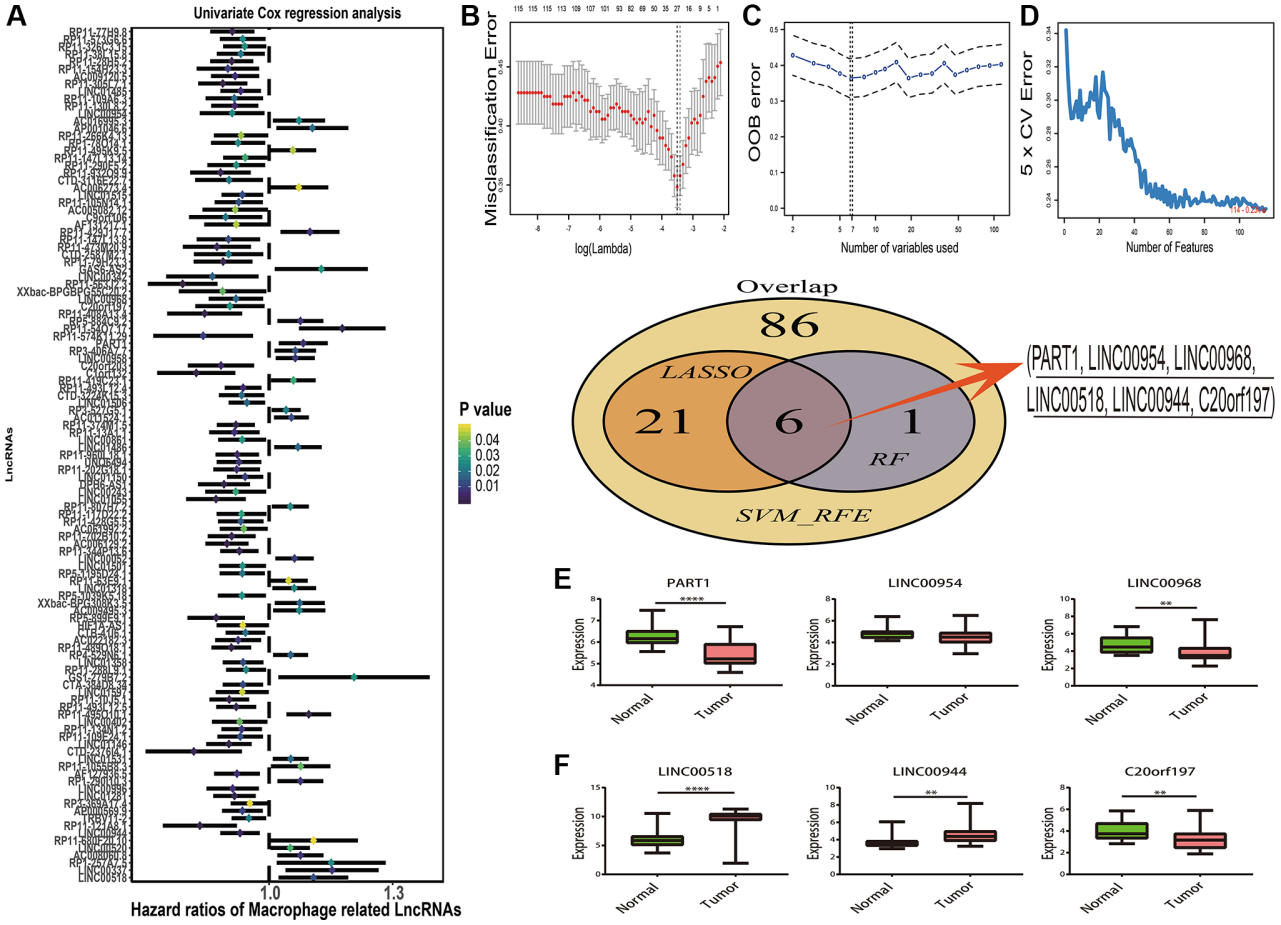
**Prognostic macrophage associated differentially expressed lncRNAs (DEMlncRNAs) feature selection.** (**A**) Forest plots of the prognostic DEMlncRNAs in training dataset. (**B**) Misclassification error distribution with the corresponding λ-logarithm value and the left variants of model in The Least Absolute Shrinkage and Selection Operator (LASSO) algorithm. (**C**) Random forests variable selection (RF-VS) algorithm. The lowest point of the curve indicates the lowest out of bag (OOB) error, and the corresponding DEMlncRNAs at this point are the best signature selected by RF-VS. (**D**) Support Vector Machine-Recursive Feature Elimination (SVM-RFE) algorithms. The point highlighted indicates the lowest error rate, and the corresponding DEMlncRNAs at this point are the best signature selected by SVM-RFE. (**E**) The Venn plot of overlapping DEMlncRNAs selected by LASSO, RF-VS and SVM-RFE algorithms. (**F**) Boxplots of lncRNA expression distribution in GSE15605 dataset.

**Figure 3:**
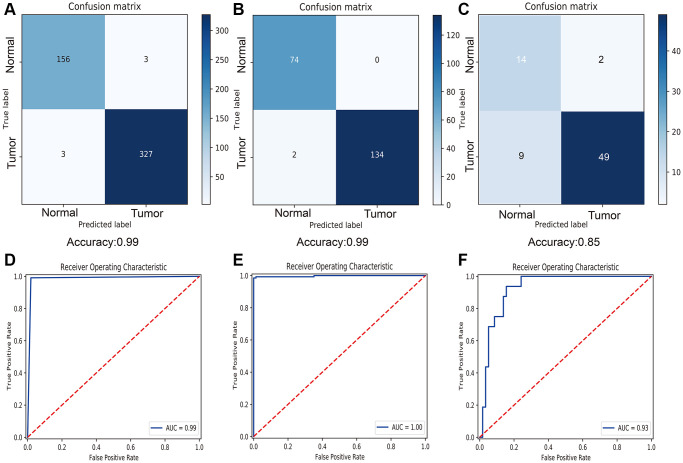
**Diagnostic performance of six selected macrophage associated lncRNAs.** (**A**) Confusion matrix of diagnostic prediction for melanoma in train dataset. (**B**) Confusion matrix of diagnostic prediction for melanoma in test dataset. (**C**) Confusion matrix of diagnostic prediction for melanoma in GSE15605 dataset. (**D**) Receiver operating characteristic (ROC) curve of diagnostic model in train dataset. (**E**) ROC curve of diagnostic model in test dataset. (**F**) ROC curve of diagnostic model in GSE15605 dataset.

According the results of overlap in macrophage associated lncRNAs and differentially expressed lncRNAs (Supplementary Figure 2B), 447 DEMlncRNAs were selected for further analysis (Supplementary Table 2). The heatmap of 447 DEMlncRNAs was shown in Supplementary Figure 2C. After excluding the patients without survival information, the TCGA-SKCM dataset were randomly separated into two independent datasets: train samples (*n* = 313) and validation samples (*n* = 134). Comparison of the clinical information between train and validation samples, no statistically significant differences were observed in two datasets (Table 1). Next, the relationships between 447 DEMlncRNAs and overall survival time in train sample were assessed by univariate Cox regression analysis, and we discovered 115 DEMlncRNAs were significantly correlated with survival (Figure 2A). In order to find the optimum signatures, machine learning methods were firstly performed to screen out the most important DEMlncRNAs in the train dataset. Combined the feature selection results from LASSO algorithm (Figure 2B), RF-VS algorithm (Figure 2C) and SVM-RFE algorithm (Figure 2D), we observed that six overlapping DEMlncRNAs were shared in these machine learning methods (Figure 2E and Supplementary Table 3). Further comparison analysis between 58 melanomas and 16 normal skin samples in the GSE15605 dataset showed that five overlapping DEMlncRNAs had significant differences (Figure 2F). Furthermore, we used neural network algorithm to well establish a diagnostic model with six DEMlncRNAs in TCGA-SKCM & GTEx dataset (randomly split into train and test dataset with ratio = 7:3), which suggested good accuracies and AUC values in train dataset (accuracy = 0.99 and AUC = 0.99) (Figure 3A, 3D) and test dataset (accuracy = 0.99 and AUC = 1.00) (Figure 3B, 3E). Even in GSE15605 dataset, our diagnostic model still revealed an excellent performance to distinguish normal and tumor patients with accuracy = 0.85 and AUC = 0.93 (Figure 3C, 3F).

**Table 1:** Clinical characteristics of train and validation dataset.

	**Level**	**Train samples**	**Validation samples**	***P*-value**
*n*		313	134	
Overall survival time (median (IQR))		3.02 (1.29, 6.48)	3.03 (1.44, 6.26)	0.823
Age (median (IQR))		59.00 (49.00, 72.00)	57.00 (46.00, 68.75)	0.115
Gender (%)	Female	118 (37.7)	53 (39.6)	0.793
	Male	195 (62.3)	81 (60.4)	
Race (%)	Asian	8 (2.6)	4 (3.0)	0.967
	Not reported	7 (2.2)	3 (2.2)	
	White	298 (95.2)	127 (94.8)	
Melanoma clark level value (%)		97 (31.0)	42 (31.3)	0.780
	I	1 (0.3)	0 (0.0)	
	II	10 (3.2)	8 (6.0)	
	III	55 (17.6)	21 (15.7)	
	IV	116 (37.1)	48 (35.8)	
	V	34 (10.9)	15 (11.2)	
Pathologic_M (%)		20 (6.4)	6 (4.5)	0.473
	M0	275 (87.9)	123 (91.8)	
	M1	18 (5.8)	5 (3.7)	
Pathologic_N (%)		17 (5.4)	2 (1.5)	0.151
	N0-1	225 (71.9)	103 (76.9)	
	N2-3	71 (22.7)	29 (21.6)	
Pathologic_T (%)		21 (6.7)	6 (4.5)	0.634
	T0-1	77 (24.6)	32 (23.9)	
	T2-4	215 (68.7)	96 (71.6)	
Tumor stage (%)	I/II nos	4 (1.3)	6 (4.5)	0.367
	Not reported	26 (8.3)	10 (7.5)	
	Stage I	51 (16.3)	25 (18.7)	
	Stage II	97 (31.0)	41 (30.6)	
	Stage III	118 (37.7)	47 (35.1)	
	Stage IV	17 (5.4)	5 (3.7)	
Tumor status (%)		121 (38.7)	50 (37.3)	0.851
	Tumor free	122 (39.0)	56 (41.8)	
	With tumor	70 (22.4)	28 (20.9)	
Radiation therapy (%)		123 (39.3)	49 (36.6)	0.275
	No	169 (54.0)	70 (52.2)	
	Yes	21 (6.7)	15 (11.2)	
Vital status (%)	Alive	169 (54.0)	65 (48.5)	0.190
	Dead	144 (46.0)	68 (50.7)	
	Not reported	0 (0.0)	1 (0.7)	
Sample tissue (%)	Metastatic	246 (78.6)	100 (74.6)	0.426
	Primary tumor	67 (21.4)	34 (25.4)	

Abbreviations: IQR: interquartile range; OS: overall survival.

**Figure 4:**
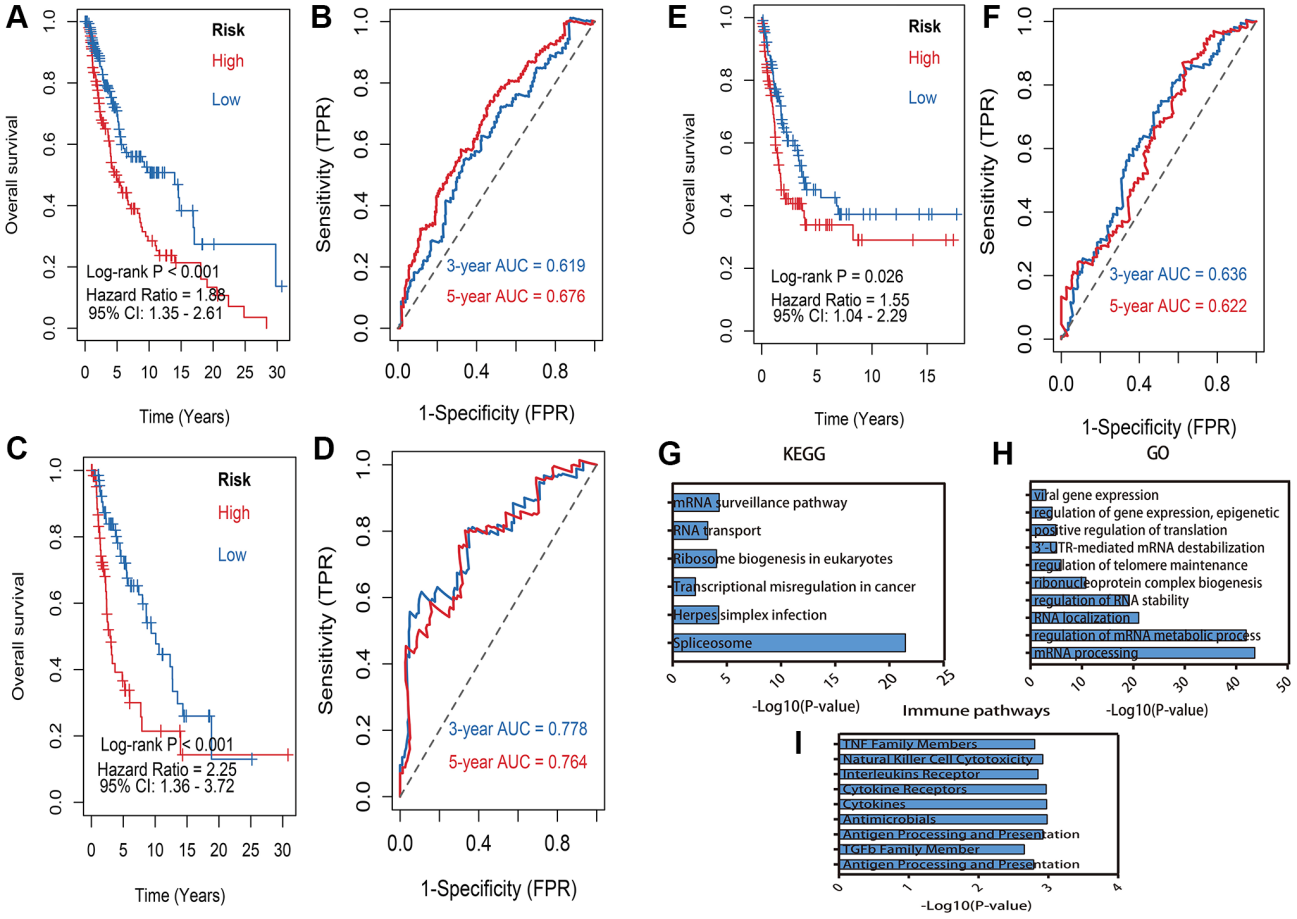
**Kaplan–Meier survival analysis and pathway enrichment.** (**A**) Kaplan–Meier survival analysis of risk model in train dataset. (**B**) 3, 5 years of the receiver operating characteristic (ROC) curves in train dataset. (**C**) Kaplan–Meier survival analysis of risk model in validation dataset. (**D**) 3, 5 years of the receiver operating characteristic (ROC) curves in validation dataset. (**E**) Kaplan–Meier survival analysis of risk model in GSE65904 dataset. (**F**) 3, 5 years of the receiver operating characteristic (ROC) curves in GSE65904 dataset. (**G**) Enriched Kyoto Encyclopedia of Genes and Genomes (KEGG) pathways of lncRNA-related mRNAs: X-axis means the -log10(*p*-value) of enrichment, Y-axis means pathway terms. (**H**) The biology process (BP) pathway enrichment of lncRNA-related: X-axis means the -log10(*p*-value) of enrichment, Y-axis means pathway terms. (**I**) The immune related pathway enrichment: X-axis means the -log10(*p*-value) of enrichment, Y-axis means pathway terms.

Afterwards, the six DEMlncRNAs were subsequently performed to develop a risk model in train dataset by applying multivariate Cox analysis. Each patient’s risk score was finally computed using the risk score formula. The risk model consisted of risk scores distribution, vital status, overall survival (OS) time, and heat map of DEMlncRNAs expression, which respectively illustrated in train (Supplementary Figure 3A), validation (Supplementary Figure 3B) and GSE65904 (Supplementary Figure 3C) datasets. Next, we divided the melanoma patients in the train dataset into high-risk and low-risk groups based on the median value of the risk score. With a significant log-rank test of *p* < 0.001, the KM survival curves revealed that patients of high-risk group had substantially worse survival than low-risk group (Figure 4A). The ROC curve was drawn to estimate the ability of prediction power, and the AUCs of the curves were 0.619 at 3-year and 0.676 at 5-year respectively (Figure 4B). Moreover, to confirm the robustness of the six DEMlncRNAs based model, it was further verified in validation and GSE65904 datasets via the same cutoff value. The patients in validation and GSE65904 datasets were accordingly divided into high- and low-risk subgroups. KM curves indicated that significantly distinct survival outcome was also observed at high- and low-risk group in validation dataset with log-rank *p* < 0.001 (Figure 4C) and GSE65904 dataset with log-rank *p* = 0.026 (Figure 4E). The 3-, 5-year of AUCs were 0.778, 0.764 in validation dataset (Figure 4D) and 0.636, 0.622 (Figure 4F) in GSE65904 dataset respectively.

### Independence of the risk score from traditional clinical variables

**Figure 5:**
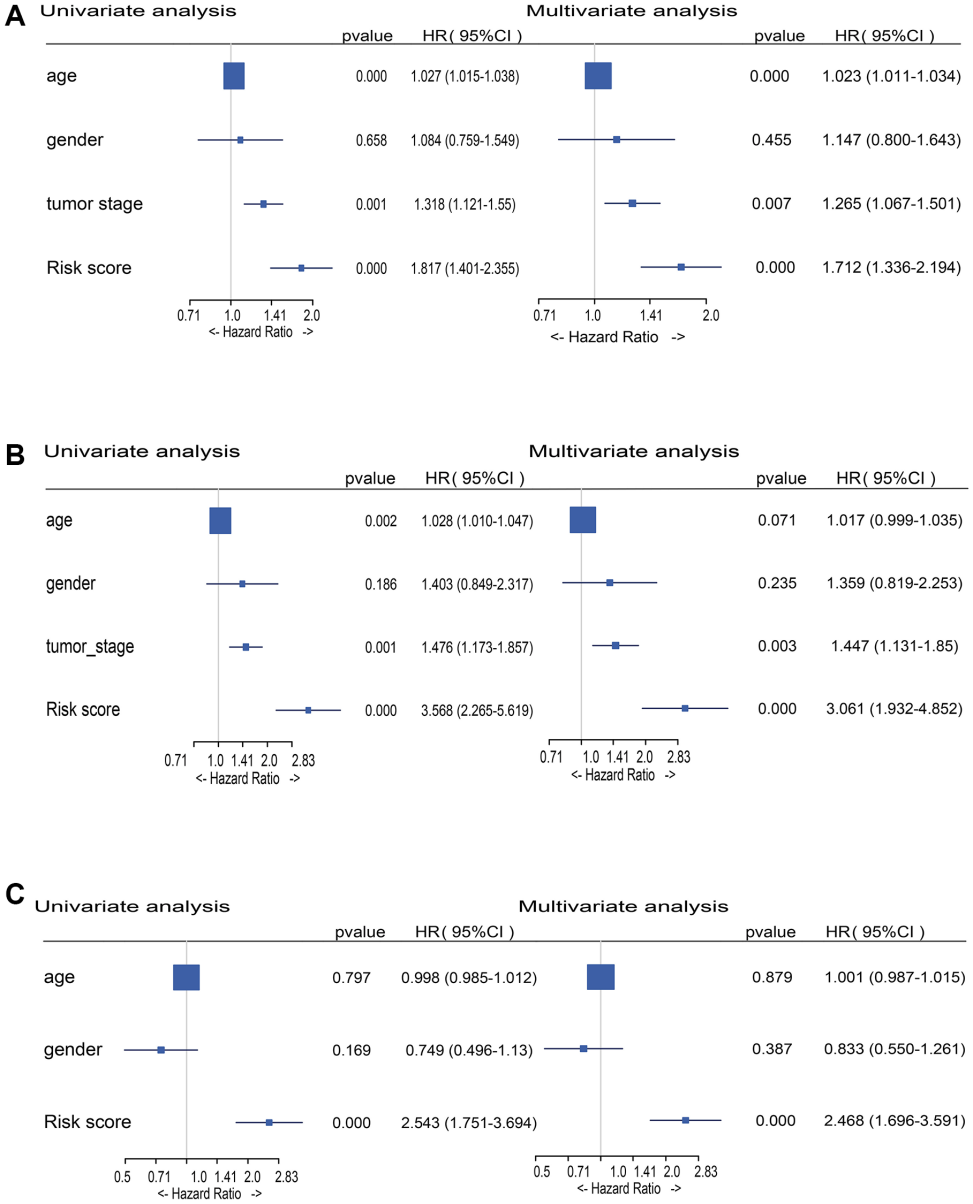
**Forest plot of risk score and traditional clinical variables in uni- and multivariate Cox regression.** (**A**) Hazard Ratios (HRs) of uni- and multivariate Cox analysis in TCGA-SKCM train dataset. (**B**) HRs of uni- and multivariate Cox analysis in TCGA-SKCM validation dataset. (**C**) HRs of uni- and multivariate Cox analysis in GSE65904 dataset.

In uni- and multi-variate Cox regression analysis, the risk score and conventional clinical parameters were utilized to establish if the DEMlncRNAs signature is an independent predictive predictor of OS in various datasets. The train and validation datasets demonstrated that the DEMlncRNAs signature risk score and tumor stage were substantially linked with OS in both uni- and multi-variate Cox analysis (Figure 5A, 5B). In same analyses of an external GSE65904 dataset, the risk score was remained strongly linked with OS (Figure 5C). These findings suggest that the risk score of the DEMlncRNAs signature is a significant predictive factor that is independent of other conventional clinical factors.

### Pathway enrichment analysis

Overall, 53 lncRNA-paired protein-coding genes were predicted on the ENCORI website and utilized in BP term and KEGG pathway enrichment analyses. The KEGG enrichment results showed that these paired protein-coding genes were considerably enriched in biological processes linked to RNA transport, the mRNA surveillance pathway, and ribosome synthesis in eukaryotes, transcriptional misregulation in cancer, Herpes simplex infection and Spliceosome (Figure 4G). The BP findings indicated that these paired protein-coding genes were considerably enriched in pathways such as viral gene expression, gene expression regulation, epigenetics, positive control of translation, and 3′-UTR-mediated mRNA destabilization, regulation of telomere maintenance (Figure 4H). Furthermore, Based on ImmLnc database, these DEMlncRNAs significantly associated with immune pathways, including TNF family members, natural killer cell cytotoxicity, interleukins receptor and cytokine Receptors (Figure 4I). The detail results of pathway enrichment analysis were listed in Supplementary Table 4.

### Associations between risk score and conventional variables as well as the immunological microenvironment

**Figure 6:**
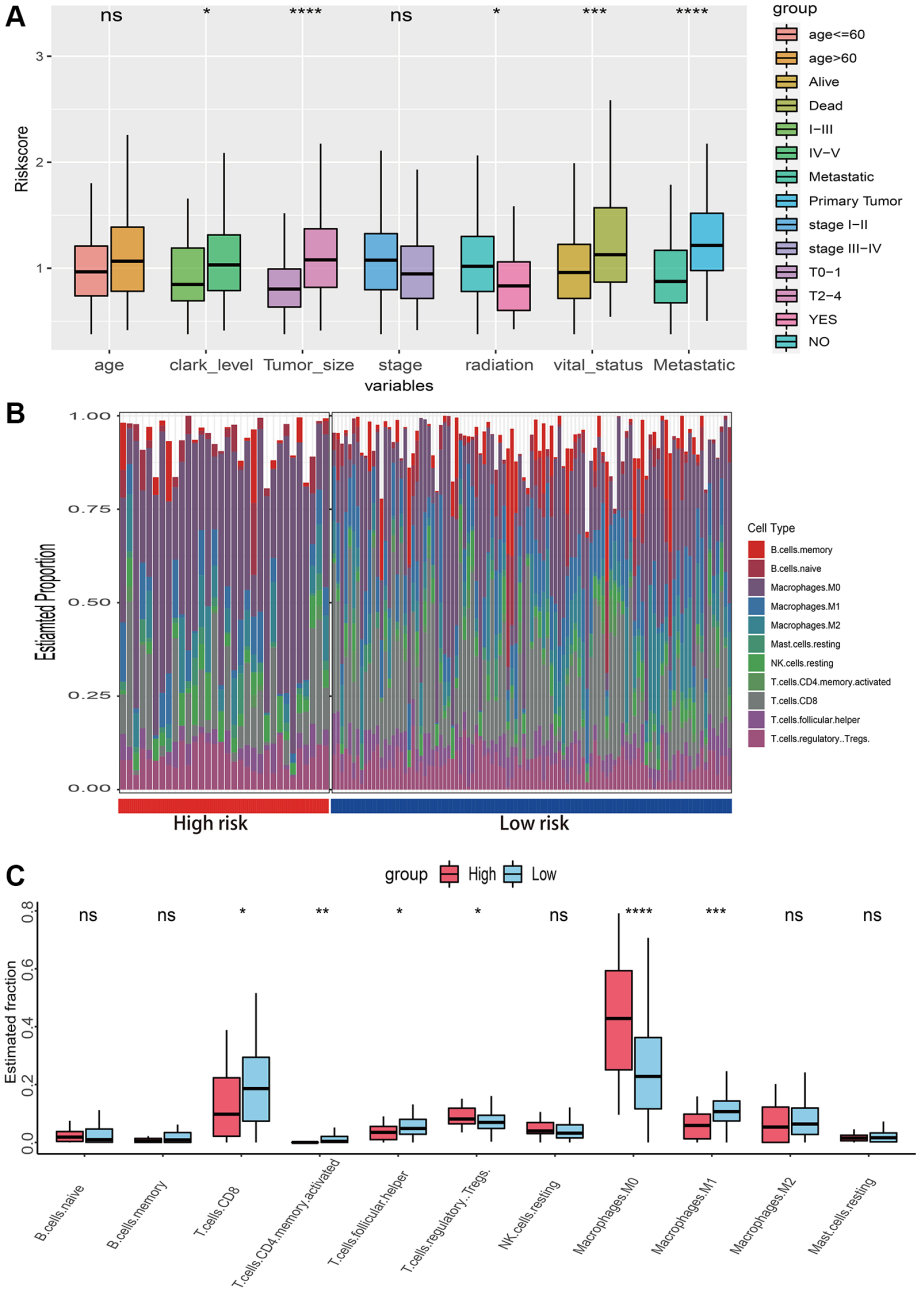
**Relationships between risk score with clinical characteristics and immune microenvironment.** (**A**) The risk score distribution of clinical variables which including age, clark level, tumor size, stage, radiation, vital status and metastatic status. (**B**) The landscape of immune infiltration between high and low risk groups in TCGA-SKCM dataset. (**C**) The difference of 11 immune infiltration between high and low risk groups; ^*^p < 0.05; ^**^p < 0.01; ^***^p < 0.001; ^****^p < 0.0001.

**Figure 7:**
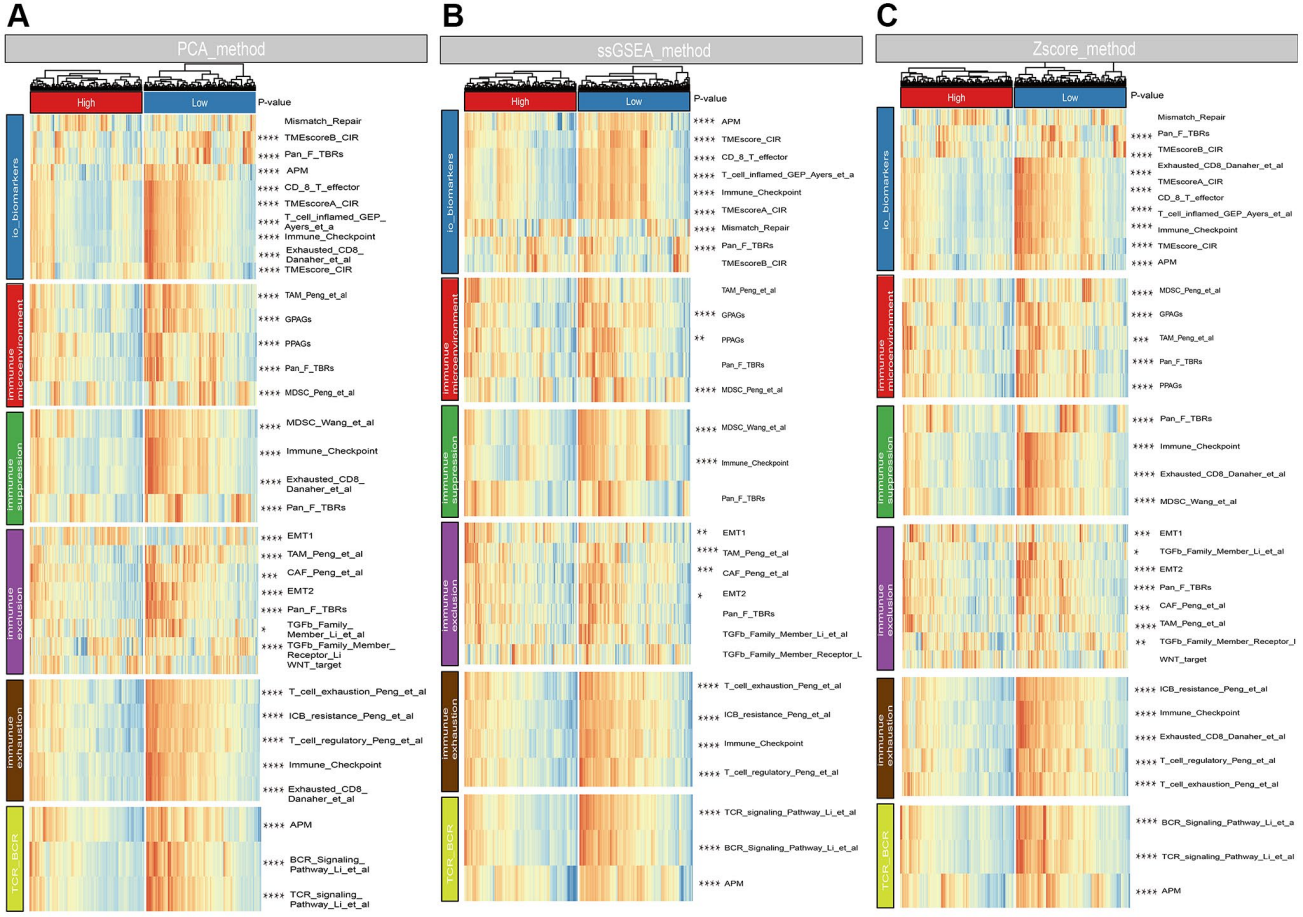
**Associations between risk score and immune signatures.** (**A**) Heatmap for immune related signatures between high and low risk subgroup based on Principal Component Analysis (PCA) method. (**B**) Heatmap for immune related signatures between high and low risk subgroup based on Single-sample Enrichment Analysis (ssGSEA) methods. (**C**) Heatmap for immune related signatures between high and low risk subgroup based on Z-score method.

Box plots demonstrated that clark level, tumor size, radiation, vital status, and metastatic status were associated with risk score (Figure 6A). To investigate the relationships between risk score and immunological microenvironment, the CIBERSORT algorithm was employed to calculate the relative proportions of 22 immune cells. Only 133 melanoma samples and 11 immune cells were selected out for further research based on the selection criteria. In overall, the 11 immune cell subsets in melanoma included memory B cells, naïve B cells, resting Mast cells, and M0, M1, M2 macrophages, resting NK cells, CD 8 T cells, follicular helper T cells, regulatory Tregs T cells and CD4 memory T cells have been activated, whose sum of mean proportions was more than 75% in all melanoma samples (Figure 6B). The differences in immune cell infiltrations between high- and low-risk groups were explored, and the findings revealed that Macrophages M0, M1, CD8, follicular helper, regulatory Tregs and CD4 memory activated T cells were differently infiltrated (Figure 6C). Afterwards, a total of 25 promising immune associated signatures were scored and classified into six categories which contained immune related biomarker, immune microenvironment, immune suppression, immune exclusion, immune exhaustion, T-cell or B-cell receptor (TCR_BCR). The heatmaps delineated a different immune expressed pattern between high and low risk phenotype regardless by using PCA (Figure 7A), ssGSEA (Figure 7B) or Z-score (Figure 7C) methods.

### Comparison of risk score and previously identified prognostic indicators

**Figure 8:**
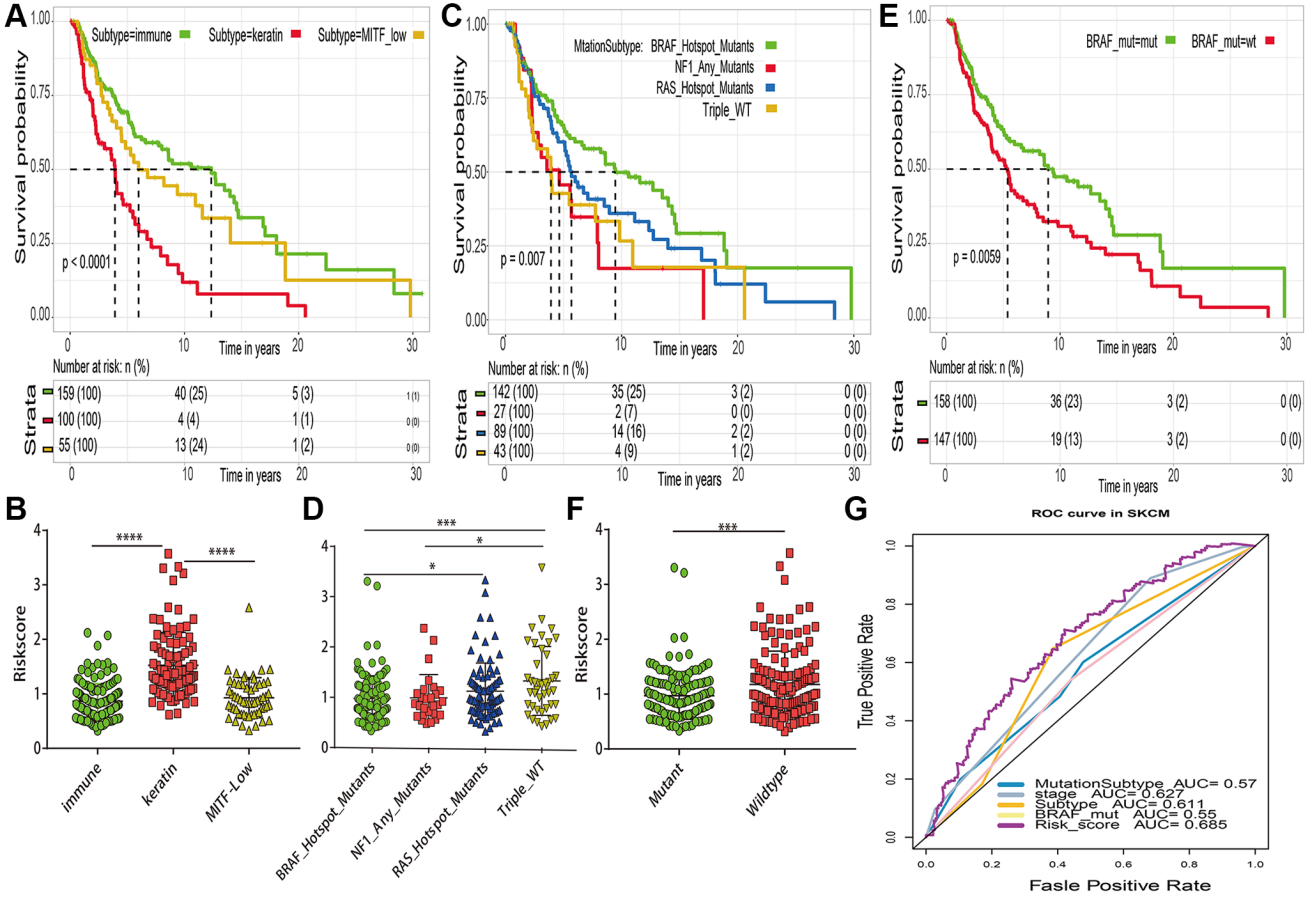
**Comparations between risk score and previously established prognostic markers in TCGA-SKCM.** (**A**) Kaplan–Meier survival curve of transcriptomic subtypes. (**B**) The risk score distribution of transcriptomic subtypes (named “immune”, “keratin” and “MITF-low). (**C**) Kaplan–Meier survival curve of mutation subtypes (named BRAF subtype, RAS subtype, NF1 subtype and Triple Wild-Type subtype). (**D**) The risk score distribution of mutation subtypes. (**E**) Kaplan–Meier curve of BRAF mutant. (**F**) The risk score distribution of BRAF mutant and wildtype. (**G**) The 5 years area under the curve (AUC) of risk score and prognostic markers associated with overall survival. ^*^p < 0.05; ^**^p < 0.01; ^***^p < 0.001; ^****^p < 0.0001.

In order to correlate our assessment of risk score with other established prognostic markers, several well established prognostic markers such as transcriptomic classification, mutation subtype and BRAF mutant for cutaneous melanoma were obtained from the previous TCGA study [25]. The TCGA research proven that patient in “immune” subtype of transcriptomic classifications (Figure 8A), “BRAF Hotspot Mutants” of mutation subtypes (Figure 8C) and BRAF mutant group (Figure 8E) have a good prognosis, respectively. The risk distribution of these well established prognostic markers suggested that “MITF low” subtype (Figure 8B), “Triple WT” mutant subtype (Figure 8D) and BRAF wildtype group (Figure 8F) have a higher risk score than “immune” subtype, “BRAF Hotspot Mutants” subtypes and BRAF mutant group, respectively. Most importantly, compared with the 5 years AUC values of these established prognostic markers (transcriptomic classification, mutation subtype, tumor stage and BRAF mutant), our signature can achieve higher accuracy value (Figure 8G).

### Potential indicator for melanoma immunotherapy

**Figure 9:**
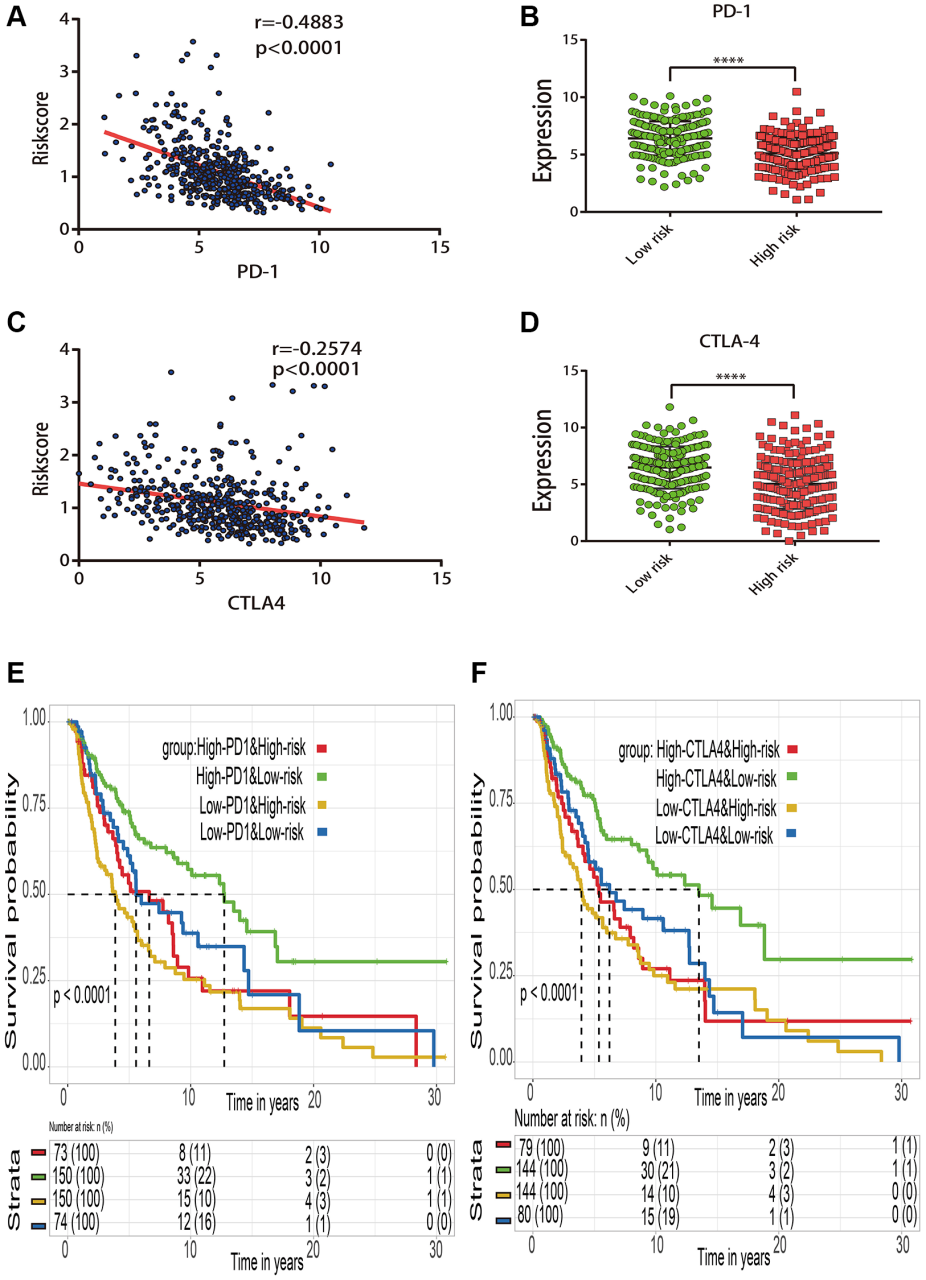
**Effect of the risk score and immune checkpoint gene expression on patient survival.** (**A**) Correlation between the risk score and PD-1 gene expression. (**B**) Expression distribution of PD-1 gene in the high- and low-risk groups stratified by risk score. (**C**) Correlation between the risk score and CTLA4 gene expression. (**D**) Expression distribution of CTLA4 gene in the high- and low-risk groups stratified by risk score. (**E**) Kaplan–Meier survival curve of four groups stratified by the risk score and PD-1 expression. (**F**) Kaplan–Meier survival curve of four groups stratified by the risk score and CTLA4 expression.

The relationship between risk score and immune checkpoint gene expression (PD-1 and CTLA-4) was investigated further to assess the potential responsiveness to immunotherapy. According to the Spearman tests, the estimated risk score was substantially negatively linked with PD-1 expression (r = −0.4883; *p* < 0.0001) (Figure 9A) and CTLA-4 expression (r = −0.2574; *p* < 0.0001) (Figure 9C). The stratified analysis indicated that PD-1 (Figure 9B) and CTLA-4 (Figure 9D) in the low risk subgroup had significant higher expression levels than those in high risk subgroup. The cross-talk influences of the risk score and immune checkpoint genes on patient survival were further analyzed. Based on the combination of risk score and immune checkpoint genes, these patients were classified into four categories, and KM curve analyses were performed to assess the various survival outcomes among the four subgroups. When compared to the data, the risk score was able to discriminate patients’ outcomes with opposing levels of immune checkpoint genes (Figure 9E, 9F). Patients with high risk scores and low levels of immune checkpoint genes fared the poorest. Patients with low risk scores and high levels of immune checkpoint genes, on the other hand, are more likely to survive longer among the four groups.

**Figure 10:**
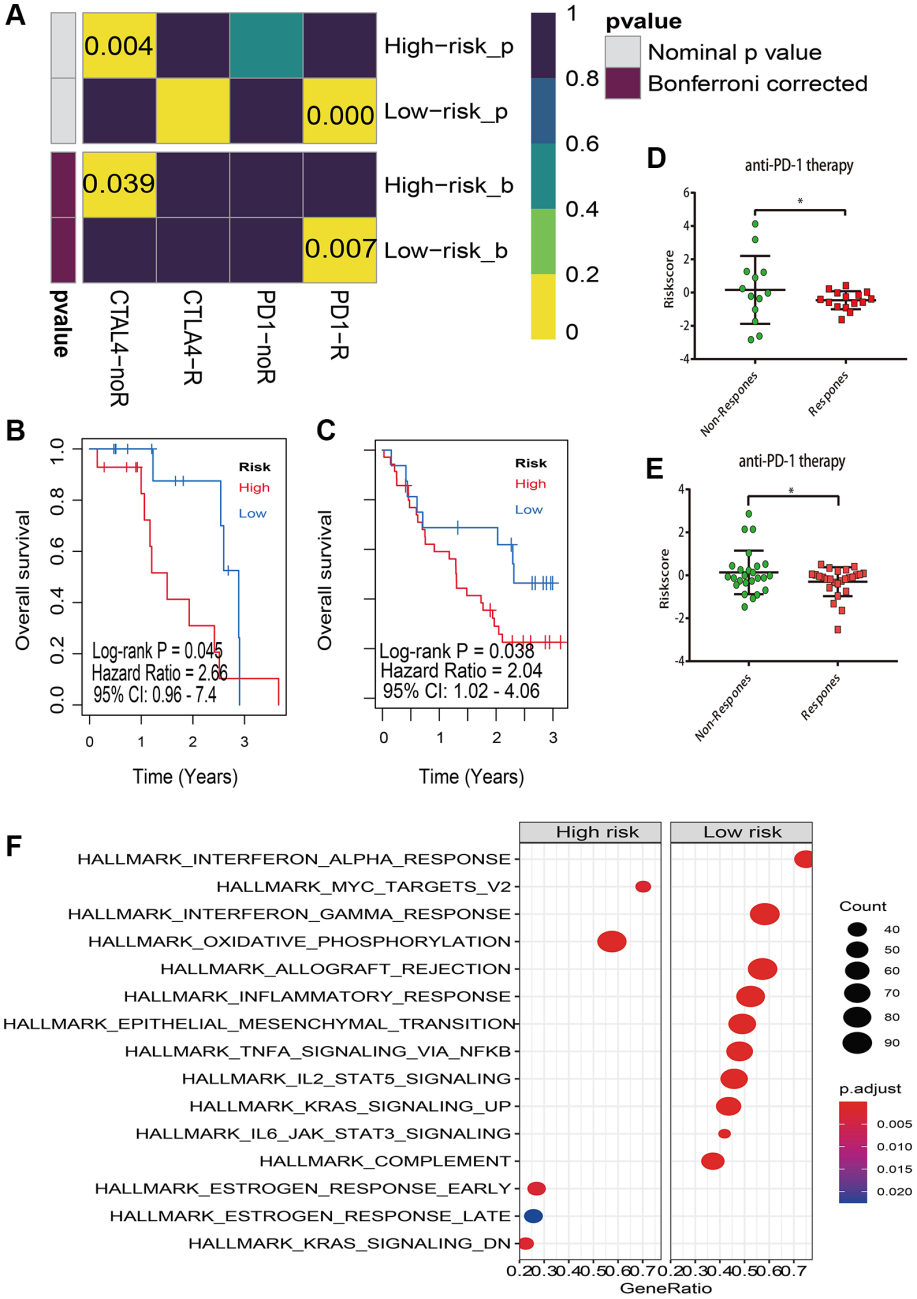
**Immunotherapy response prediction.** (**A**) Submap analysis manifested that low risk group could be more sensitive to the PD-1 inhibitor (Bonferroni-corrected *P* = 0.07), and high risk group are insensitive to anti-CTLA-4 therapy (Bonferroni corrected *P* = 0.039); (**B**) Kaplan–Meier survival analysis of risk model in Hugo et al melanoma study (accession number: GSE78220); (**C**) Kaplan–Meier survival analysis of risk model in Riaz et al melanoma study (accession number: GSE91061); (**D**) The risk score distribution of anti-PD-1 therapy between response and non-response in GSE78220; (**E**) The risk score distribution of anti-PD-1 therapy between response and non-response in GSE91061; (**F**) Gene set enrichment analysis (GSEA) of high vs low risk scores groups in cancer hallmark pathways.

Because of the strong association between the risk score and immune checkpoint genes, we postulated that the risk score may be used to predict response to melanoma immunotherapy. As a consequence, we conducted the TIDE method and subclass mapping to predict melanoma who responded to immune-checkpoint inhibitors (CTLA-4 and PD-1) [22]. Strikingly, the low risk group reacts better to anti-PD-1 therapy (Bonferroni adjusted *P* = 0.007) (Figure 10A). Patients in the high-risk category, on the other hand, do not respond to anti-CTLA-4 therapy (Bonferroni corrected *P* = 0.039) (Figure 10A). To validate our hypothesis, participants in the Riaz et al. and Hugo et al. melanoma studies who received anti-PD-1 treatment were divided into low and high risk score groups [20, 21]. Notably, individuals in high risk group have a poor prognosis regardless in Hugo et al. cohort (Figure 10B) or Riaz et al. cohort (Figure 10C). Furthermore, in the Hugo et al cohort (Figure 10D) and Riaz et al. cohort (Figure 10E), the non-respond group had a higher risk score than the response group for anti-PD-1 immunotherapy.

### Gene set enrichment analysis (GSEA)

GSEA was used to examine the distinct activation of cancer hallmarks between low and high risk groups for investigating the potential biological and molecular mechanism of the lncRNA signature. We found that 10 positive linked pathways were enriched in the low risk group, which included interferon alpha and gamma response, allograft rejection, inflammatory response, epithelial mesenchymal transition, TNFA signaling through NFKB (Figure 10F). Oxidative phosphorylation, MYC targets, estrogen response late, estrogen response early, and KRAS signaling were all actively related with the high risk group (Figure 10F).

## DISCUSSION

The risk and morbidity of melanoma patients increase rapidly. Once it has spread, it is much harder to improve the chance of melanoma-specific survival at all [27]. Cancer immunotherapy is currently recognized as a potential treatment approach that is commonly employed in melanoma patients [28, 29]. Nevertheless, not all melanomas are responding to immunotherapy and even in the subtype of those tumor. These differential outcomes not only caused by the heterogeneity of tumor, but also resulted in tumor microenvironment composition [30, 31]. Immune cells of the tumor microenvironment are critically involved in response of immunotherapy. As a key component of infiltrating immune cells, macrophage has been identified that it’s activities could dysregulate immune activity, enhance tumor metastasis, upregulate immune response evasion and effect other immune cells [32–34]. Besides, recent studies revealed that lncRNAs are widely regulated cancer immunity. Several bigdata analyses suggested that the cell-type specificity of lncRNA can work as a potential marker for the subpopulation of immune cells [35, 36]. Hence, it is necessary to elucidate the accurate molecular regulation of macrophage polarization to increase the efficacy of tumor-infiltrated macrophage targeted methods and immunotherapy response in melanoma.

Therefore, in this study, multiple machine learning algorithms were applied to select potential lncRNAs from the list of macrophage associated lncRNAs and established a diagnostic and prognostic signature, which also was verified in validation and external sets. The diagnostic and prognostic signature contained six lncRNAs, including PART1, LINC00968, LINC00954, LINC00944, LINC00518 and C20orf197. Among these macrophage-related lncRNAs, several have been demonstrated to link with cancer or immunological response. For example, the PART1 lncRNA can modulate Toll-like receptor pathways to effect the immune response and cell apoptosis [37]. Recent experimental results manifested that the inhibition of LINC00968 can significantly decrease the proliferation of tumor cell and could be regard as an oncogene in various cancers [38, 39]. Luan at el also suggested that LICN00518 was highly expressed in melanoma tissues and could be an independent risk indicator for melanoma patients [40]. The enrichment of these lncRNAs in immune response-related pathways, such as TNF family members, natural killer cell cytotoxicity, and interleukin receptors, suggests their potential involvement in modulating the interactions between tumor-infiltrating macrophages and the immune microenvironment within melanoma. These lncRNAs may exert regulatory effects on the expression of key immune-related genes, cytokines, and receptors, thereby influencing the polarization and function of macrophages and other immune cells in the tumor microenvironment. Furthermore, the association of these lncRNAs with cytokine receptors and RNA synthesis-related signaling pathways, including mRNA surveillance, RNA transport, and transcriptional misregulation in cancer, indicates their potential roles in coordinating the expression and processing of immune-related genes and signaling molecules. It is conceivable that these lncRNAs may participate in the post-transcriptional and post-translational regulation of immune response genes, affecting the secretion and activity of cytokines and chemokines involved in melanoma-immune cell crosstalk.

To estimate the accurate ability of survival prediction, we also built risk score model and classified melanomas into high- or low-risk group. The KM survival curves showed that low-risk patients have a good prognosis. Compared with the traditional clinical factors, the multivariate Cox results in different cohorts suggested that the risk score might be independently considered as a prognostic biomarker. Moreover, the distribution of risk score in clinical features manifiested that the high risk score was closely associated with high clark level, large tumor size, no radiation and metastasis. Besides, the high-risk patients had higher macrophage infiltration and lower T cell infiltration than low risk group. T cells act as cytotoxic lymphocytes, and once fully cytotoxic, they are critical in suppressing the proliferation of cancer cells and growth through the immune system [41]. However, the increasingly infiltrated myeloid cells such as macrophages, and dendritic cells would enhance cancer cells expansion, immune surveillance evasion, and eventually lead to metastasis [42, 43]. Most importantly, we astonishingly observed that our macrophage associated lncRNAs signature defined low-risk melanoma patients who were belong to immune inflamed subtype and have strong immuno-stimulating functions of suppression, exclusion, and exhaustion. Therefore, it goes without saying that our lncRNA related signature closely associated with immune activation and prognosis of melanoma. Furthermore, the risk score distributions of transcriptomic classification, mutation subtype and BRAF mutant showed that “keratin”, “Triple WT” subtypes and wildtype of BRAF were significantly higher than “immune”, “BRAF Hotspot Mutants” and mutant of BRAF in melanoma, which was in line with prior findings. Notably, when compared to the AUC values of well-established prognostic biomarkers such as stage, transcriptome categorization, mutation subtype, and BRAF mutant over a 5-year period, our lncRNA associated biomarker achieves a higher accuracy value.

At present, checkpoint blockade immunotherapies were emerging as a promising strategy and reveal a great benefit in cancer therapy. Especially, PD-1 and CTLA-4 blocking antibodies are widely used in clinical melanoma treatment [44–46]. However, only a portion of patients are responding to immune checkpoint inhibitor therapy. Therefore, it’s essentially to identify the predicted roles for immune checkpoint immunotherapy responses. When checking the associations between DEMlncRNAs signature and PD-1/CTLA-4, the risk score of DEMlncRNAs signature showed a significantly correlated with the PD-1 and CTLA-4 expression. In addition, the combination of survival analyses between DEMlncRNAs signature and immune checkpoint genes indicated an interacted influence on the prognosis of patients. Our results are consistent with recent researches that the PD-1 expression of tumor-infiltrating macrophage negatively correlates with the function of macrophage for against tumor cells [47]. The synthesis of PD-1 blockade with macrophage associated treatment will enhance immunotherapy effect in cancer therapy [48]. Hence, our DEMlncRNAs related signature might be used to predict melanoma treatment response. However, it is uncertain whether types of melanomas are amenable to immune checkpoint inhibitor treatment. Thus, the subgroup with varied risk scores was investigated in many published datasets that reacted to immune-checkpoint inhibitors. We were surprised to discover that the low-risk group is likely to respond to anti-PD-1 treatment, whereas the high-risk group is insensitive to anti-CTLA-4 treatment, which could supply effective solutions to aid in the final clinical decision and help patients with advanced melanoma achieve the highest remission rate.

Moreover, we discovered that immunological pathways such interferon alpha and gamma response, inflammatory response, allograft rejection, and TNFA signaling through NFKB were favorably active in the low-risk phenotype using GSEA. Interferon alpha and gamma signaling is well recognized as an important effector in anti-cancer immune response [49]. NFKB is essential for the control of immunological responses and inflammation [50]. As a result, it’s clear to see why low-risk melanoma patients have a greater survival rate and respond better to immunotherapy.

## CONCLUSION

In summary, our study comprehensively identified several lncRNAs related signatures by integrative analysis of immune related lncRNA, tumor-infiltrating macrophage and clinical features. And we also proven the efficiency of signature in predicting prognosis and immunotherapy response of melanoma, which may give a more simple and reliable prediction for melanoma patients and provide a framework to evaluate potential population for immunotherapy. Furthermore, to the best of our knowledge, this is the first study to investigate the tumor-infiltrated macrophage associated lncRNA signature, emphasizing the role of lncRNAs in macrophage infiltration and paving the way for future individual melanoma immunotherapy.

## Supplementary Materials

Supplementary Figures

Supplementary Table 1

Supplementary Table 2

Supplementary Table 3

Supplementary Table 4
