# Supplementary material for: Tumor-infiltrating macrophage associated lncRNA signature in cutaneous melanoma: implications for diagnosis, prognosis, and immunotherapy
Source: Aging (Albany NY). 2024 Mar 13;16(5):4518–40. doi: 10.18632/aging.205606 (PMC10968696; doi:10.18632/aging.205606)
Supplement: Supplementary Figures [file aging-16-205606-s001.pdf]

SUPPLEMENTARY FIGURES

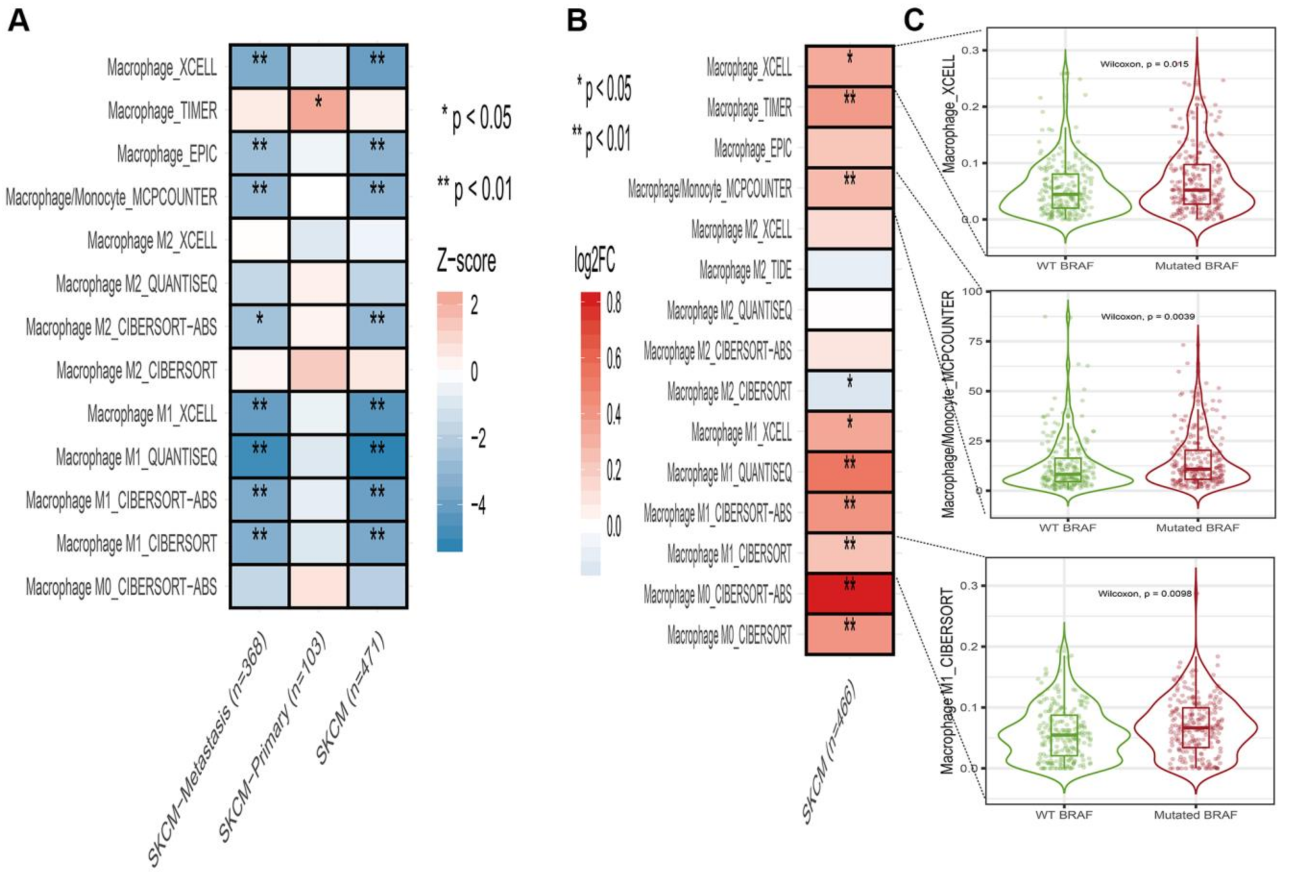

Supplementary Figure 1. The prognostic value of macrophage in TCGA-SKCM by multiple immune deconvolution methods. (**A**) Heatmap of multivariable Cox proportional hazard model for macrophage in TCGA-SKCM datasets. (**B**) Heatmap of correlation between macrophage and BRAF mutant. (**C**) Boxplot of macrophage between BRAF mutant and BRAF wildtype.

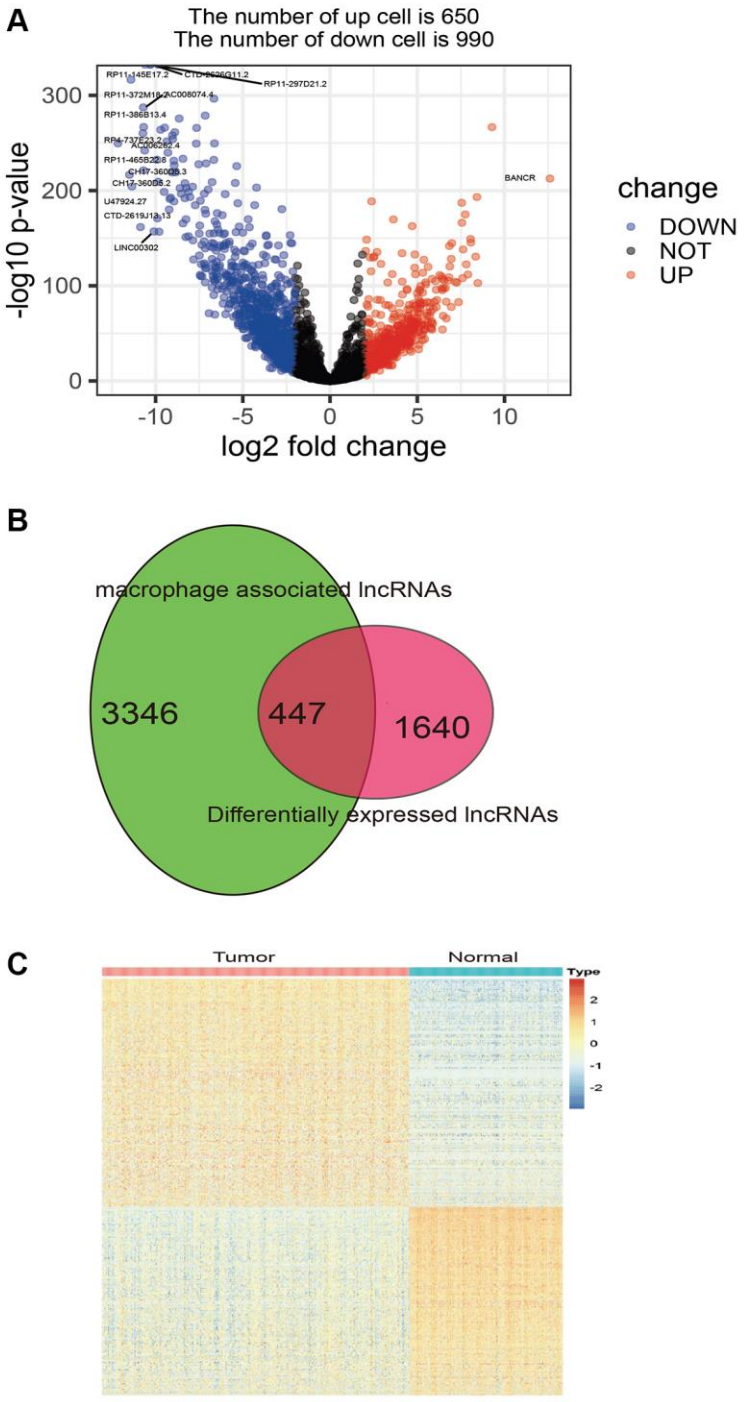

Supplementary Figure 2. Selection of macrophage associated differentially expressed lncRNAs (DEMlncRNAs). (**A**) Volcano plot of differentially expressed lncRNAs between normal and melanoma. The highly differentially expressed lncRNAs were labeled (|Log2FC|>10). (**B**) The Venn plot of macrophage associated differentially expressed lncRNAs (DEMlncRNAs). (**C**) Heat map of the DEMlncRNAs in TCGA-SKCM dataset.

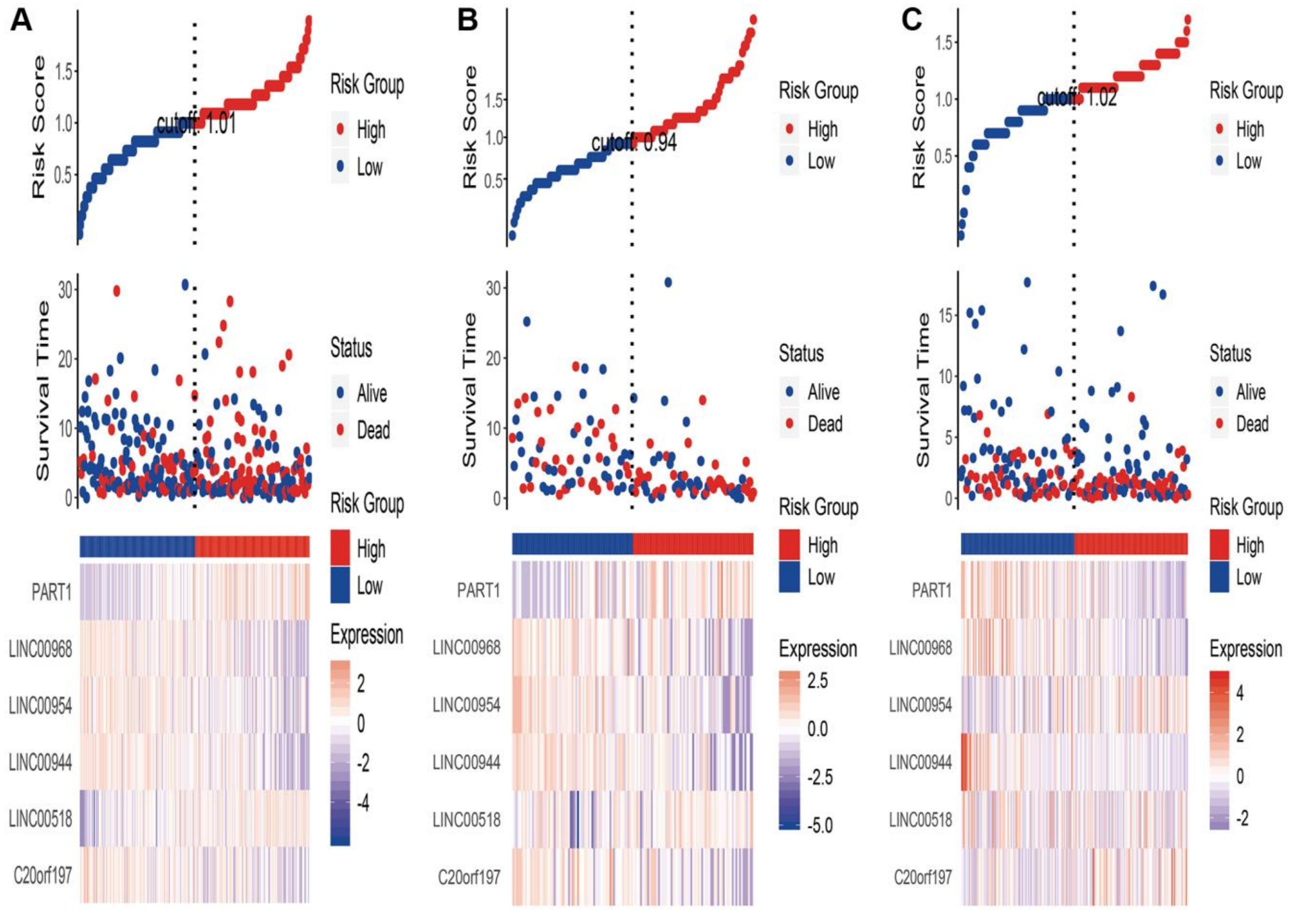

Supplementary Figure 3. Risk score model. (**A**) The risk model in train dataset: the distribution of risk score which are arranged in ascending order from left to right; Overall survival (OS) time and life status; The prognostic lncRNA expression patterns. (**B**) The risk model in validation dataset: the distribution of risk score which are arranged in ascending order from left to right; Overall survival (OS) time and life status; The prognostic lncRNA expression patterns. (**C**) The risk model in GSE65904 dataset: the distribution of risk score which are arranged in ascending order from left to right; Overall survival (OS) time and life status; The prognostic lncRNA expression patterns.
