# Supplementary material for: Tumor-infiltrating macrophage associated lncRNA signature in cutaneous melanoma: implications for diagnosis, prognosis, and immunotherapy
Source: Aging (Albany NY). 2024 Mar 13;16(5):4518–40. doi: 10.18632/aging.205606 (PMC10968696; doi:10.18632/aging.205606)
Supplement: Supplementary Table 1 [file aging-16-205606-s002.docx]

| Supplementary Table 1. The correlation between macrophage and lncRNAs in cutaneous melanoma. | | | | | | | | | |
| --- | --- | --- | --- | --- | --- | --- | --- | --- | --- |
| **cancer** | **immune_cell** | **lncRNA_id** | **lncRNA_symbol** | **cor_p_value** | **cor_R_value** | **lncRNA_id** | **lncRNA_symbol** | **cor_p_value** | **cor_R_value** |
| SKCM | Macrophage | ENSG00000146521 | LINC01558 | 4.49E-08 | 0.2492656 | ENSG00000254038 | RP11-419C23.1 | 0.0264942 | -0.1024624 |
| SKCM | Macrophage | ENSG00000152931 | PART1 | 0.011514 | -0.1165855 | ENSG00000254054 | RP11-156K13.3 | 9.70E-10 | 0.2775038 |
| SKCM | Macrophage | ENSG00000153363 | LINC00467 | 9.45E-08 | -0.2433776 | ENSG00000254211 | LINC01485 | 0.0107529 | 0.1176816 |
| SKCM | Macrophage | ENSG00000175746 | C15orf54 | 0.000166 | 0.1730214 | ENSG00000254223 | RP11-419K12.1 | 3.48E-06 | -0.2123823 |
| SKCM | Macrophage | ENSG00000176659 | C20orf197 | 6.78E-21 | 0.4144753 | ENSG00000254319 | RP11-134O21.1 | 6.80E-08 | 0.2459941 |
| SKCM | Macrophage | ENSG00000177757 | FAM87B | 0.000141 | 0.1748381 | ENSG00000254321 | RP11-495O10.1 | 0.0023554 | -0.1401156 |
| SKCM | Macrophage | ENSG00000179082 | C9orf106 | 1.91E-14 | 0.3436377 | ENSG00000254337 | RP11-865I6.2 | 3.19E-06 | 0.213178 |
| SKCM | Macrophage | ENSG00000180712 | RP11-290F5.2 | 4.99E-06 | 0.2090264 | ENSG00000254438 | RP11-231N3.1 | 0.0020237 | -0.1421863 |
| SKCM | Macrophage | ENSG00000181908 | AP003774.4 | 8.90E-22 | 0.4229518 | ENSG00000254605 | RP11-626H12.2 | 9.92E-06 | 0.2024741 |
| SKCM | Macrophage | ENSG00000183674 | LINC00518 | 2.33E-07 | -0.2360267 | ENSG00000254645 | RP11-396O20.2 | 3.53E-05 | -0.1897585 |
| SKCM | Macrophage | ENSG00000186594 | MIR22HG | 1.62E-06 | 0.2193338 | ENSG00000254802 | AC022182.3 | 1.07E-25 | 0.457986 |
| SKCM | Macrophage | ENSG00000187185 | CTD-2600O9.1 | 0.003274 | -0.1355246 | ENSG00000254872 | RP13-870H17.3 | 0.0002121 | 0.170246 |
| SKCM | Macrophage | ENSG00000197445 | C16orf47 | 0.000411 | 0.1624779 | ENSG00000254973 | RP11-429J17.7 | 4.58E-05 | -0.1870525 |
| SKCM | Macrophage | ENSG00000198547 | C20orf203 | 0.024977 | 0.1035126 | ENSG00000255366 | RP11-1134I14.8 | 0.0002075 | 0.1704954 |
| SKCM | Macrophage | ENSG00000203386 | LINC01317 | 2.39E-07 | -0.2358045 | ENSG00000255375 | RP1-65P5.5 | 2.31E-05 | -0.1941169 |
| SKCM | Macrophage | ENSG00000203684 | IBA57-AS1 | 0.005833 | -0.1271298 | ENSG00000255760 | RP11-428G5.5 | 7.08E-20 | 0.4043646 |
| SKCM | Macrophage | ENSG00000203709 | C1orf132 | 1.95E-08 | 0.255706 | ENSG00000255864 | RP11-444D3.1 | 0.0045942 | -0.1306579 |
| SKCM | Macrophage | ENSG00000204365 | C10orf126 | 0.037298 | -0.09619383 | ENSG00000255933 | RP11-495K9.5 | 5.11E-09 | -0.2656726 |
| SKCM | Macrophage | ENSG00000204792 | LINC01291 | 0.001277 | -0.1483062 | ENSG00000256084 | RP11-134N1.2 | 3.25E-08 | 0.2517894 |
| SKCM | Macrophage | ENSG00000204904 | LINC01545 | 0.017678 | -0.1095053 | ENSG00000256128 | LINC00944 | 3.42E-09 | 0.2685891 |
| SKCM | Macrophage | ENSG00000204929 | AC074391.1 | 0.035106 | 0.09732678 | ENSG00000256443 | RP11-794G24.1 | 0.041974 | -0.09395405 |
| SKCM | Macrophage | ENSG00000205444 | RP4-529N6.1 | 5.58E-07 | -0.2286549 | ENSG00000256540 | RP11-598F7.6 | 5.72E-07 | 0.2284538 |
| SKCM | Macrophage | ENSG00000205562 | RP11-497E19.1 | 1.91E-06 | 0.2178468 | ENSG00000256560 | LINC01486 | 1.23E-10 | -0.2914507 |
| SKCM | Macrophage | ENSG00000205611 | LINC01597 | 0.031052 | 0.09959176 | ENSG00000257114 | RP11-25I15.3 | 0.00434 | 0.131487 |
| SKCM | Macrophage | ENSG00000205622 | AF064858.6 | 1.94E-05 | 0.195834 | ENSG00000257327 | RP11-650K20.3 | 0.0018671 | -0.1432746 |
| SKCM | Macrophage | ENSG00000205786 | LINC01531 | 3.73E-10 | -0.2840649 | ENSG00000257660 | RP11-579D7.4 | 0.0001095 | -0.1776815 |
| SKCM | Macrophage | ENSG00000212743 | RP11-563J2.3 | 0.001785 | 0.1438792 | ENSG00000257894 | RP1-78O14.1 | 6.02E-07 | 0.2280159 |
| SKCM | Macrophage | ENSG00000212766 | EWSAT1 | 0.02719 | -0.1019977 | ENSG00000257924 | RP11-493L12.5 | 1.44E-19 | 0.4012344 |
| SKCM | Macrophage | ENSG00000213373 | LINC00671 | 0.008545 | 0.121306 | ENSG00000258066 | RP11-781A6.1 | 0.0016565 | -0.1448779 |
| SKCM | Macrophage | ENSG00000213888 | LINC01521 | 0.010754 | -0.1176793 | ENSG00000258082 | RP11-443B7.3 | 6.03E-18 | 0.3842124 |
| SKCM | Macrophage | ENSG00000214381 | LINC00488 | 0.007671 | -0.1229742 | ENSG00000258181 | RP11-493L12.4 | 2.99E-22 | 0.4274119 |
| SKCM | Macrophage | ENSG00000214546 | AC087491.2 | 0.021877 | -0.1058427 | ENSG00000258183 | RP11-753N8.1 | 0.016989 | 0.1101772 |
| SKCM | Macrophage | ENSG00000214548 | MEG3 | 7.63E-07 | 0.2259638 | ENSG00000258203 | RP1-228P16.3 | 0.0182647 | -0.1089504 |
| SKCM | Macrophage | ENSG00000214650 | RP11-83B20.1 | 0.009633 | -0.1194267 | ENSG00000258479 | LINC00640 | 0.0260412 | 0.1027705 |
| SKCM | Macrophage | ENSG00000214894 | LINC00243 | 3.87E-05 | 0.1888184 | ENSG00000258498 | DIO3OS | 2.36E-11 | 0.3021058 |
| SKCM | Macrophage | ENSG00000215244 | RP11-563J2.2 | 1.85E-06 | 0.2181293 | ENSG00000258561 | RP11-72M17.1 | 6.93E-06 | -0.2059233 |
| SKCM | Macrophage | ENSG00000215374 | FAM66B | 0.002362 | 0.1400774 | ENSG00000258593 | CTD-3051D23.4 | 0.0020854 | -0.1417791 |
| SKCM | Macrophage | ENSG00000215386 | MIR99AHG | 0.005941 | 0.1268554 | ENSG00000258702 | RP11-433J8.1 | 1.63E-05 | 0.1975709 |
| SKCM | Macrophage | ENSG00000215859 | RP6-74O6.2 | 0.01037 | -0.1182591 | ENSG00000258733 | CTD-2341M24.1 | 2.09E-07 | 0.2368999 |
| SKCM | Macrophage | ENSG00000215866 | LINC01356 | 0.020483 | -0.1069853 | ENSG00000258777 | HIF1A-AS1 | 0.0486783 | 0.09108449 |
| SKCM | Macrophage | ENSG00000219926 | RP11-394A14.2 | 0.034885 | -0.09744478 | ENSG00000258791 | LINC00520 | 5.01E-05 | -0.1860991 |
| SKCM | Macrophage | ENSG00000222005 | LINC01118 | 0.007608 | 0.1231011 | ENSG00000258811 | CTD-3051D23.1 | 0.0236582 | -0.1044715 |
| SKCM | Macrophage | ENSG00000222022 | AC112721.1 | 1.98E-06 | 0.2175373 | ENSG00000258867 | LINC01146 | 7.13E-44 | 0.582038 |
| SKCM | Macrophage | ENSG00000222032 | AC112721.2 | 6.18E-09 | 0.2642805 | ENSG00000258884 | CTD-3035D6.2 | 0.0351936 | -0.09728058 |
| SKCM | Macrophage | ENSG00000223403 | MEG9 | 0.002798 | 0.1377332 | ENSG00000258942 | RP11-255G12.2 | 0.0206927 | 0.1068088 |
| SKCM | Macrophage | ENSG00000223414 | LINC00473 | 2.48E-14 | -0.3422239 | ENSG00000259004 | RP11-8L8.2 | 6.03E-51 | 0.6189928 |
| SKCM | Macrophage | ENSG00000223704 | LINC01422 | 1.16E-06 | 0.2223122 | ENSG00000259054 | AE000662.93 | 0.0053177 | -0.1285066 |
| SKCM | Macrophage | ENSG00000223714 | RP5-1172N10.2 | 0.007025 | -0.1243223 | ENSG00000259380 | RP11-346D14.1 | 0.0489652 | -0.0909693 |
| SKCM | Macrophage | ENSG00000223749 | MIR503HG | 0.000836 | 0.1537462 | ENSG00000259527 | LINC00052 | 5.88E-05 | -0.1844146 |
| SKCM | Macrophage | ENSG00000223764 | RP11-54O7.3 | 2.61E-08 | 0.2534606 | ENSG00000259584 | RP11-521C20.2 | 0.0029202 | -0.1371356 |
| SKCM | Macrophage | ENSG00000223979 | SMCR2 | 0.017381 | -0.1097922 | ENSG00000259687 | LINC01220 | 0.0026984 | -0.1382374 |
| SKCM | Macrophage | ENSG00000224164 | RP3-369A17.4 | 6.74E-12 | 0.3099031 | ENSG00000259711 | CTD-3032H12.2 | 2.97E-09 | -0.2696048 |
| SKCM | Macrophage | ENSG00000224209 | LINC00466 | 0.000154 | -0.1738569 | ENSG00000259828 | RP11-63E9.1 | 4.87E-08 | -0.2486273 |
| SKCM | Macrophage | ENSG00000224259 | LINC01133 | 0.004979 | 0.1294793 | ENSG00000260025 | RP11-490M8.1 | 0.0132214 | 0.1143415 |
| SKCM | Macrophage | ENSG00000224330 | AC005019.3 | 1.54E-07 | -0.2394037 | ENSG00000260052 | CTC-527H23.3 | 2.96E-06 | -0.2138812 |
| SKCM | Macrophage | ENSG00000224511 | LINC00365 | 0.003814 | -0.1333532 | ENSG00000260086 | RP11-42I10.1 | 2.35E-08 | -0.2542873 |
| SKCM | Macrophage | ENSG00000224592 | RP5-884C9.2 | 0.004738 | -0.1302069 | ENSG00000260136 | CTD-2270L9.4 | 3.03E-07 | -0.2338411 |
| SKCM | Macrophage | ENSG00000224652 | LINC00885 | 6.64E-05 | 0.1831167 | ENSG00000260209 | RP11-680F20.10 | 0.0008084 | -0.1541705 |
| SKCM | Macrophage | ENSG00000224790 | AP000704.5 | 0.000276 | -0.1672085 | ENSG00000260219 | RP11-347C12.10 | 0.0003277 | -0.1651812 |
| SKCM | Macrophage | ENSG00000224933 | LINC01034 | 0.009608 | -0.1194674 | ENSG00000260331 | RP11-111J6.2 | 0.0355451 | 0.09709533 |
| SKCM | Macrophage | ENSG00000225007 | AC000067.1 | 0.038407 | 0.09564183 | ENSG00000260418 | RP3-406A7.7 | 0.0229025 | -0.1050419 |
| SKCM | Macrophage | ENSG00000225077 | LINC00337 | 0.033038 | -0.09845252 | ENSG00000260578 | CTD-2541J13.1 | 3.44E-10 | 0.2846143 |
| SKCM | Macrophage | ENSG00000225096 | XXbac-BPG55C20.7 | 1.22E-07 | -0.2413347 | ENSG00000260645 | RP11-250B2.5 | 0.0061678 | 0.1262928 |
| SKCM | Macrophage | ENSG00000225173 | XXbac-BPG308K3.5 | 0.024836 | -0.1036133 | ENSG00000260658 | RP11-368L12.1 | 0.0014209 | -0.14691 |
| SKCM | Macrophage | ENSG00000225206 | MIR137HG | 0.001531 | 0.1459281 | ENSG00000260743 | RP11-255C15.3 | 0.0383318 | 0.09567904 |
| SKCM | Macrophage | ENSG00000225383 | SFTA1P | 9.48E-07 | 0.2240762 | ENSG00000260876 | LINC01229 | 1.45E-07 | 0.239909 |
| SKCM | Macrophage | ENSG00000225535 | LINC01393 | 0.012227 | 0.1156147 | ENSG00000260884 | AC009120.5 | 0.0153053 | 0.1119261 |
| SKCM | Macrophage | ENSG00000225637 | AP001046.6 | 0.01943 | -0.1078931 | ENSG00000260911 | RP11-196G11.2 | 3.15E-05 | 0.1909254 |
| SKCM | Macrophage | ENSG00000225647 | AC005487.2 | 0.002757 | -0.1379388 | ENSG00000260971 | RP11-504A18.1 | 2.45E-07 | 0.2356002 |
| SKCM | Macrophage | ENSG00000225684 | FAM225B | 0.001007 | 0.1513742 | ENSG00000260977 | RP11-333I13.1 | 0.0002086 | 0.1704383 |
| SKCM | Macrophage | ENSG00000225768 | RP11-127O4.3 | 8.59E-05 | -0.180338 | ENSG00000260979 | RP11-77H9.8 | 0.0003512 | 0.1643602 |
| SKCM | Macrophage | ENSG00000225775 | RP11-354I10.1 | 1.43E-05 | -0.1988576 | ENSG00000261000 | RP11-534L20.5 | 1.40E-06 | 0.2206087 |
| SKCM | Macrophage | ENSG00000226004 | RP11-10J5.1 | 4.61E-25 | 0.4525908 | ENSG00000261020 | RP11-744K17.1 | 0.0103974 | -0.1182175 |
| SKCM | Macrophage | ENSG00000226194 | RP1-137D17.1 | 0.003315 | 0.1353524 | ENSG00000261039 | RP11-417E7.2 | 2.87E-05 | 0.191897 |
| SKCM | Macrophage | ENSG00000226281 | RP1-80N2.2 | 0.001753 | 0.1441181 | ENSG00000261064 | RP11-1000B6.3 | 0.0261673 | 0.1026843 |
| SKCM | Macrophage | ENSG00000226363 | HAGLROS | 0.002413 | -0.1397815 | ENSG00000261097 | LINC00563 | 5.91E-07 | 0.2281611 |
| SKCM | Macrophage | ENSG00000226530 | RP11-348F1.2 | 0.00221 | 0.1409907 | ENSG00000261218 | RP11-960L18.1 | 4.97E-20 | 0.4059124 |
| SKCM | Macrophage | ENSG00000226708 | AC073409.1 | 0.004636 | -0.1305266 | ENSG00000261364 | RP11-59E19.4 | 0.003939 | -0.1328885 |
| SKCM | Macrophage | ENSG00000226751 | AF127936.5 | 4.81E-42 | 0.5716497 | ENSG00000261618 | RP11-79H23.3 | 2.69E-11 | 0.3012874 |
| SKCM | Macrophage | ENSG00000226791 | AC109826.1 | 7.56E-09 | 0.2628027 | ENSG00000261671 | RP11-573G6.6 | 1.55E-05 | 0.1981206 |
| SKCM | Macrophage | ENSG00000226798 | RP11-289F5.1 | 0.00081 | -0.1541493 | ENSG00000261754 | CTC-523E23.1 | 6.74E-17 | 0.3726521 |
| SKCM | Macrophage | ENSG00000226808 | LINC00840 | 3.38E-14 | 0.3405307 | ENSG00000261795 | RP11-90P13.1 | 6.43E-12 | 0.3101919 |
| SKCM | Macrophage | ENSG00000226816 | AC005082.12 | 5.60E-30 | 0.4921381 | ENSG00000261888 | AC144831.1 | 0.000395 | 0.1629578 |
| SKCM | Macrophage | ENSG00000226853 | AC010894.3 | 3.39E-05 | -0.190182 | ENSG00000261996 | CTC-281F24.1 | 6.13E-05 | 0.183978 |
| SKCM | Macrophage | ENSG00000227374 | RP11-109A6.3 | 0.023268 | 0.1047638 | ENSG00000262223 | RP11-1055B8.3 | 2.31E-10 | -0.2872953 |
| SKCM | Macrophage | ENSG00000227482 | RP11-18B16.2 | 0.027257 | 0.1019535 | ENSG00000262370 | RP11-473M20.9 | 1.54E-33 | 0.5178762 |
| SKCM | Macrophage | ENSG00000227486 | RP13-188A5.1 | 0.000493 | 0.1602965 | ENSG00000262714 | RP11-44F14.8 | 0.0002283 | 0.1694004 |
| SKCM | Macrophage | ENSG00000227531 | RP11-202G18.1 | 1.29E-31 | 0.5042467 | ENSG00000262873 | CTD-2561B21.11 | 0.001484 | -0.1463363 |
| SKCM | Macrophage | ENSG00000227676 | LINC01068 | 0.003001 | 0.1367517 | ENSG00000263033 | RP11-396B14.2 | 0.0015501 | 0.1457601 |
| SKCM | Macrophage | ENSG00000228022 | HCG20 | 0.000717 | -0.1556739 | ENSG00000263105 | RP11-95P2.3 | 9.64E-05 | -0.1790862 |
| SKCM | Macrophage | ENSG00000228033 | AC010967.2 | 1.81E-07 | -0.2381151 | ENSG00000263154 | RP11-1055B8.2 | 3.10E-12 | -0.3146074 |
| SKCM | Macrophage | ENSG00000228065 | LINC01515 | 0.019197 | 0.1080999 | ENSG00000263207 | RP11-26L20.4 | 1.70E-07 | -0.2386064 |
| SKCM | Macrophage | ENSG00000228262 | LINC01320 | 6.66E-06 | -0.2062952 | ENSG00000263321 | RP11-388C12.5 | 0.0227284 | -0.1051757 |
| SKCM | Macrophage | ENSG00000228459 | LINC01546 | 3.77E-15 | 0.3522802 | ENSG00000263655 | RP11-25L3.3 | 0.0005511 | 0.1589283 |
| SKCM | Macrophage | ENSG00000228478 | RP1-290I10.3 | 2.56E-09 | -0.2706583 | ENSG00000263812 | LINC00908 | 1.27E-05 | 0.2000368 |
| SKCM | Macrophage | ENSG00000228549 | RP11-108M9.3 | 0.005652 | 0.1276006 | ENSG00000263821 | RP11-527H14.1 | 0.0018677 | -0.1432704 |
| SKCM | Macrophage | ENSG00000228590 | AC007381.3 | 7.05E-09 | 0.2633191 | ENSG00000264301 | LINC01444 | 0.0223516 | -0.1054681 |
| SKCM | Macrophage | ENSG00000228734 | RP11-335E6.3 | 0.006316 | -0.1259363 | ENSG00000264707 | L3MBTL4-AS1 | 5.74E-11 | 0.2964324 |
| SKCM | Macrophage | ENSG00000228741 | SPATA13 | 0.02262 | 0.1052597 | ENSG00000265975 | CTB-41I6.2 | 1.09E-37 | 0.5453344 |
| SKCM | Macrophage | ENSG00000228742 | RP5-884M6.1 | 0.000193 | 0.1713149 | ENSG00000266010 | GATA6-AS1 | 0.0005752 | 0.1584039 |
| SKCM | Macrophage | ENSG00000228784 | LINC00954 | 7.30E-11 | 0.2948784 | ENSG00000266256 | LINC00683 | 0.0024574 | 0.1395324 |
| SKCM | Macrophage | ENSG00000228971 | RP11-286B14.1 | 0.021614 | -0.1060529 | ENSG00000266389 | CTB-41I6.1 | 3.24E-39 | 0.5548242 |
| SKCM | Macrophage | ENSG00000229108 | MEOX2-AS1 | 1.44E-09 | 0.2747665 | ENSG00000266554 | LINC01443 | 0.000724 | -0.1555529 |
| SKCM | Macrophage | ENSG00000229116 | RP11-20J15.3 | 1.41E-15 | 0.3573986 | ENSG00000266588 | RP1-56K13.5 | 1.46E-08 | 0.2578984 |
| SKCM | Macrophage | ENSG00000229140 | CCDC26 | 0.011699 | -0.1163284 | ENSG00000266835 | GAPLINC | 0.0060137 | -0.126673 |
| SKCM | Macrophage | ENSG00000229195 | AC009495.4 | 1.38E-08 | -0.2583408 | ENSG00000266977 | CTC-459F4.5 | 4.40E-05 | -0.1874791 |
| SKCM | Macrophage | ENSG00000229246 | LINC00377 | 7.87E-05 | 0.1812945 | ENSG00000267107 | PCAT19 | 2.62E-11 | 0.3014493 |
| SKCM | Macrophage | ENSG00000229588 | RP11-479J7.2 | 5.91E-06 | 0.2074295 | ENSG00000267114 | CTB-129P6.11 | 0.0196158 | -0.1077301 |
| SKCM | Macrophage | ENSG00000229613 | LINC01501 | 1.03E-14 | 0.3469654 | ENSG00000267325 | LINC01415 | 9.22E-14 | 0.3349892 |
| SKCM | Macrophage | ENSG00000229656 | RP11-462L8.1 | 0.001349 | 0.1475904 | ENSG00000267374 | RP11-244M2.1 | 0.0078655 | 0.1225893 |
| SKCM | Macrophage | ENSG00000229660 | RP5-1142J19.1 | 0.049886 | -0.09060322 | ENSG00000267466 | RP11-13K12.5 | 3.12E-06 | 0.2133769 |
| SKCM | Macrophage | ENSG00000229671 | LINC01150 | 7.29E-44 | 0.5819831 | ENSG00000267496 | FAM215A | 1.04E-05 | 0.2020191 |
| SKCM | Macrophage | ENSG00000230107 | CTA-126B4.7 | 1.13E-10 | 0.2920412 | ENSG00000267506 | RP11-13K12.1 | 3.19E-09 | 0.2690796 |
| SKCM | Macrophage | ENSG00000230138 | RP11-117D22.2 | 6.38E-17 | 0.3729225 | ENSG00000267528 | AC011524.1 | 1.21E-08 | -0.2593106 |
| SKCM | Macrophage | ENSG00000230366 | DSCR9 | 0.044994 | -0.09261699 | ENSG00000267583 | RP11-322E11.5 | 1.72E-07 | 0.2385382 |
| SKCM | Macrophage | ENSG00000230381 | RP4-655J12.5 | 7.42E-05 | -0.1819187 | ENSG00000267612 | CTD-3116E22.7 | 3.52E-05 | 0.1897783 |
| SKCM | Macrophage | ENSG00000230390 | LINC01048 | 1.38E-05 | 0.1992587 | ENSG00000267649 | CTD-2587H24.10 | 0.0040586 | -0.1324575 |
| SKCM | Macrophage | ENSG00000230499 | AC108463.1 | 4.60E-07 | 0.2303053 | ENSG00000267658 | RP11-358B23.1 | 0.0060565 | -0.1265665 |
| SKCM | Macrophage | ENSG00000230515 | AC092580.3 | 0.035531 | 0.09710288 | ENSG00000267683 | AC008991.1 | 1.05E-11 | -0.3071486 |
| SKCM | Macrophage | ENSG00000230526 | RP11-472G21.2 | 0.030888 | 0.09968872 | ENSG00000267731 | RP11-147L13.8 | 3.21E-10 | 0.2850858 |
| SKCM | Macrophage | ENSG00000230537 | RP11-305L7.1 | 1.30E-11 | 0.3058306 | ENSG00000267737 | AC061992.2 | 3.19E-08 | 0.2519232 |
| SKCM | Macrophage | ENSG00000230555 | RP11-517P14.2 | 0.001759 | 0.1440747 | ENSG00000268027 | AC006129.2 | 7.53E-28 | 0.4756429 |
| SKCM | Macrophage | ENSG00000230587 | AC093609.1 | 8.08E-11 | 0.2942166 | ENSG00000268087 | CTC-429P9.2 | 0.0120085 | 0.1159067 |
| SKCM | Macrophage | ENSG00000230836 | LINC01293 | 5.49E-05 | -0.1851405 | ENSG00000268266 | AC003005.2 | 0.0005853 | -0.1581883 |
| SKCM | Macrophage | ENSG00000230943 | RP11-367G18.1 | 3.47E-09 | 0.2684793 | ENSG00000268307 | CTD-2619J13.13 | 0.0038999 | -0.1330321 |
| SKCM | Macrophage | ENSG00000231246 | RP5-965F6.2 | 9.76E-11 | 0.2929872 | ENSG00000268333 | RP4-806M20.3 | 0.0353043 | -0.09722208 |
| SKCM | Macrophage | ENSG00000231367 | AC016995.3 | 0.041507 | -0.094168 | ENSG00000268658 | LINC00664 | 0.0047993 | 0.1300187 |
| SKCM | Macrophage | ENSG00000231453 | LINC01305 | 0.037984 | 0.09585117 | ENSG00000268707 | RP11-247A12.7 | 0.0009295 | 0.1524027 |
| SKCM | Macrophage | ENSG00000231528 | FAM225A | 0.001881 | 0.1431766 | ENSG00000268734 | CTB-61M7.2 | 5.36E-17 | 0.3737674 |
| SKCM | Macrophage | ENSG00000231680 | AP003774.6 | 7.06E-23 | 0.4332101 | ENSG00000268945 | CTD-2192J16.26 | 0.000459 | -0.1611533 |
| SKCM | Macrophage | ENSG00000231690 | LINC00574 | 7.47E-05 | 0.1818467 | ENSG00000269086 | CTC-523E23.5 | 4.25E-11 | 0.2983715 |
| SKCM | Macrophage | ENSG00000231768 | LINC01354 | 1.33E-07 | 0.2406377 | ENSG00000269246 | CTC-246B18.10 | 0.0001025 | -0.1784143 |
| SKCM | Macrophage | ENSG00000231811 | RP3-527G5.1 | 0.000915 | -0.1525961 | ENSG00000269902 | RP6-99M1.3 | 0.0079553 | 0.122414 |
| SKCM | Macrophage | ENSG00000232028 | AC007391.2 | 3.60E-05 | -0.1895654 | ENSG00000269927 | RP6-91H8.3 | 0.0378123 | 0.09593627 |
| SKCM | Macrophage | ENSG00000232053 | AC009784.3 | 0.005558 | 0.1278506 | ENSG00000269930 | RP11-932O9.9 | 0.0029222 | 0.1371261 |
| SKCM | Macrophage | ENSG00000232118 | BACH1-AS1 | 0.016411 | 0.1107602 | ENSG00000269967 | RP11-84A19.4 | 0.0005638 | 0.1586488 |
| SKCM | Macrophage | ENSG00000232411 | AC009495.3 | 3.62E-09 | -0.2681812 | ENSG00000269976 | RP11-130L8.2 | 0.0043694 | 0.1313889 |
| SKCM | Macrophage | ENSG00000232412 | RP1-315G1.3 | 0.00063 | -0.1572851 | ENSG00000270069 | MIR222HG | 0.0047314 | 0.1302276 |
| SKCM | Macrophage | ENSG00000232774 | FLJ22447 | 2.52E-05 | 0.1932219 | ENSG00000270090 | RP11-529E10.7 | 0.0017505 | -0.1441406 |
| SKCM | Macrophage | ENSG00000232855 | AF131217.1 | 8.09E-16 | 0.3602471 | ENSG00000270093 | AP000473.8 | 4.46E-05 | 0.1873355 |
| SKCM | Macrophage | ENSG00000232931 | LINC00342 | 0.046627 | 0.09192515 | ENSG00000270210 | RP11-373D23.3 | 0.0003132 | 0.1657139 |
| SKCM | Macrophage | ENSG00000233056 | ERVH48-1 | 0.005591 | 0.1277611 | ENSG00000270562 | RP11-154H23.3 | 2.25E-12 | 0.3165475 |
| SKCM | Macrophage | ENSG00000233081 | RP11-440G5.2 | 0.031056 | 0.09958947 | ENSG00000270607 | RP11-359E10.1 | 0.0342821 | -0.09776849 |
| SKCM | Macrophage | ENSG00000233379 | RP11-318G21.4 | 0.012906 | -0.1147356 | ENSG00000270659 | RP11-105N14.1 | 0.0074019 | 0.1235234 |
| SKCM | Macrophage | ENSG00000233593 | RP4-665J23.1 | 4.32E-09 | -0.2668931 | ENSG00000270972 | RP11-326C3.15 | 0.0002069 | 0.1705332 |
| SKCM | Macrophage | ENSG00000233760 | AC004947.2 | 0.014485 | 0.1128405 | ENSG00000270996 | RP11-342K6.4 | 0.006103 | -0.1264516 |
| SKCM | Macrophage | ENSG00000233858 | AC026904.1 | 5.88E-06 | 0.2074802 | ENSG00000271086 | NAMA | 0.0009243 | -0.1524734 |
| SKCM | Macrophage | ENSG00000233901 | LINC01503 | 9.15E-11 | 0.2934089 | ENSG00000271109 | CTC-523E23.11 | 3.16E-14 | 0.3409095 |
| SKCM | Macrophage | ENSG00000233967 | RP11-250B2.3 | 0.022404 | 0.1054272 | ENSG00000271192 | RP4-555D20.3 | 0.0156278 | -0.1115785 |
| SKCM | Macrophage | ENSG00000233975 | RP11-288L9.1 | 0.0367 | 0.09649729 | ENSG00000271384 | RP11-435O5.7 | 0.0075618 | -0.1231956 |
| SKCM | Macrophage | ENSG00000233987 | AC106706.1 | 0.000131 | -0.1757351 | ENSG00000271815 | CTD-2235C13.3 | 0.002488 | -0.139362 |
| SKCM | Macrophage | ENSG00000234076 | TPRG1-AS1 | 2.63E-10 | 0.2864291 | ENSG00000271840 | RP1-224A6.9 | 0.0035004 | 0.1345778 |
| SKCM | Macrophage | ENSG00000234155 | RP11-30P6.6 | 0.012377 | 0.1154174 | ENSG00000271892 | CTD-2228A4.1 | 4.65E-05 | -0.1868971 |
| SKCM | Macrophage | ENSG00000234215 | RP5-942I16.1 | 0.001182 | -0.1493126 | ENSG00000271926 | CTD-2376I4.1 | 0.0002721 | 0.167364 |
| SKCM | Macrophage | ENSG00000234323 | LINC01505 | 0.00335 | -0.1352023 | ENSG00000271930 | RP11-44N12.5 | 8.42E-06 | 0.2040507 |
| SKCM | Macrophage | ENSG00000234506 | LINC01506 | 3.41E-20 | 0.4075502 | ENSG00000271983 | RP11-28H5.2 | 0.0476682 | -0.09149473 |
| SKCM | Macrophage | ENSG00000234690 | AC073283.4 | 0.006511 | 0.1254762 | ENSG00000271991 | RP11-79O8.1 | 0.0034558 | -0.1347603 |
| SKCM | Macrophage | ENSG00000234902 | AC007879.2 | 8.44E-08 | 0.2442761 | ENSG00000271992 | RP11-42O15.3 | 0.0005799 | -0.158302 |
| SKCM | Macrophage | ENSG00000235172 | LINC01366 | 6.64E-08 | -0.2461842 | ENSG00000272002 | RP11-557L19.1 | 0.009362 | -0.1198766 |
| SKCM | Macrophage | ENSG00000235271 | LINC01422 | 0.014299 | 0.113054 | ENSG00000272023 | CTC-350I8.1 | 0.0020459 | -0.1420387 |
| SKCM | Macrophage | ENSG00000235304 | LINC01281 | 2.20E-18 | 0.3889006 | ENSG00000272049 | RP11-480D4.6 | 0.0181565 | -0.1090515 |
| SKCM | Macrophage | ENSG00000235319 | AC012360.4 | 0.000424 | -0.1621149 | ENSG00000272076 | RP11-11C20.3 | 0.0198772 | 0.1075025 |
| SKCM | Macrophage | ENSG00000235366 | LINC01055 | 1.91E-13 | 0.3308955 | ENSG00000272077 | RP11-348P10.2 | 0.0001117 | 0.1774585 |
| SKCM | Macrophage | ENSG00000235532 | LINC00402 | 2.52E-18 | 0.3882768 | ENSG00000272081 | CTD-2376I4.2 | 9.97E-11 | 0.292844 |
| SKCM | Macrophage | ENSG00000235615 | AJ239322.1 | 0.000132 | 0.1756312 | ENSG00000272108 | AC005754.8 | 0.0049775 | 0.1294831 |
| SKCM | Macrophage | ENSG00000235659 | RP11-374M1.5 | 0.000864 | 0.1533362 | ENSG00000272121 | RP4-555D20.4 | 2.46E-10 | -0.2868702 |
| SKCM | Macrophage | ENSG00000235884 | LINC00941 | 0.005041 | 0.1292963 | ENSG00000272140 | RP11-574K11.29 | 0.0118019 | 0.1161871 |
| SKCM | Macrophage | ENSG00000235888 | AF064858.8 | 2.37E-08 | 0.2542197 | ENSG00000272205 | RP11-277B15.3 | 0.0055497 | -0.1278725 |
| SKCM | Macrophage | ENSG00000235947 | EGOT | 6.26E-09 | 0.2641901 | ENSG00000272255 | CTD-3224K15.3 | 0.0222838 | 0.1055212 |
| SKCM | Macrophage | ENSG00000236013 | RP3-332B22.1 | 0.003355 | -0.1351811 | ENSG00000272264 | RP11-92K15.3 | 0.0434162 | 0.09330577 |
| SKCM | Macrophage | ENSG00000236230 | RP11-400N13.1 | 0.034398 | -0.09770577 | ENSG00000272316 | XXbac-BPGBPG55C20.2 | 0.0129609 | 0.1146666 |
| SKCM | Macrophage | ENSG00000236384 | LINC00479 | 0.00031 | -0.1658311 | ENSG00000272334 | RP11-129K12.1 | 0.0125187 | 0.115232 |
| SKCM | Macrophage | ENSG00000236385 | RP11-114M1.2 | 0.003012 | -0.1367026 | ENSG00000272379 | RP1-257A7.5 | 3.63E-05 | -0.1894728 |
| SKCM | Macrophage | ENSG00000236393 | RP11-320G24.1 | 0.001336 | 0.1477195 | ENSG00000272416 | CTD-2081C10.7 | 5.97E-09 | 0.2645317 |
| SKCM | Macrophage | ENSG00000236544 | AC008060.8 | 0.005276 | -0.1286242 | ENSG00000272425 | RP11-363E6.4 | 0.0032516 | -0.1356238 |
| SKCM | Macrophage | ENSG00000236819 | LINC01563 | 0.022548 | -0.1053148 | ENSG00000272430 | RP11-38L15.8 | 0.0017661 | -0.1440215 |
| SKCM | Macrophage | ENSG00000236985 | RP5-1195D24.1 | 0.001021 | 0.151206 | ENSG00000272473 | AC006273.4 | 0.0457757 | 0.09228332 |
| SKCM | Macrophage | ENSG00000237352 | LINC01358 | 6.23E-17 | 0.373041 | ENSG00000272512 | RP11-54O7.17 | 0.0061602 | -0.1263115 |
| SKCM | Macrophage | ENSG00000237372 | UNQ6494 | 6.00E-20 | 0.4050896 | ENSG00000272524 | RP11-254F7.4 | 0.048234 | -0.09126403 |
| SKCM | Macrophage | ENSG00000237614 | AC073257.2 | 0.000576 | 0.1583947 | ENSG00000272583 | RP11-344P13.6 | 4.61E-05 | 0.1869853 |
| SKCM | Macrophage | ENSG00000237721 | AF064858.11 | 9.26E-06 | 0.2031411 | ENSG00000272662 | RP11-190C22.8 | 0.00022 | -0.1698229 |
| SKCM | Macrophage | ENSG00000237751 | LINC01143 | 1.17E-05 | -0.2008564 | ENSG00000272682 | AC004471.10 | 0.0068493 | -0.124708 |
| SKCM | Macrophage | ENSG00000237790 | LINC01318 | 5.28E-06 | -0.2084966 | ENSG00000272695 | GAS6-AS2 | 1.21E-07 | 0.2414004 |
| SKCM | Macrophage | ENSG00000237879 | LINC00398 | 0.031927 | 0.09908264 | ENSG00000272711 | RP11-259N19.1 | 7.55E-11 | -0.2946631 |
| SKCM | Macrophage | ENSG00000239482 | RP11-90K6.1 | 0.013151 | -0.1144288 | ENSG00000272763 | RP11-357H14.17 | 0.0009356 | 0.1523192 |
| SKCM | Macrophage | ENSG00000240050 | RP1-93H18.1 | 5.45E-12 | 0.3111937 | ENSG00000272798 | CTA-390C10.9 | 6.95E-05 | -0.1826337 |
| SKCM | Macrophage | ENSG00000240219 | RP11-430C7.5 | 2.52E-08 | 0.2537407 | ENSG00000272824 | RP6-74O6.6 | 0.0265934 | 0.1023956 |
| SKCM | Macrophage | ENSG00000240350 | AC017002.1 | 3.52E-15 | 0.3526409 | ENSG00000272855 | RP5-1102E8.3 | 0.035477 | -0.09713106 |
| SKCM | Macrophage | ENSG00000241657 | TRBV11-2 | 8.31E-25 | 0.4503886 | ENSG00000272871 | RP11-408A13.4 | 0.0132724 | 0.1142785 |
| SKCM | Macrophage | ENSG00000241912 | RP11-292E2.2 | 0.016265 | 0.1109098 | ENSG00000272908 | RP11-121A8.1 | 1.29E-53 | 0.6317622 |
| SKCM | Macrophage | ENSG00000242147 | RP13-463N16.6 | 0.004396 | 0.1312997 | ENSG00000273004 | GS1-279B7.2 | 0.005685 | -0.1275138 |
| SKCM | Macrophage | ENSG00000242258 | LINC00996 | 1.24E-28 | 0.4818306 | ENSG00000273059 | XXyac-YX155B6.7 | 0.0005064 | -0.1599607 |
| SKCM | Macrophage | ENSG00000244342 | LINC00698 | 0.037596 | -0.09604429 | ENSG00000273102 | AP000569.9 | 0.000923 | 0.1524913 |
| SKCM | Macrophage | ENSG00000245164 | LINC00861 | 2.11E-26 | 0.4638825 | ENSG00000273139 | XXbac-B444P24.14 | 5.30E-05 | -0.1855216 |
| SKCM | Macrophage | ENSG00000246223 | LINC01550 | 4.36E-17 | 0.3747686 | ENSG00000273162 | RP11-108L7.15 | 0.0210638 | -0.106501 |
| SKCM | Macrophage | ENSG00000246363 | RP11-13A1.1 | 1.79E-07 | 0.2381969 | ENSG00000273214 | RP5-1039K5.18 | 0.0041082 | -0.1322822 |
| SKCM | Macrophage | ENSG00000246375 | RP11-10L7.1 | 0.019154 | 0.1081386 | ENSG00000273272 | CTA-384D8.34 | 2.48E-13 | 0.3294127 |
| SKCM | Macrophage | ENSG00000246430 | LINC00968 | 3.35E-23 | 0.4361603 | ENSG00000273295 | AP000350.5 | 0.0171944 | 0.1099743 |
| SKCM | Macrophage | ENSG00000246898 | LINC00920 | 0.001105 | -0.1501838 | ENSG00000273321 | RP11-621L6.3 | 2.51E-19 | -0.3987612 |
| SKCM | Macrophage | ENSG00000247095 | MIR210HG | 0.000159 | -0.1735344 | ENSG00000273341 | RP5-899E9.1 | 7.35E-33 | 0.5131316 |
| SKCM | Macrophage | ENSG00000247970 | RP11-543C4.1 | 0.037956 | -0.09586482 | ENSG00000273415 | RP11-702B10.2 | 1.64E-06 | 0.2192357 |
| SKCM | Macrophage | ENSG00000247982 | LINC00926 | 1.74E-07 | 0.2384096 | ENSG00000273576 | RP11-390P24.1 | 0.0070439 | 0.1242815 |
| SKCM | Macrophage | ENSG00000248079 | DPH6-AS1 | 0.028164 | 0.1013649 | ENSG00000273669 | RP11-405M12.4 | 1.04E-20 | 0.4126409 |
| SKCM | Macrophage | ENSG00000248479 | RP11-807H7.2 | 0.007117 | -0.1241243 | ENSG00000273771 | RP11-236L14.2 | 1.60E-06 | 0.2194288 |
| SKCM | Macrophage | ENSG00000248636 | RP11-768F21.1 | 3.42E-09 | 0.2685808 | ENSG00000273812 | WI2-87327B8.2 | 4.80E-07 | 0.2299495 |
| SKCM | Macrophage | ENSG00000248714 | RP11-1079K10.3 | 0.003531 | 0.134454 | ENSG00000273821 | RP5-963E22.6 | 0.0047487 | -0.1301741 |
| SKCM | Macrophage | ENSG00000249069 | LINC01033 | 0.007034 | 0.1243035 | ENSG00000273958 | RP11-219B17.3 | 0.0234634 | 0.104617 |
| SKCM | Macrophage | ENSG00000249236 | CTD-2227I18.1 | 0.000108 | -0.1778288 | ENSG00000273972 | CTD-2306A12.1 | 2.07E-07 | 0.2369833 |
| SKCM | Macrophage | ENSG00000249388 | RP11-834C11.6 | 0.000136 | 0.1753028 | ENSG00000273980 | RP13-49I15.6 | 0.0365174 | -0.0965908 |
| SKCM | Macrophage | ENSG00000249476 | CTD-2587M2.1 | 0.000131 | 0.1757068 | ENSG00000273998 | RP4-777L9.2 | 0.0244138 | -0.1039168 |
| SKCM | Macrophage | ENSG00000249740 | OSMR-AS1 | 9.77E-05 | 0.1789311 | ENSG00000274292 | RP11-347I19.7 | 0.0357135 | 0.09700709 |
| SKCM | Macrophage | ENSG00000249790 | RP11-20D14.6 | 1.19E-05 | 0.2006887 | ENSG00000274370 | AC144831.3 | 0.0027856 | 0.1377949 |
| SKCM | Macrophage | ENSG00000249816 | LINC00964 | 0.008918 | 0.1206377 | ENSG00000274678 | RP11-2C24.7 | 0.0423443 | 0.0937858 |
| SKCM | Macrophage | ENSG00000250056 | LINC01018 | 0.000174 | 0.1724854 | ENSG00000274695 | RP11-21K12.3 | 0.0012791 | -0.1482872 |
| SKCM | Macrophage | ENSG00000250125 | RP11-707A18.1 | 0.044752 | -0.09272136 | ENSG00000274833 | RP11-1055B8.10 | 1.04E-13 | -0.3343099 |
| SKCM | Macrophage | ENSG00000250334 | LINC00989 | 1.36E-08 | 0.2583992 | ENSG00000275142 | RP5-999L4.2 | 0.0145061 | 0.1128161 |
| SKCM | Macrophage | ENSG00000250400 | LINC00977 | 0.01228 | -0.1155451 | ENSG00000275263 | RP11-1072A3.4 | 0.000294 | -0.1664587 |
| SKCM | Macrophage | ENSG00000250846 | EPHA5-AS1 | 2.68E-10 | -0.2862956 | ENSG00000275557 | RP11-353N4.6 | 0.0221766 | 0.1056054 |
| SKCM | Macrophage | ENSG00000250889 | LINC01336 | 0.009497 | 0.119651 | ENSG00000275703 | U47924.32 | 0.0051796 | 0.1288962 |
| SKCM | Macrophage | ENSG00000251323 | RP11-452H21.4 | 0.033903 | -0.09797478 | ENSG00000275963 | RP11-180M15.6 | 0.0244226 | -0.1039103 |
| SKCM | Macrophage | ENSG00000251381 | LINC00958 | 0.008587 | -0.1212286 | ENSG00000276216 | CH17-373J23.1 | 0.0001853 | 0.1717894 |
| SKCM | Macrophage | ENSG00000251432 | RP11-420A23.1 | 0.000623 | -0.157417 | ENSG00000276399 | FLJ36000 | 0.0005512 | -0.1589257 |
| SKCM | Macrophage | ENSG00000251517 | RP11-109E24.1 | 4.68E-19 | 0.3959694 | ENSG00000276417 | RP11-266K4.13 | 0.0135221 | 0.1139732 |
| SKCM | Macrophage | ENSG00000251615 | RP11-774O3.3 | 7.06E-06 | 0.2057381 | ENSG00000277135 | RP11-327J17.9 | 1.91E-05 | 0.1960003 |
| SKCM | Macrophage | ENSG00000251637 | RP11-119D9.1 | 0.000245 | 0.1685758 | ENSG00000277214 | RP11-70D24.3 | 0.0389007 | 0.0954007 |
| SKCM | Macrophage | ENSG00000253177 | RP11-100L22.1 | 1.55E-05 | 0.1980941 | ENSG00000277235 | RP4-550H1.7 | 0.0295216 | -0.1005127 |
| SKCM | Macrophage | ENSG00000253214 | RP11-1149M10.2 | 0.000163 | 0.1732651 | ENSG00000277453 | CTC-492K19.7 | 0.0136139 | -0.1138621 |
| SKCM | Macrophage | ENSG00000253364 | RP11-731F5.2 | 3.74E-16 | 0.3641595 | ENSG00000278041 | RP5-984P4.6 | 3.75E-12 | -0.3134677 |
| SKCM | Macrophage | ENSG00000253400 | RP11-337A23.6 | 0.001078 | -0.150505 | ENSG00000278231 | RP5-906C1.1 | 0.0113373 | 0.1168338 |
| SKCM | Macrophage | ENSG00000253633 | KB-1980E6.3 | 8.32E-05 | 0.1806891 | ENSG00000278238 | RP11-245D16.4 | 0.0001461 | -0.1744777 |
| SKCM | Macrophage | ENSG00000253701 | AL928768.3 | 2.17E-09 | 0.2718525 | ENSG00000278514 | AC068831.16 | 5.42E-05 | -0.1852759 |
| SKCM | Macrophage | ENSG00000253746 | RP11-527N22.2 | 0.005285 | 0.1285979 | ENSG00000278595 | RP5-1068H6.6 | 0.0212972 | -0.1063099 |
| SKCM | Macrophage | ENSG00000253844 | RP11-546K22.1 | 0.001756 | -0.1440953 | ENSG00000278740 | RP11-147L13.14 | 8.97E-08 | 0.243788 |
