# Supplementary material for: Tumor-infiltrating macrophage associated lncRNA signature in cutaneous melanoma: implications for diagnosis, prognosis, and immunotherapy
Source: Aging (Albany NY). 2024 Mar 13;16(5):4518–40. doi: 10.18632/aging.205606 (PMC10968696; doi:10.18632/aging.205606)
Supplement: Supplementary Table 2 [file aging-16-205606-s003.docx]

Supplementary Table 2. The differentially expressed lncRNAs between tumor and normal groups in TCGA-SKCM&GTEx.

| Smybol | logFC | AveExpr | t | P.Value | adj.P.Val | B | change |
| --- | --- | --- | --- | --- | --- | --- | --- |
| RP11-143A12.3 | 9.284437 | -3.39625 | 57.59831 | 2.22E-267 | 1.55E-264 | 602.2831 | UP |
| BANCR | 12.60543 | -0.88824 | 45.96879 | 2.55E-213 | 5.29E-211 | 477.9247 | UP |
| RP11-127O4.3 | 8.421252 | -3.9544 | 42.13158 | 6.83E-194 | 9.84E-192 | 433.2215 | UP |
| LINC00998 | 2.384869 | 5.053382 | 41.2577 | 2.34E-189 | 3.04E-187 | 422.785 | UP |
| RP4-555D20.4 | 7.547185 | -2.56707 | 40.97499 | 7.00E-188 | 8.80E-186 | 419.3886 | UP |
| CTD-2207A17.1 | 7.748248 | -4.56069 | 38.66185 | 1.20E-175 | 1.31E-173 | 391.2378 | UP |
| RP11-259N19.1 | 3.630541 | 0.795739 | 37.68674 | 2.07E-170 | 2.12E-168 | 379.1825 | UP |
| RP11-308N19.1 | 7.560746 | -3.06999 | 37.04982 | 5.80E-167 | 5.77E-165 | 371.2501 | UP |
| CTC-231O11.1 | 4.710915 | 1.773537 | 36.38799 | 2.32E-163 | 2.20E-161 | 362.96 | UP |
| LINC01212 | 8.052483 | -3.31216 | 33.93717 | 7.54E-150 | 5.73E-148 | 331.8633 | UP |
| BAIAP2-AS1 | 2.097447 | 2.302816 | 33.81508 | 3.61E-149 | 2.71E-147 | 330.2988 | UP |
| AC079767.4 | 8.073604 | -3.52925 | 33.26228 | 4.40E-146 | 3.18E-144 | 323.1978 | UP |
| LINC01234 | 6.980581 | -1.59473 | 33.15028 | 1.86E-145 | 1.30E-143 | 321.7559 | UP |
| GAPLINC | 6.841457 | 0.776618 | 32.62319 | 1.68E-142 | 1.06E-140 | 314.955 | UP |
| AC096559.1 | 7.626102 | -2.45316 | 31.97813 | 7.16E-139 | 4.26E-137 | 306.601 | UP |
| RP11-347C12.10 | 2.040479 | 0.639858 | 31.70127 | 2.61E-137 | 1.51E-135 | 303.0054 | UP |
| MIR4435-2HG | 2.69314 | 5.039823 | 31.49716 | 3.72E-136 | 2.11E-134 | 300.351 | UP |
| LINC00920 | 4.127834 | 0.934951 | 31.2704 | 7.14E-135 | 4.00E-133 | 297.3985 | UP |
| RP11-210M15.2 | 4.8631 | -1.19116 | 31.04504 | 1.35E-133 | 7.50E-132 | 294.4606 | UP |
| CTD-2270L9.4 | 2.534169 | 1.080021 | 30.6949 | 1.31E-131 | 7.02E-130 | 289.8894 | UP |
| RP11-347E10.1 | 8.347432 | -2.03531 | 30.64458 | 2.53E-131 | 1.34E-129 | 289.2316 | UP |
| LINC00467 | 2.300875 | 2.051719 | 30.47971 | 2.19E-130 | 1.13E-128 | 287.0759 | UP |
| LINC00152 | 2.487198 | 4.684147 | 30.44947 | 3.25E-130 | 1.67E-128 | 286.6804 | UP |
| LINC00973 | 6.946676 | -4.86728 | 30.22931 | 5.80E-129 | 2.97E-127 | 283.7986 | UP |
| LINC00518 | 5.378415 | 2.166741 | 30.07585 | 4.34E-128 | 2.15E-126 | 281.7883 | UP |
| RP4-665J23.1 | 2.917539 | 1.333261 | 29.30656 | 1.06E-123 | 4.98E-122 | 271.6914 | UP |
| LINC00589 | 7.806707 | -2.7136 | 29.2204 | 3.29E-123 | 1.51E-121 | 270.5588 | UP |
| RP1-290I10.7 | 6.861108 | -4.09399 | 28.84296 | 4.74E-121 | 2.05E-119 | 265.5929 | UP |
| LINC00602 | 5.618412 | -4.98707 | 28.75579 | 1.49E-120 | 6.36E-119 | 264.4452 | UP |
| CTB-114C7.4 | 4.851069 | -5.62146 | 28.59627 | 1.22E-119 | 5.12E-118 | 262.3442 | UP |
| AC079922.3 | 2.34841 | -0.42862 | 27.9348 | 7.55E-116 | 3.03E-114 | 253.6225 | UP |
| LINC01366 | 5.312872 | -1.46185 | 27.67586 | 2.31E-114 | 8.93E-113 | 250.2049 | UP |
| RP11-42I10.1 | 3.567091 | -0.28521 | 27.43483 | 5.58E-113 | 2.14E-111 | 247.0223 | UP |
| LINC00337 | 4.893592 | -3.12399 | 27.39194 | 9.83E-113 | 3.75E-111 | 246.4559 | UP |
| RP11-400N13.3 | 7.903654 | -3.90986 | 27.35739 | 1.55E-112 | 5.83E-111 | 245.9996 | UP |
| RP13-349O20.2 | 6.287199 | -2.93226 | 27.23807 | 7.51E-112 | 2.76E-110 | 244.4235 | UP |
| RP1-155D22.2 | 6.393643 | -5.02249 | 26.99111 | 1.97E-110 | 6.98E-109 | 241.1607 | UP |
| CTD-3035D6.2 | 6.817015 | -4.84651 | 26.84535 | 1.35E-109 | 4.73E-108 | 239.2347 | UP |
| AC145110.1 | 7.593223 | -2.75323 | 26.68842 | 1.08E-108 | 3.66E-107 | 237.1609 | UP |
| RP13-766D20.4 | 5.855072 | -4.30168 | 26.56329 | 5.64E-108 | 1.90E-106 | 235.5071 | UP |
| RP1-40E16.9 | 6.442667 | -2.1318 | 26.56072 | 5.83E-108 | 1.96E-106 | 235.4732 | UP |
| LINC01293 | 6.538667 | -2.77449 | 26.29633 | 1.93E-106 | 6.15E-105 | 231.9789 | UP |
| AC093850.2 | 6.769128 | -4.15662 | 26.24108 | 4.00E-106 | 1.26E-104 | 231.2487 | UP |
| RP3-522D1.1 | 6.041368 | -1.17923 | 26.13671 | 1.59E-105 | 4.98E-104 | 229.8694 | UP |
| FAM225A | 4.137088 | -0.96216 | 26.06016 | 4.38E-105 | 1.35E-103 | 228.8576 | UP |
| AC003092.1 | 7.182702 | -5.05343 | 26.01095 | 8.40E-105 | 2.58E-103 | 228.2073 | UP |
| CTC-756D1.2 | 5.473236 | -5.39437 | 25.92356 | 2.67E-104 | 8.08E-103 | 227.0525 | UP |
| LINC01272 | 3.305224 | 2.25122 | 25.90029 | 3.63E-104 | 1.09E-102 | 226.745 | UP |
| RP11-529E10.7 | 7.267808 | -1.74243 | 25.88182 | 4.63E-104 | 1.39E-102 | 226.5009 | UP |
| RP4-718J7.4 | 8.469797 | -4.29206 | 25.79769 | 1.41E-103 | 4.16E-102 | 225.3893 | UP |
| RP11-480D4.6 | 5.490015 | -3.17438 | 25.54429 | 4.02E-102 | 1.16E-100 | 222.0415 | UP |
| RP11-38M8.1 | 5.195196 | -1.49043 | 25.25276 | 1.90E-100 | 5.29E-99 | 218.1915 | UP |
| RP11-1055B8.2 | 6.507646 | -3.65709 | 24.91211 | 1.71E-98 | 4.66E-97 | 213.6952 | UP |
| RP11-221N13.3 | 6.615466 | -1.78663 | 24.85225 | 3.76E-98 | 1.02E-96 | 212.9055 | UP |
| HCCAT5 | 6.398906 | -5.22407 | 24.78944 | 8.63E-98 | 2.32E-96 | 212.0769 | UP |
| AC109826.1 | 4.405241 | -3.168 | 24.71222 | 2.39E-97 | 6.39E-96 | 211.0584 | UP |
| LINC00466 | 5.11735 | -5.60857 | 24.62867 | 7.20E-97 | 1.90E-95 | 209.9567 | UP |
| RP11-480D4.2 | 6.082754 | -3.99313 | 24.5694 | 1.57E-96 | 4.12E-95 | 209.1753 | UP |
| RP11-598F7.6 | 4.42457 | -3.16042 | 24.52061 | 3.00E-96 | 7.79E-95 | 208.5322 | UP |
| RP11-223C24.1 | 6.442242 | -3.7285 | 24.48751 | 4.64E-96 | 1.20E-94 | 208.0959 | UP |
| RP11-521C20.2 | 5.160514 | -3.53293 | 24.41386 | 1.23E-95 | 3.14E-94 | 207.1253 | UP |
| CTC-527H23.3 | 5.311857 | -3.61937 | 24.06584 | 1.20E-93 | 3.01E-92 | 202.5425 | UP |
| LINC00473 | 4.999697 | 0.604647 | 23.94163 | 6.18E-93 | 1.51E-91 | 200.9083 | UP |
| FIRRE | 4.149972 | -3.90104 | 23.69334 | 1.62E-91 | 3.83E-90 | 197.6441 | UP |
| RP1-65J11.1 | 5.06022 | -3.63804 | 23.11833 | 3.10E-88 | 6.94E-87 | 190.0993 | UP |
| RP1-257A7.5 | 2.222791 | 3.35223 | 22.85225 | 1.01E-86 | 2.22E-85 | 186.6161 | UP |
| CASC15 | 2.71415 | 1.065162 | 22.71375 | 6.20E-86 | 1.32E-84 | 184.8053 | UP |
| RP11-359E10.1 | 2.884191 | 0.334219 | 22.67914 | 9.76E-86 | 2.07E-84 | 184.3531 | UP |
| RP11-94H18.1 | 6.124835 | -3.63532 | 22.67365 | 1.05E-85 | 2.20E-84 | 184.2813 | UP |
| CH507-513H4.5 | 5.144955 | -0.19472 | 22.45399 | 1.85E-84 | 3.83E-83 | 181.4136 | UP |
| RP11-315A16.1 | 6.662785 | -3.67452 | 22.35838 | 6.45E-84 | 1.32E-82 | 180.1668 | UP |
| RP11-5P4.1 | 5.980276 | -5.97895 | 22.35488 | 6.75E-84 | 1.38E-82 | 180.1211 | UP |
| AC008060.8 | 5.562979 | -6.13907 | 22.30335 | 1.32E-83 | 2.69E-82 | 179.4496 | UP |
| LINC00520 | 7.364138 | -0.02615 | 22.28911 | 1.59E-83 | 3.22E-82 | 179.264 | UP |
| AC144835.1 | 5.716311 | -4.24616 | 22.18264 | 6.39E-83 | 1.27E-81 | 177.8773 | UP |
| RP11-806H10.4 | 5.210634 | -2.19345 | 22.17732 | 6.84E-83 | 1.36E-81 | 177.8081 | UP |
| RP11-472G21.2 | 5.361192 | -6.33179 | 22.09661 | 1.96E-82 | 3.86E-81 | 176.7577 | UP |
| RP11-222K16.2 | 3.987261 | -1.91932 | 22.05586 | 3.33E-82 | 6.55E-81 | 176.2276 | UP |
| RP11-756G20.1 | 4.765787 | -4.11232 | 21.98753 | 8.11E-82 | 1.58E-80 | 175.3392 | UP |
| LINC00494 | 5.14333 | -5.06182 | 21.92522 | 1.82E-81 | 3.51E-80 | 174.5294 | UP |
| ERICD | 2.109581 | -1.56955 | 21.85057 | 4.82E-81 | 9.16E-80 | 173.5598 | UP |
| SUCLG2-AS1 | 3.183014 | -1.07996 | 21.77803 | 1.24E-80 | 2.33E-79 | 172.6183 | UP |
| RP11-680F20.10 | 4.532985 | -3.40024 | 21.77123 | 1.35E-80 | 2.54E-79 | 172.5301 | UP |
| AC104820.2 | 5.208379 | -5.74553 | 21.73006 | 2.31E-80 | 4.32E-79 | 171.9961 | UP |
| RP11-61A14.4 | 3.35893 | -4.67414 | 21.71833 | 2.68E-80 | 5.01E-79 | 171.8439 | UP |
| AP000289.6 | 4.860686 | -6.46149 | 21.65164 | 6.38E-80 | 1.18E-78 | 170.9793 | UP |
| RP11-11N9.4 | 4.921688 | -0.47267 | 21.64627 | 6.84E-80 | 1.26E-78 | 170.9096 | UP |
| LINC01521 | 2.171736 | -0.30416 | 21.46582 | 7.10E-79 | 1.30E-77 | 168.5729 | UP |
| AC010967.2 | 6.951866 | -4.19315 | 21.3947 | 1.78E-78 | 3.23E-77 | 167.653 | UP |
| XXbac-BPG55C20.7 | 6.094209 | -5.30661 | 21.38694 | 1.97E-78 | 3.56E-77 | 167.5528 | UP |
| CTC-548K16.1 | 5.715089 | -4.88166 | 21.35134 | 3.13E-78 | 5.63E-77 | 167.0925 | UP |
| RP11-725P16.2 | 3.567537 | -2.40095 | 21.1616 | 3.64E-77 | 6.43E-76 | 164.6426 | UP |
| RP4-784A16.5 | 5.166668 | -5.89343 | 21.11898 | 6.31E-77 | 1.11E-75 | 164.0929 | UP |
| RP11-98G7.1 | 5.028578 | -4.77997 | 21.04505 | 1.64E-76 | 2.86E-75 | 163.14 | UP |
| LINC01608 | 5.922131 | -5.86894 | 20.99886 | 2.97E-76 | 5.16E-75 | 162.545 | UP |
| CTC-756D1.3 | 4.563585 | -5.95836 | 20.97595 | 3.99E-76 | 6.87E-75 | 162.2501 | UP |
| HCG20 | 4.447027 | -2.08081 | 20.94365 | 6.06E-76 | 1.04E-74 | 161.8343 | UP |
| LINC00944 | 5.76915 | -3.48944 | 20.4576 | 3.13E-73 | 5.21E-72 | 155.5959 | UP |
| LINC01356 | 3.980238 | -2.54426 | 20.31578 | 1.93E-72 | 3.18E-71 | 153.7826 | UP |
| AC009784.3 | 5.555464 | -5.33257 | 20.26773 | 3.56E-72 | 5.86E-71 | 153.1688 | UP |
| LINC01545 | 5.042599 | -4.63787 | 20.23993 | 5.08E-72 | 8.34E-71 | 152.8139 | UP |
| RP5-963E22.6 | 4.828598 | -3.32817 | 20.23752 | 5.24E-72 | 8.58E-71 | 152.7832 | UP |
| FLJ36000 | 5.127865 | -6.19071 | 20.22998 | 5.77E-72 | 9.43E-71 | 152.6869 | UP |
| FLVCR1-AS1 | 2.617572 | 0.493391 | 20.14349 | 1.74E-71 | 2.81E-70 | 151.5839 | UP |
| RP11-121A8.1 | 3.696387 | -4.53034 | 19.9747 | 1.50E-70 | 2.37E-69 | 149.4347 | UP |
| RP4-555D20.2 | 2.911519 | -1.0827 | 19.88681 | 4.59E-70 | 7.17E-69 | 148.3175 | UP |
| RP4-777L9.2 | 4.488904 | -4.87659 | 19.81339 | 1.17E-69 | 1.82E-68 | 147.3852 | UP |
| RP11-343D24.2 | 5.553953 | -6.26316 | 19.79756 | 1.43E-69 | 2.21E-68 | 147.1844 | UP |
| RP11-838N2.5 | 5.700078 | -4.71006 | 19.76454 | 2.17E-69 | 3.35E-68 | 146.7656 | UP |
| RP11-59E19.4 | 6.483202 | -5.53656 | 19.71673 | 3.99E-69 | 6.12E-68 | 146.1595 | UP |
| CTD-2377D24.6 | 4.435489 | -5.33573 | 19.60207 | 1.71E-68 | 2.58E-67 | 144.7076 | UP |
| RP11-90P13.1 | 3.65656 | -5.70626 | 19.46935 | 9.17E-68 | 1.37E-66 | 143.0302 | UP |
| LINC00461 | 4.104753 | -3.16674 | 19.42736 | 1.56E-67 | 2.31E-66 | 142.5002 | UP |
| CTC-529P8.1 | 4.995871 | -3.24432 | 19.32585 | 5.62E-67 | 8.24E-66 | 141.2203 | UP |
| AC011294.3 | 4.483421 | -1.14192 | 19.24757 | 1.51E-66 | 2.21E-65 | 140.2346 | UP |
| RP11-112L6.3 | 4.624522 | -4.42576 | 19.22374 | 2.04E-66 | 2.97E-65 | 139.9349 | UP |
| RP11-886D15.1 | 6.666423 | -5.19568 | 19.21323 | 2.33E-66 | 3.38E-65 | 139.8026 | UP |
| LINC01116 | 2.217826 | 2.080446 | 19.13793 | 6.00E-66 | 8.69E-65 | 138.8563 | UP |
| CTD-2376I4.2 | 3.589758 | -2.02367 | 19.06435 | 1.51E-65 | 2.16E-64 | 137.9326 | UP |
| RP11-26J3.1 | 4.102081 | -5.94858 | 19.02153 | 2.59E-65 | 3.70E-64 | 137.3956 | UP |
| RP11-231N3.1 | 5.446304 | -6.13494 | 18.914 | 1.00E-64 | 1.41E-63 | 136.0488 | UP |
| RP11-621L6.3 | 4.591015 | -1.88513 | 18.87718 | 1.59E-64 | 2.23E-63 | 135.588 | UP |
| AC005281.1 | 4.702496 | -6.80954 | 18.77769 | 5.52E-64 | 7.65E-63 | 134.3449 | UP |
| SPRY4-IT1 | 5.068507 | -2.92159 | 18.7616 | 6.75E-64 | 9.33E-63 | 134.144 | UP |
| TRBV11-2 | 5.822019 | -4.34243 | 18.70396 | 1.39E-63 | 1.90E-62 | 133.425 | UP |
| RP11-131J3.1 | 4.942098 | -4.60298 | 18.69304 | 1.59E-63 | 2.17E-62 | 133.2889 | UP |
| AP000569.9 | 4.970507 | -5.48596 | 18.68585 | 1.74E-63 | 2.37E-62 | 133.1993 | UP |
| LINC01505 | 4.780787 | -5.93736 | 18.67525 | 1.98E-63 | 2.70E-62 | 133.0671 | UP |
| RP11-511B23.3 | 4.675078 | -5.55825 | 18.61677 | 4.12E-63 | 5.56E-62 | 132.3387 | UP |
| RP11-930P14.2 | 2.040397 | -1.91818 | 18.57198 | 7.20E-63 | 9.68E-62 | 131.7814 | UP |
| LINC00326 | 4.770348 | -6.48067 | 18.56943 | 7.43E-63 | 9.98E-62 | 131.7496 | UP |
| AC010894.3 | 2.292036 | 0.746786 | 18.52109 | 1.36E-62 | 1.81E-61 | 131.1487 | UP |
| EPHA5-AS1 | 5.595557 | -3.77081 | 18.46579 | 2.70E-62 | 3.58E-61 | 130.4617 | UP |
| bP-2189O9.3 | 5.812082 | -5.13551 | 18.39331 | 6.65E-62 | 8.75E-61 | 129.5625 | UP |
| LINC00346 | 2.220371 | -1.51316 | 18.33404 | 1.39E-61 | 1.81E-60 | 128.8281 | UP |
| C16orf47 | 4.208734 | -4.28983 | 18.25763 | 3.58E-61 | 4.63E-60 | 127.8825 | UP |
| RP3-332B22.1 | 4.99798 | -2.39812 | 18.21898 | 5.78E-61 | 7.44E-60 | 127.4048 | UP |
| RP11-650K20.3 | 4.259247 | -4.12574 | 18.19764 | 7.53E-61 | 9.62E-60 | 127.1412 | UP |
| RP11-236B18.5 | 5.888902 | -5.8787 | 18.19231 | 8.04E-61 | 1.02E-59 | 127.0753 | UP |
| RP3-369A17.4 | 4.948676 | -6.13867 | 18.18558 | 8.74E-61 | 1.11E-59 | 126.9922 | UP |
| RP11-626K17.3 | 2.455042 | -8.09003 | 18.12617 | 1.82E-60 | 2.29E-59 | 126.2589 | UP |
| AC112721.2 | 4.838323 | -6.68045 | 18.01241 | 7.42E-60 | 9.23E-59 | 124.8574 | UP |
| LINC00355 | 5.332584 | -6.28621 | 17.98222 | 1.08E-59 | 1.33E-58 | 124.486 | UP |
| LINC01281 | 4.386706 | -6.13633 | 17.95915 | 1.43E-59 | 1.77E-58 | 124.2024 | UP |
| AC010987.6 | 4.740205 | -5.80888 | 17.93728 | 1.87E-59 | 2.31E-58 | 123.9336 | UP |
| LINC01444 | 5.245707 | -4.63724 | 17.90947 | 2.64E-59 | 3.24E-58 | 123.592 | UP |
| RP11-557L19.1 | 3.905802 | -3.53181 | 17.89728 | 3.07E-59 | 3.75E-58 | 123.4424 | UP |
| LINC01285 | 3.897745 | -4.82132 | 17.87894 | 3.84E-59 | 4.69E-58 | 123.2172 | UP |
| AC005624.2 | 4.922651 | -5.4707 | 17.87267 | 4.15E-59 | 5.06E-58 | 123.1402 | UP |
| CTD-2561B21.11 | 2.903604 | -1.63293 | 17.83953 | 6.24E-59 | 7.56E-58 | 122.7337 | UP |
| RP11-333O1.1 | 4.089685 | -6.00688 | 17.82991 | 7.02E-59 | 8.49E-58 | 122.6158 | UP |
| AC074391.1 | 3.855681 | -4.89526 | 17.82381 | 7.57E-59 | 9.14E-58 | 122.541 | UP |
| RP11-37L2.1 | 5.446205 | -3.89234 | 17.79952 | 1.02E-58 | 1.23E-57 | 122.2433 | UP |
| CTD-2357A8.3 | 4.789268 | -5.64601 | 17.74541 | 1.98E-58 | 2.37E-57 | 121.5808 | UP |
| LINC00996 | 3.867784 | -4.49707 | 17.70339 | 3.32E-58 | 3.95E-57 | 121.0668 | UP |
| RP1-290I10.3 | 4.345888 | -6.68709 | 17.5878 | 1.37E-57 | 1.61E-56 | 119.6552 | UP |
| RP11-473M10.3 | 5.123247 | -6.06155 | 17.56047 | 1.91E-57 | 2.24E-56 | 119.322 | UP |
| LINC00488 | 4.688268 | -6.75404 | 17.50031 | 3.97E-57 | 4.64E-56 | 118.5893 | UP |
| ELFN1-AS1 | 5.785637 | -2.63807 | 17.47429 | 5.46E-57 | 6.32E-56 | 118.2725 | UP |
| AP000704.5 | 4.544311 | -4.06783 | 17.45044 | 7.30E-57 | 8.43E-56 | 117.9825 | UP |
| RP11-472N13.3 | 4.794019 | -0.51978 | 17.37577 | 1.81E-56 | 2.07E-55 | 117.0753 | UP |
| RP11-27G22.1 | 4.561125 | -6.92505 | 17.35875 | 2.23E-56 | 2.54E-55 | 116.8688 | UP |
| HAGLROS | 3.30411 | -1.35763 | 17.33008 | 3.16E-56 | 3.59E-55 | 116.5209 | UP |
| RP4-771M4.3 | 4.455782 | -6.51134 | 17.28345 | 5.57E-56 | 6.30E-55 | 115.9559 | UP |
| RP11-114M1.2 | 5.409117 | -2.94525 | 17.27905 | 5.87E-56 | 6.64E-55 | 115.9025 | UP |
| RP11-483I13.2 | 4.655332 | -4.79416 | 17.24566 | 8.81E-56 | 9.92E-55 | 115.4983 | UP |
| LINC01291 | 4.226668 | -0.34825 | 17.22095 | 1.19E-55 | 1.34E-54 | 115.1993 | UP |
| AF127936.5 | 4.510117 | -4.90963 | 17.21951 | 1.21E-55 | 1.36E-54 | 115.1819 | UP |
| RP11-138J23.1 | 5.782166 | -5.97093 | 17.21541 | 1.27E-55 | 1.42E-54 | 115.1324 | UP |
| CTD-2341M24.1 | 3.371822 | -2.02819 | 17.1909 | 1.71E-55 | 1.91E-54 | 114.836 | UP |
| RP11-1055B8.3 | 3.673028 | 2.213606 | 17.16723 | 2.28E-55 | 2.54E-54 | 114.55 | UP |
| LINC01531 | 5.466509 | -2.88322 | 17.1115 | 4.47E-55 | 4.94E-54 | 113.8772 | UP |
| RP11-396O20.2 | 4.767872 | -6.18763 | 17.08652 | 6.05E-55 | 6.66E-54 | 113.576 | UP |
| EWSAT1 | 3.423137 | -1.07218 | 17.07795 | 6.71E-55 | 7.36E-54 | 113.4726 | UP |
| RP11-357H14.17 | 4.354667 | -6.26414 | 17.06951 | 7.43E-55 | 8.13E-54 | 113.3709 | UP |
| LINC00221 | 6.447185 | -5.21674 | 17.0529 | 9.08E-55 | 9.89E-54 | 113.1707 | UP |
| RP11-90J7.2 | 4.777663 | -6.53603 | 17.05213 | 9.17E-55 | 9.97E-54 | 113.1615 | UP |
| RP11-197K6.1 | 5.850608 | -5.58661 | 17.03577 | 1.12E-54 | 1.21E-53 | 112.9644 | UP |
| AC012360.4 | 4.014491 | -5.36607 | 17.00054 | 1.71E-54 | 1.84E-53 | 112.5403 | UP |
| LINC00698 | 4.834098 | -6.36275 | 16.93994 | 3.55E-54 | 3.79E-53 | 111.8118 | UP |
| CCDC26 | 3.860557 | -5.33399 | 16.92404 | 4.30E-54 | 4.58E-53 | 111.6208 | UP |
| LINC01194 | 5.371781 | -6.34209 | 16.83336 | 1.28E-53 | 1.35E-52 | 110.5331 | UP |
| RP11-190C22.8 | 4.334376 | -4.93887 | 16.82908 | 1.35E-53 | 1.42E-52 | 110.4817 | UP |
| LINC00977 | 3.948182 | -6.36454 | 16.65916 | 1.03E-52 | 1.07E-51 | 108.4503 | UP |
| RP11-1070N10.5 | 4.633391 | -6.79423 | 16.65881 | 1.04E-52 | 1.07E-51 | 108.4461 | UP |
| LINC01050 | 4.405511 | -6.16376 | 16.59594 | 2.20E-52 | 2.25E-51 | 107.6967 | UP |
| AP003900.6 | 5.096045 | -6.54204 | 16.58124 | 2.62E-52 | 2.68E-51 | 107.5217 | UP |
| LINC01270 | 2.972682 | -1.52751 | 16.55753 | 3.48E-52 | 3.55E-51 | 107.2395 | UP |
| LINC01563 | 4.560064 | -2.05628 | 16.52384 | 5.19E-52 | 5.27E-51 | 106.8389 | UP |
| RP11-6N13.1 | 4.277296 | -6.772 | 16.50173 | 6.76E-52 | 6.85E-51 | 106.5761 | UP |
| LINC01220 | 3.229378 | -2.62872 | 16.49644 | 7.20E-52 | 7.26E-51 | 106.5133 | UP |
| CTD-2376I4.1 | 3.226222 | -3.24442 | 16.46187 | 1.09E-51 | 1.09E-50 | 106.1028 | UP |
| L3MBTL4-AS1 | 2.805746 | -3.52246 | 16.44578 | 1.32E-51 | 1.32E-50 | 105.9119 | UP |
| AC004540.5 | 2.177689 | 0.323012 | 16.44436 | 1.34E-51 | 1.34E-50 | 105.8951 | UP |
| LINC01556 | 4.523554 | -5.66312 | 16.43062 | 1.58E-51 | 1.58E-50 | 105.7321 | UP |
| RP1-224A6.3 | 3.949616 | -4.97448 | 16.41289 | 1.95E-51 | 1.94E-50 | 105.5219 | UP |
| OVAAL | 4.822083 | -6.53432 | 16.375 | 3.05E-51 | 3.02E-50 | 105.073 | UP |
| RP11-1070N10.7 | 5.043784 | -6.51892 | 16.36662 | 3.37E-51 | 3.33E-50 | 104.9739 | UP |
| RP11-1102P16.1 | 4.456962 | -6.72335 | 16.30974 | 6.62E-51 | 6.50E-50 | 104.3012 | UP |
| AC108463.1 | 4.645822 | -4.98548 | 16.29187 | 8.18E-51 | 8.02E-50 | 104.0899 | UP |
| SPATA13 | 3.461403 | -6.05095 | 16.2233 | 1.84E-50 | 1.79E-49 | 103.2807 | UP |
| RP11-1008C21.2 | 2.011301 | -0.54177 | 16.17228 | 3.37E-50 | 3.27E-49 | 102.6796 | UP |
| XXbac-B444P24.14 | 4.034957 | -4.05697 | 16.15597 | 4.08E-50 | 3.95E-49 | 102.4876 | UP |
| RP11-517P14.2 | 2.252975 | -0.4363 | 16.12951 | 5.57E-50 | 5.38E-49 | 102.1763 | UP |
| CTD-2227I18.1 | 4.471405 | -6.64728 | 16.11895 | 6.31E-50 | 6.07E-49 | 102.0522 | UP |
| LINC01443 | 5.306707 | -1.38262 | 16.06199 | 1.24E-49 | 1.18E-48 | 101.3831 | UP |
| RP11-439M11.1 | 4.410511 | -4.10868 | 16.06066 | 1.25E-49 | 1.19E-48 | 101.3675 | UP |
| LINC01146 | 4.001027 | -5.29198 | 16.04165 | 1.57E-49 | 1.49E-48 | 101.1444 | UP |
| AP003774.6 | 3.653458 | -6.64685 | 15.95343 | 4.42E-49 | 4.16E-48 | 100.1109 | UP |
| RP11-109E24.1 | 3.688217 | -7.283 | 15.90668 | 7.66E-49 | 7.15E-48 | 99.56433 | UP |
| RP11-134N1.2 | 4.24747 | -6.78587 | 15.87509 | 1.11E-48 | 1.03E-47 | 99.19541 | UP |
| AE000662.93 | 4.62177 | -5.93407 | 15.86425 | 1.26E-48 | 1.17E-47 | 99.06888 | UP |
| RP11-574H6.1 | 5.218173 | -6.48702 | 15.8578 | 1.36E-48 | 1.26E-47 | 98.99358 | UP |
| RP11-764K9.1 | 3.127427 | -5.84488 | 15.84479 | 1.58E-48 | 1.45E-47 | 98.84186 | UP |
| RP3-388N13.5 | 3.39661 | -4.91643 | 15.81186 | 2.32E-48 | 2.13E-47 | 98.458 | UP |
| RP11-575F12.1 | 4.657126 | -6.28402 | 15.75466 | 4.53E-48 | 4.12E-47 | 97.79205 | UP |
| NBAT1 | 3.258548 | -3.79579 | 15.71787 | 6.96E-48 | 6.33E-47 | 97.3644 | UP |
| LINC00315 | 2.781169 | -5.99537 | 15.65635 | 1.42E-47 | 1.28E-46 | 96.65033 | UP |
| RP11-405M12.4 | 3.355799 | -2.68972 | 15.58982 | 3.08E-47 | 2.76E-46 | 95.87956 | UP |
| SMCR2 | 4.213628 | -4.79033 | 15.58883 | 3.12E-47 | 2.79E-46 | 95.86815 | UP |
| RP11-328N19.1 | 4.409995 | -6.51941 | 15.58802 | 3.15E-47 | 2.81E-46 | 95.85875 | UP |
| AC016700.5 | 2.425957 | -1.19646 | 15.54015 | 5.49E-47 | 4.88E-46 | 95.30518 | UP |
| LINC01446 | 4.450289 | -6.26438 | 15.53966 | 5.52E-47 | 4.90E-46 | 95.29956 | UP |
| RP11-25H12.1 | 4.408541 | -7.00038 | 15.49007 | 9.80E-47 | 8.67E-46 | 94.72704 | UP |
| GS1-600G8.5 | 3.907109 | -5.02965 | 15.43034 | 1.96E-46 | 1.72E-45 | 94.03849 | UP |
| CTD-2314B22.1 | 4.385453 | -6.96779 | 15.41324 | 2.38E-46 | 2.09E-45 | 93.84165 | UP |
| LINC01314 | 3.156934 | 0.122863 | 15.38647 | 3.25E-46 | 2.84E-45 | 93.53368 | UP |
| LINC01516 | 2.943172 | -7.1067 | 15.3609 | 4.36E-46 | 3.80E-45 | 93.23981 | UP |
| LINC00402 | 3.214601 | -7.2783 | 15.3563 | 4.60E-46 | 4.00E-45 | 93.18701 | UP |
| LINC01048 | 4.209222 | -6.33408 | 15.30951 | 7.88E-46 | 6.82E-45 | 92.6499 | UP |
| RP4-655J12.5 | 4.011601 | -6.74019 | 15.30899 | 7.93E-46 | 6.85E-45 | 92.64386 | UP |
| RP11-151A10.3 | 3.579968 | -4.02841 | 15.27702 | 1.14E-45 | 9.85E-45 | 92.27741 | UP |
| RP11-495O10.1 | 3.894189 | -6.98684 | 15.26693 | 1.29E-45 | 1.11E-44 | 92.1618 | UP |
| RP11-493L12.5 | 4.037671 | -7.15528 | 15.26328 | 1.34E-45 | 1.15E-44 | 92.11999 | UP |
| RP5-907D15.4 | 4.001674 | -2.80875 | 15.22401 | 2.10E-45 | 1.80E-44 | 91.6705 | UP |
| AC005019.3 | 4.686507 | -6.38812 | 15.20814 | 2.53E-45 | 2.15E-44 | 91.48895 | UP |
| RP11-10J5.1 | 4.015105 | -7.14339 | 15.13028 | 6.16E-45 | 5.23E-44 | 90.60001 | UP |
| RP11-358B23.1 | 3.897752 | -7.03821 | 15.12129 | 6.83E-45 | 5.79E-44 | 90.49751 | UP |
| CTD-2171N6.1 | 3.188798 | -4.88834 | 15.11024 | 7.75E-45 | 6.56E-44 | 90.37163 | UP |
| RP11-318G21.4 | 5.426359 | -5.47783 | 15.09397 | 9.33E-45 | 7.87E-44 | 90.18628 | UP |
| CTC-548K16.2 | 3.853298 | -6.25759 | 15.07293 | 1.19E-44 | 9.99E-44 | 89.94665 | UP |
| RP5-1068H6.6 | 4.00567 | -6.9991 | 15.06405 | 1.31E-44 | 1.10E-43 | 89.84564 | UP |
| PKNOX2-AS1 | 3.650926 | -7.39182 | 15.06094 | 1.36E-44 | 1.14E-43 | 89.81031 | UP |
| RP11-542M13.3 | 3.861892 | -6.78837 | 15.05179 | 1.51E-44 | 1.26E-43 | 89.70622 | UP |
| LINC01597 | 3.321941 | -5.82406 | 15.03732 | 1.78E-44 | 1.49E-43 | 89.54166 | UP |
| RP11-982M15.8 | 2.401989 | -6.16523 | 15.02672 | 2.01E-44 | 1.67E-43 | 89.42124 | UP |
| RP11-284F21.9 | 2.845286 | -3.27139 | 15.02672 | 2.01E-44 | 1.67E-43 | 89.42115 | UP |
| ERVMER61-1 | 4.421488 | -5.73165 | 15.01162 | 2.39E-44 | 1.98E-43 | 89.24959 | UP |
| RP4-555D20.3 | 3.675535 | -7.3157 | 15.00424 | 2.60E-44 | 2.15E-43 | 89.16589 | UP |
| MYCNUT | 4.218056 | -7.05662 | 14.9768 | 3.55E-44 | 2.93E-43 | 88.85438 | UP |
| FAM225B | 2.78922 | -2.72148 | 14.92233 | 6.60E-44 | 5.42E-43 | 88.2371 | UP |
| LINC01317 | 5.30462 | -2.94057 | 14.8112 | 2.33E-43 | 1.89E-42 | 86.98119 | UP |
| CTD-2337I7.1 | 3.723757 | -6.07142 | 14.78468 | 3.14E-43 | 2.55E-42 | 86.68225 | UP |
| RP11-114H23.1 | 4.137642 | -4.9583 | 14.77242 | 3.61E-43 | 2.92E-42 | 86.54418 | UP |
| RP11-230G5.2 | 4.905844 | -4.06182 | 14.75486 | 4.40E-43 | 3.55E-42 | 86.34639 | UP |
| RP11-368I7.4 | 3.239429 | -4.23856 | 14.73388 | 5.58E-43 | 4.49E-42 | 86.11039 | UP |
| LINC00563 | 2.908461 | -7.8066 | 14.73178 | 5.71E-43 | 4.59E-42 | 86.08673 | UP |
| LINC00460 | 4.589422 | -4.97695 | 14.71784 | 6.68E-43 | 5.36E-42 | 85.92993 | UP |
| RP11-108L7.15 | 2.515468 | -2.59723 | 14.65323 | 1.38E-42 | 1.11E-41 | 85.20454 | UP |
| AC112721.1 | 3.828225 | -7.23954 | 14.63226 | 1.75E-42 | 1.40E-41 | 84.96936 | UP |
| CTA-384D8.34 | 4.537388 | -3.63547 | 14.61914 | 2.03E-42 | 1.62E-41 | 84.82236 | UP |
| LINC00629 | 3.725025 | -5.1855 | 14.50965 | 6.94E-42 | 5.48E-41 | 83.59834 | UP |
| GS1-279B7.2 | 2.211872 | -1.86858 | 14.48718 | 8.93E-42 | 7.03E-41 | 83.34779 | UP |
| RP1-228P16.3 | 3.371143 | -7.65952 | 14.47606 | 1.01E-41 | 7.95E-41 | 83.22389 | UP |
| RP11-288L9.1 | 4.05452 | -6.20136 | 14.46207 | 1.18E-41 | 9.28E-41 | 83.06803 | UP |
| RP11-136O12.2 | 3.27251 | -7.72277 | 14.42534 | 1.78E-41 | 1.40E-40 | 82.65928 | UP |
| LINC00505 | 4.619014 | -6.01428 | 14.39019 | 2.64E-41 | 2.06E-40 | 82.26855 | UP |
| RP5-933K21.3 | 3.423889 | -3.50953 | 14.36978 | 3.31E-41 | 2.59E-40 | 82.04197 | UP |
| RP11-8L8.2 | 3.25627 | -4.71201 | 14.36781 | 3.38E-41 | 2.64E-40 | 82.02013 | UP |
| RP11-419K12.1 | 3.894725 | -6.40276 | 14.3604 | 3.68E-41 | 2.87E-40 | 81.93781 | UP |
| RP11-254F7.4 | 4.238664 | -5.87098 | 14.30226 | 7.02E-41 | 5.43E-40 | 81.29348 | UP |
| LINC01034 | 4.515032 | -6.72589 | 14.29266 | 7.81E-41 | 6.03E-40 | 81.18732 | UP |
| AC007952.4 | 3.399652 | -5.45408 | 14.28807 | 8.22E-41 | 6.33E-40 | 81.13651 | UP |
| LINC01143 | 4.123895 | -4.85362 | 14.25729 | 1.16E-40 | 8.89E-40 | 80.79612 | UP |
| RP5-984P4.6 | 2.790653 | -3.48581 | 14.22866 | 1.59E-40 | 1.22E-39 | 80.47995 | UP |
| RP11-214F16.8 | 3.465002 | -3.07927 | 14.21796 | 1.79E-40 | 1.37E-39 | 80.36184 | UP |
| RP11-731F5.2 | 4.533222 | -6.36812 | 14.1904 | 2.43E-40 | 1.85E-39 | 80.05792 | UP |
| RP11-121C6.5 | 2.909655 | -6.10003 | 14.16203 | 3.32E-40 | 2.52E-39 | 79.74535 | UP |
| MIR503HG | 2.554294 | -0.12824 | 14.15137 | 3.74E-40 | 2.83E-39 | 79.62801 | UP |
| RP11-153K16.1 | 3.250517 | -7.49046 | 14.13797 | 4.33E-40 | 3.28E-39 | 79.48056 | UP |
| RP11-579D7.4 | 4.011248 | -4.89258 | 14.10949 | 5.93E-40 | 4.47E-39 | 79.16741 | UP |
| RP11-42O15.3 | 3.232187 | -2.44972 | 14.08142 | 8.09E-40 | 6.07E-39 | 78.85916 | UP |
| RP11-480D4.1 | 2.629884 | -7.87119 | 14.07245 | 8.93E-40 | 6.69E-39 | 78.76074 | UP |
| RP5-884M6.1 | 4.117878 | -6.32195 | 14.01787 | 1.63E-39 | 1.21E-38 | 78.16254 | UP |
| RP11-363E6.4 | 2.072923 | -2.43041 | 14.00547 | 1.87E-39 | 1.38E-38 | 78.02686 | UP |
| AP003774.4 | 2.382607 | -4.36235 | 14.00162 | 1.95E-39 | 1.44E-38 | 77.98473 | UP |
| LINC01021 | 4.258861 | -3.15446 | 13.99865 | 2.01E-39 | 1.49E-38 | 77.95225 | UP |
| RP5-1172N10.2 | 3.258904 | -6.41013 | 13.99842 | 2.02E-39 | 1.49E-38 | 77.94968 | UP |
| LINC01358 | 4.158191 | -4.02506 | 13.99145 | 2.18E-39 | 1.61E-38 | 77.87349 | UP |
| LINC00297 | 2.969218 | -7.95993 | 13.98633 | 2.30E-39 | 1.70E-38 | 77.81752 | UP |
| RP11-302M6.5 | 3.336838 | -7.01875 | 13.94227 | 3.73E-39 | 2.75E-38 | 77.33612 | UP |
| RP3-483K16.4 | 3.456207 | -3.89692 | 13.92278 | 4.62E-39 | 3.40E-38 | 77.12356 | UP |
| SFTA1P | 3.072356 | -2.78231 | 13.92185 | 4.67E-39 | 3.43E-38 | 77.11338 | UP |
| RP11-89C3.4 | 4.4575 | -2.70022 | 13.89464 | 6.29E-39 | 4.60E-38 | 76.8168 | UP |
| RP11-335E6.3 | 2.763276 | -8.07382 | 13.88989 | 6.62E-39 | 4.84E-38 | 76.76507 | UP |
| RP11-317N12.1 | 5.104459 | -6.25722 | 13.86193 | 8.99E-39 | 6.55E-38 | 76.4607 | UP |
| RP11-736K20.6 | 2.002593 | -1.74458 | 13.8506 | 1.02E-38 | 7.40E-38 | 76.33749 | UP |
| RP5-875H18.9 | 4.145258 | -4.9149 | 13.84136 | 1.13E-38 | 8.18E-38 | 76.23704 | UP |
| ST3GAL5-AS1 | 2.301805 | -1.45309 | 13.8366 | 1.19E-38 | 8.61E-38 | 76.18533 | UP |
| RP11-48B3.3 | 2.227294 | -3.88722 | 13.8206 | 1.41E-38 | 1.02E-37 | 76.01147 | UP |
| AC133644.2 | 2.114702 | 0.154708 | 13.8147 | 1.51E-38 | 1.09E-37 | 75.94739 | UP |
| RP5-887A10.1 | 4.482069 | -6.2565 | 13.75961 | 2.74E-38 | 1.98E-37 | 75.34994 | UP |
| RP11-218F4.1 | 3.105702 | -7.31426 | 13.71395 | 4.51E-38 | 3.23E-37 | 74.85575 | UP |
| LINC00858 | 3.480631 | -6.91688 | 13.66031 | 8.06E-38 | 5.76E-37 | 74.27646 | UP |
| RP5-906C1.1 | 3.190717 | -1.09146 | 13.64161 | 9.87E-38 | 7.02E-37 | 74.07476 | UP |
| RP11-114H23.2 | 3.738827 | -6.19871 | 13.62047 | 1.24E-37 | 8.80E-37 | 73.84698 | UP |
| RP11-214K3.24 | 2.163669 | -4.92545 | 13.58344 | 1.85E-37 | 1.30E-36 | 73.44854 | UP |
| LINC01198 | 3.419679 | 0.589509 | 13.55111 | 2.62E-37 | 1.84E-36 | 73.10112 | UP |
| LINC01502 | 4.387715 | -6.98179 | 13.54538 | 2.79E-37 | 1.96E-36 | 73.03962 | UP |
| CDKN2A-AS1 | 3.578855 | -6.95735 | 13.52636 | 3.43E-37 | 2.40E-36 | 72.8355 | UP |
| CTB-50L17.16 | 4.046099 | -4.59941 | 13.46996 | 6.29E-37 | 4.39E-36 | 72.23123 | UP |
| RP11-394A14.2 | 3.244078 | -7.37259 | 13.45865 | 7.10E-37 | 4.95E-36 | 72.11028 | UP |
| RP4-529N6.1 | 4.606187 | -0.30964 | 13.44414 | 8.30E-37 | 5.77E-36 | 71.95518 | UP |
| RP11-221J22.1 | 3.722463 | -7.48416 | 13.43664 | 9.00E-37 | 6.25E-36 | 71.87502 | UP |
| MNX1-AS1 | 4.111486 | -5.46456 | 13.39914 | 1.34E-36 | 9.32E-36 | 71.47464 | UP |
| RP11-1152H15.1 | 3.494612 | -7.18733 | 13.37645 | 1.71E-36 | 1.18E-35 | 71.23278 | UP |
| RP11-489O18.1 | 3.69892 | -6.19963 | 13.3734 | 1.77E-36 | 1.22E-35 | 71.20022 | UP |
| RP11-711D18.2 | 3.176022 | -7.67287 | 13.30414 | 3.71E-36 | 2.54E-35 | 70.46347 | UP |
| RP11-527H14.1 | 3.548145 | -6.45322 | 13.29221 | 4.22E-36 | 2.88E-35 | 70.3367 | UP |
| AC022182.3 | 3.539336 | -4.39837 | 13.20584 | 1.06E-35 | 7.17E-35 | 69.42161 | UP |
| RP11-66B24.1 | 3.422577 | -1.15819 | 13.14647 | 1.99E-35 | 1.34E-34 | 68.7946 | UP |
| LINC00941 | 3.298766 | -3.09199 | 13.10869 | 2.96E-35 | 1.98E-34 | 68.39652 | UP |
| AL133493.2 | 2.609956 | -3.92172 | 13.094 | 3.46E-35 | 2.31E-34 | 68.24182 | UP |
| CTD-3157E16.1 | 2.877896 | 0.139585 | 13.05722 | 5.11E-35 | 3.39E-34 | 67.85517 | UP |
| LINC00601 | 2.950294 | -5.7375 | 13.0416 | 6.02E-35 | 3.99E-34 | 67.6912 | UP |
| AJ239322.1 | 2.86412 | -7.96613 | 13.03407 | 6.52E-35 | 4.31E-34 | 67.61218 | UP |
| CTB-41I6.1 | 3.859775 | -5.84249 | 12.97383 | 1.23E-34 | 8.06E-34 | 66.98108 | UP |
| XXbac-B33L19.4 | 3.735915 | -7.14836 | 12.94607 | 1.65E-34 | 1.07E-33 | 66.69083 | UP |
| RP4-777O23.1 | 3.604255 | -7.08418 | 12.93151 | 1.92E-34 | 1.25E-33 | 66.53872 | UP |
| RP11-218E20.3 | 2.848233 | -5.2873 | 12.92078 | 2.15E-34 | 1.39E-33 | 66.42671 | UP |
| LINC00282 | 2.698062 | -3.3753 | 12.90566 | 2.51E-34 | 1.63E-33 | 66.26898 | UP |
| HIF1A-AS1 | 3.208418 | -5.95127 | 12.84509 | 4.74E-34 | 3.06E-33 | 65.63828 | UP |
| RP11-443B7.3 | 2.98666 | -6.06399 | 12.83147 | 5.46E-34 | 3.53E-33 | 65.49671 | UP |
| CTB-151G24.1 | 5.079463 | -3.64667 | 12.82149 | 6.06E-34 | 3.91E-33 | 65.39301 | UP |
| C15orf54 | 2.552389 | -6.86345 | 12.81151 | 6.73E-34 | 4.33E-33 | 65.28932 | UP |
| AC023469.1 | 3.577486 | -7.42613 | 12.7882 | 8.58E-34 | 5.50E-33 | 65.04752 | UP |
| RP11-18B16.2 | 2.966163 | -6.50379 | 12.77627 | 9.71E-34 | 6.21E-33 | 64.92388 | UP |
| PRCAT47 | 2.532938 | -4.97115 | 12.77057 | 1.03E-33 | 6.58E-33 | 64.86472 | UP |
| CTC-459F4.5 | 3.731033 | -7.31977 | 12.76755 | 1.06E-33 | 6.79E-33 | 64.83352 | UP |
| RP11-1072A3.4 | 3.368003 | -2.13813 | 12.75771 | 1.18E-33 | 7.51E-33 | 64.73158 | UP |
| MGC39584 | 4.570742 | -4.83592 | 12.75335 | 1.23E-33 | 7.85E-33 | 64.68641 | UP |
| RP5-899E9.1 | 2.894782 | -3.49422 | 12.71695 | 1.80E-33 | 1.14E-32 | 64.30997 | UP |
| CH507-39O4.2 | 3.227609 | -2.97664 | 12.70808 | 1.97E-33 | 1.25E-32 | 64.2183 | UP |
| RP1-90G24.6 | 4.056199 | -6.47537 | 12.69679 | 2.22E-33 | 1.41E-32 | 64.1017 | UP |
| AP000472.2 | 3.708914 | -7.49319 | 12.69659 | 2.22E-33 | 1.41E-32 | 64.09965 | UP |
| CTD-2116N20.1 | 3.791669 | -6.04132 | 12.68692 | 2.46E-33 | 1.55E-32 | 63.99983 | UP |
| RP11-160E2.16 | 3.068647 | -5.44411 | 12.67425 | 2.80E-33 | 1.77E-32 | 63.86919 | UP |
| RP11-476K15.1 | 3.280109 | -7.75267 | 12.67419 | 2.81E-33 | 1.77E-32 | 63.86857 | UP |
| RP11-218I7.2 | 3.282077 | -7.68378 | 12.6645 | 3.10E-33 | 1.95E-32 | 63.76865 | UP |
| CTB-41I6.2 | 3.392801 | -4.84251 | 12.65929 | 3.27E-33 | 2.06E-32 | 63.71489 | UP |
| AC009495.3 | 3.452106 | -2.39541 | 12.61299 | 5.29E-33 | 3.32E-32 | 63.23839 | UP |
| SALRNA1 | 2.294092 | -7.92411 | 12.59327 | 6.48E-33 | 4.06E-32 | 63.03571 | UP |
| RP11-608O21.1 | 3.249462 | -7.61932 | 12.46534 | 2.42E-32 | 1.50E-31 | 61.72602 | UP |
| RP11-865I6.2 | 3.73741 | -7.00058 | 12.44906 | 2.86E-32 | 1.77E-31 | 61.55995 | UP |
| RP11-480I12.7 | 2.695808 | -3.99489 | 12.40131 | 4.66E-32 | 2.88E-31 | 61.07367 | UP |
| LINC01117 | 2.758533 | -2.13623 | 12.37922 | 5.84E-32 | 3.59E-31 | 60.84911 | UP |
| RP11-93K22.13 | 3.32479 | -5.18246 | 12.34608 | 8.19E-32 | 5.02E-31 | 60.51273 | UP |
| LINC01037 | 3.368034 | -7.57469 | 12.34273 | 8.48E-32 | 5.19E-31 | 60.47874 | UP |
| CTC-492K19.7 | 3.197443 | -5.22309 | 12.34159 | 8.57E-32 | 5.25E-31 | 60.4672 | UP |
| RP11-462L8.1 | 2.758684 | -5.80113 | 12.34031 | 8.69E-32 | 5.32E-31 | 60.45417 | UP |
| RP11-542A14.1 | 3.837188 | -5.71067 | 12.33076 | 9.58E-32 | 5.85E-31 | 60.35742 | UP |
| CH507-152C13.6 | 2.126971 | -8.32568 | 12.31715 | 1.10E-31 | 6.70E-31 | 60.2195 | UP |
| AP001631.10 | 2.126971 | -8.32568 | 12.31715 | 1.10E-31 | 6.70E-31 | 60.2195 | UP |
| RP11-383J24.1 | 3.028035 | -7.73424 | 12.31429 | 1.13E-31 | 6.89E-31 | 60.19051 | UP |
| XXbac-BPG308K3.5 | 3.081532 | -6.32253 | 12.29909 | 1.32E-31 | 8.03E-31 | 60.03674 | UP |
| RP11-624J12.1 | 2.681422 | -7.77562 | 12.29589 | 1.37E-31 | 8.29E-31 | 60.00429 | UP |
| LINC01474 | 4.642225 | -3.14622 | 12.27306 | 1.72E-31 | 1.04E-30 | 59.77351 | UP |
| RP11-145H9.3 | 2.841273 | -7.31911 | 12.26206 | 1.93E-31 | 1.17E-30 | 59.66242 | UP |
| RP11-440G9.1 | 2.712707 | -7.81936 | 12.24758 | 2.23E-31 | 1.35E-30 | 59.5163 | UP |
| RP11-699L21.1 | 2.722617 | -4.19857 | 12.24741 | 2.23E-31 | 1.35E-30 | 59.51458 | UP |
| LINC01399 | 3.077742 | -7.85298 | 12.24476 | 2.30E-31 | 1.38E-30 | 59.48777 | UP |
| RP4-646N3.1 | 2.753774 | -6.6832 | 12.23341 | 2.58E-31 | 1.55E-30 | 59.37339 | UP |
| RP11-744K17.1 | 2.047214 | -8.52675 | 12.21978 | 2.96E-31 | 1.78E-30 | 59.23601 | UP |
| C8orf49 | 3.048037 | -7.52972 | 12.18001 | 4.42E-31 | 2.65E-30 | 58.83568 | UP |
| RP11-1079K10.3 | 3.122553 | -3.7489 | 12.17255 | 4.77E-31 | 2.85E-30 | 58.76074 | UP |
| AC005487.2 | 2.773954 | -7.69348 | 12.14056 | 6.59E-31 | 3.93E-30 | 58.43948 | UP |
| RP11-268G12.1 | 2.786739 | -7.85412 | 12.11355 | 8.65E-31 | 5.15E-30 | 58.16874 | UP |
| RP11-160E2.17 | 2.994727 | -5.7164 | 12.09194 | 1.08E-30 | 6.40E-30 | 57.95233 | UP |
| RP11-255C15.3 | 2.283067 | -4.65556 | 12.08009 | 1.21E-30 | 7.19E-30 | 57.83375 | UP |
| RP11-268G12.3 | 3.148448 | -7.67336 | 12.07415 | 1.29E-30 | 7.62E-30 | 57.77439 | UP |
| RP1-90G24.11 | 4.225313 | -5.67997 | 12.07166 | 1.32E-30 | 7.81E-30 | 57.74948 | UP |
| RP11-575F12.2 | 3.660672 | -6.37141 | 12.02076 | 2.20E-30 | 1.30E-29 | 57.24145 | UP |
| RP5-1070A16.1 | 2.631821 | -8.1115 | 12.01358 | 2.36E-30 | 1.39E-29 | 57.16985 | UP |
| RP11-400N13.1 | 3.036101 | -7.491 | 12.00843 | 2.49E-30 | 1.46E-29 | 57.11859 | UP |
| RP11-815M8.1 | 3.40833 | -6.32951 | 12.00426 | 2.59E-30 | 1.53E-29 | 57.07699 | UP |
| DSCR9 | 2.036882 | -3.47812 | 11.96868 | 3.70E-30 | 2.17E-29 | 56.72301 | UP |
| GS1-279B7.1 | 2.972349 | -7.52402 | 11.92976 | 5.46E-30 | 3.20E-29 | 56.33662 | UP |
| RP11-573G6.9 | 3.10218 | -7.14906 | 11.9104 | 6.63E-30 | 3.88E-29 | 56.14465 | UP |
| RP5-1039K5.18 | 3.119465 | -4.41872 | 11.89462 | 7.75E-30 | 4.52E-29 | 55.98838 | UP |
| RP11-424I19.2 | 3.217399 | -4.27765 | 11.89025 | 8.10E-30 | 4.71E-29 | 55.94515 | UP |
| RP11-78L16.1 | 3.431619 | -7.65166 | 11.87885 | 9.07E-30 | 5.27E-29 | 55.83239 | UP |
| RP11-84D1.2 | 2.48245 | -7.45457 | 11.86749 | 1.02E-29 | 5.89E-29 | 55.7201 | UP |
| LINC00499 | 3.730567 | -7.27367 | 11.86103 | 1.08E-29 | 6.27E-29 | 55.65625 | UP |
| LINC00839 | 2.01688 | 0.369237 | 11.82098 | 1.61E-29 | 9.27E-29 | 55.26096 | UP |
| RP11-3J1.1 | 3.231152 | -7.8117 | 11.75583 | 3.07E-29 | 1.76E-28 | 54.61991 | UP |
| CTA-373H7.7 | 2.698663 | -6.03747 | 11.75554 | 3.08E-29 | 1.76E-28 | 54.61706 | UP |
| RP1-167O22.1 | 2.65533 | -7.72123 | 11.72111 | 4.33E-29 | 2.47E-28 | 54.2792 | UP |
| LINC01318 | 2.792404 | -7.95376 | 11.71488 | 4.60E-29 | 2.62E-28 | 54.2182 | UP |
| LINC01102 | 3.41497 | -2.82297 | 11.71326 | 4.68E-29 | 2.66E-28 | 54.20225 | UP |
| RP11-308B16.2 | 3.25885 | -7.75927 | 11.70628 | 5.01E-29 | 2.84E-28 | 54.13395 | UP |
| LINC01271 | 2.536228 | -4.53553 | 11.69835 | 5.42E-29 | 3.07E-28 | 54.05627 | UP |
| LINC00945 | 2.571973 | -8.19777 | 11.6943 | 5.64E-29 | 3.19E-28 | 54.01657 | UP |
| CTD-2195M15.3 | 3.685638 | -6.85337 | 11.69103 | 5.82E-29 | 3.29E-28 | 53.98461 | UP |
| RP11-260M19.2 | 3.77482 | -5.47714 | 11.67915 | 6.55E-29 | 3.70E-28 | 53.86838 | UP |
| RP11-6J21.2 | 3.06674 | -7.13211 | 11.6772 | 6.67E-29 | 3.76E-28 | 53.84937 | UP |
| RP1-122K4.2 | 3.529851 | -6.9186 | 11.62626 | 1.10E-28 | 6.18E-28 | 53.35201 | UP |
| RP11-332H14.1 | 3.164039 | -2.54214 | 11.62586 | 1.10E-28 | 6.20E-28 | 53.34806 | UP |
| LA16c-83F12.6 | 3.16527 | -7.50807 | 11.60971 | 1.29E-28 | 7.26E-28 | 53.19071 | UP |
| CTB-30L5.1 | 2.994671 | -7.24448 | 11.59667 | 1.47E-28 | 8.23E-28 | 53.06378 | UP |
| RP11-41O4.1 | 2.316368 | -1.71615 | 11.55228 | 2.27E-28 | 1.26E-27 | 52.63234 | UP |
| RP11-63E9.1 | 3.29164 | -4.56429 | 11.53654 | 2.65E-28 | 1.47E-27 | 52.47959 | UP |
| AP000318.2 | 2.950715 | -4.61678 | 11.5133 | 3.32E-28 | 1.84E-27 | 52.25442 | UP |
| RP5-1195D24.1 | 2.738184 | -7.94949 | 11.5043 | 3.63E-28 | 2.00E-27 | 52.16722 | UP |
| AC015849.16 | 2.310874 | -5.89791 | 11.50126 | 3.74E-28 | 2.06E-27 | 52.13779 | UP |
| RP11-221N13.4 | 3.473566 | -7.29477 | 11.46697 | 5.21E-28 | 2.87E-27 | 51.80633 | UP |
| LINC01501 | 2.708259 | -8.06704 | 11.43053 | 7.43E-28 | 4.07E-27 | 51.45473 | UP |
| RP4-806M20.3 | 2.668407 | -6.56482 | 11.43005 | 7.46E-28 | 4.08E-27 | 51.45016 | UP |
| RP11-66B24.2 | 2.696339 | -1.82793 | 11.39967 | 1.00E-27 | 5.46E-27 | 51.15763 | UP |
| RP11-114G22.1 | 3.148545 | -5.35488 | 11.39959 | 1.00E-27 | 5.46E-27 | 51.15691 | UP |
| RP11-30P6.6 | 2.365847 | -7.05509 | 11.34174 | 1.75E-27 | 9.54E-27 | 50.60142 | UP |
| AP000476.1 | 2.484024 | -5.08376 | 11.27884 | 3.21E-27 | 1.74E-26 | 49.99958 | UP |
| RP11-180M15.6 | 2.000897 | -2.40103 | 11.27469 | 3.35E-27 | 1.81E-26 | 49.95996 | UP |
| RP11-128P17.2 | 3.372877 | -7.44123 | 11.25983 | 3.86E-27 | 2.08E-26 | 49.81817 | UP |
| RP11-1149M10.2 | 2.904113 | -3.70028 | 11.22014 | 5.65E-27 | 3.04E-26 | 49.44008 | UP |
| AC074363.1 | 2.719284 | -7.97298 | 11.21159 | 6.13E-27 | 3.29E-26 | 49.35882 | UP |
| RP11-973H7.4 | 2.870101 | -7.41992 | 11.18953 | 7.57E-27 | 4.06E-26 | 49.14919 | UP |
| RP11-586D19.1 | 2.139452 | -7.63649 | 11.18797 | 7.68E-27 | 4.12E-26 | 49.13438 | UP |
| CTD-2587H24.10 | 2.044383 | -2.88833 | 11.16415 | 9.64E-27 | 5.16E-26 | 48.90829 | UP |
| RP11-982M15.6 | 2.49682 | -3.62999 | 11.15322 | 1.07E-26 | 5.72E-26 | 48.80472 | UP |
| LINC01518 | 3.594465 | -7.32648 | 11.14526 | 1.15E-26 | 6.16E-26 | 48.72934 | UP |
| LINC00052 | 3.171226 | -6.44559 | 11.13624 | 1.26E-26 | 6.71E-26 | 48.64391 | UP |
| RP11-116N8.4 | 2.814073 | -7.94558 | 11.09423 | 1.88E-26 | 9.94E-26 | 48.24688 | UP |
| LINC00645 | 2.774135 | -7.31727 | 11.08804 | 1.99E-26 | 1.05E-25 | 48.18846 | UP |
| RP6-91H8.3 | 2.487891 | -7.14381 | 11.08001 | 2.15E-26 | 1.13E-25 | 48.11269 | UP |
| RP11-344P13.6 | 3.377719 | -5.58574 | 11.07379 | 2.28E-26 | 1.20E-25 | 48.054 | UP |
| RP5-1011O1.3 | 2.865777 | -7.94488 | 11.06472 | 2.48E-26 | 1.31E-25 | 47.96851 | UP |
| KIAA0125 | 3.639358 | -3.35254 | 11.03784 | 3.21E-26 | 1.68E-25 | 47.7155 | UP |
| AC017002.1 | 2.235448 | -2.05582 | 10.95613 | 6.94E-26 | 3.61E-25 | 46.94894 | UP |
| AC012506.2 | 2.172513 | -8.44016 | 10.9421 | 7.92E-26 | 4.12E-25 | 46.81766 | UP |
| RP11-814H16.2 | 3.09292 | -5.76072 | 10.93993 | 8.08E-26 | 4.20E-25 | 46.79741 | UP |
| RP11-805F19.2 | 2.87988 | -7.89087 | 10.93285 | 8.64E-26 | 4.48E-25 | 46.73121 | UP |
| LINC00456 | 3.213204 | -6.90562 | 10.93133 | 8.76E-26 | 4.54E-25 | 46.71709 | UP |
| RP11-643G5.6 | 3.826551 | -7.33494 | 10.92751 | 9.08E-26 | 4.71E-25 | 46.68134 | UP |
| CH507-210P18.4 | 2.824383 | -7.92897 | 10.88932 | 1.30E-25 | 6.70E-25 | 46.32516 | UP |
| RP4-811H24.9 | 2.645421 | -7.03922 | 10.88694 | 1.33E-25 | 6.84E-25 | 46.30296 | UP |
| RP11-289F5.1 | 2.438531 | -8.00302 | 10.87446 | 1.50E-25 | 7.69E-25 | 46.18675 | UP |
| RP11-1055B8.10 | 3.094419 | -7.2349 | 10.87209 | 1.53E-25 | 7.86E-25 | 46.16467 | UP |
| RP11-25L3.3 | 2.448027 | -8.33378 | 10.82824 | 2.30E-25 | 1.18E-24 | 45.75717 | UP |
| RP11-561B11.6 | 2.529273 | -8.22311 | 10.82126 | 2.46E-25 | 1.25E-24 | 45.69243 | UP |
| CASC9 | 3.471191 | -6.42973 | 10.81523 | 2.60E-25 | 1.32E-24 | 45.63649 | UP |
| AC145343.2 | 2.356251 | -2.38124 | 10.73949 | 5.27E-25 | 2.67E-24 | 44.93608 | UP |
| RP5-834N19.1 | 3.076638 | -7.55565 | 10.73373 | 5.56E-25 | 2.81E-24 | 44.88296 | UP |
| XXbac-B33L19.6 | 2.207731 | -8.35658 | 10.69695 | 7.82E-25 | 3.94E-24 | 44.54419 | UP |
| LINC01419 | 3.058221 | -7.81894 | 10.65289 | 1.18E-24 | 5.91E-24 | 44.13952 | UP |
| AC006129.2 | 2.723955 | -1.83603 | 10.63854 | 1.34E-24 | 6.73E-24 | 44.00789 | UP |
| RP11-702B10.2 | 2.453366 | -7.74428 | 10.6233 | 1.55E-24 | 7.73E-24 | 43.86839 | UP |
| RP11-314N14.1 | 3.2534 | -7.73288 | 10.59732 | 1.96E-24 | 9.77E-24 | 43.63075 | UP |
| LINC00703 | 2.002166 | -8.23875 | 10.57815 | 2.34E-24 | 1.16E-23 | 43.45574 | UP |
| RP11-404O13.1 | 2.61931 | -5.45172 | 10.56627 | 2.61E-24 | 1.30E-23 | 43.34741 | UP |
| RP11-21K12.3 | 2.609326 | -5.56787 | 10.54094 | 3.30E-24 | 1.63E-23 | 43.1166 | UP |
| AC008991.1 | 3.659437 | -4.54397 | 10.51607 | 4.14E-24 | 2.05E-23 | 42.89048 | UP |
| LINC00972 | 2.758597 | -8.10128 | 10.51329 | 4.25E-24 | 2.10E-23 | 42.86522 | UP |
| RP11-966I7.2 | 2.277293 | -8.32485 | 10.51131 | 4.33E-24 | 2.13E-23 | 42.84719 | UP |
| AC062028.1 | 2.673967 | -6.53448 | 10.50391 | 4.63E-24 | 2.28E-23 | 42.78003 | UP |
| LINC01602 | 2.143987 | -8.3565 | 10.50076 | 4.76E-24 | 2.35E-23 | 42.75139 | UP |
| RP11-553A10.1 | 2.87967 | -4.86725 | 10.49843 | 4.87E-24 | 2.40E-23 | 42.73031 | UP |
| RP11-715J22.4 | 2.348512 | -3.29369 | 10.4963 | 4.96E-24 | 2.44E-23 | 42.71092 | UP |
| RP11-417E7.2 | 3.095486 | -4.92068 | 10.49096 | 5.21E-24 | 2.56E-23 | 42.6625 | UP |
| CTD-2540M10.1 | 2.901719 | -7.99295 | 10.49059 | 5.23E-24 | 2.57E-23 | 42.65915 | UP |
| RP11-626H12.2 | 3.237457 | -5.71299 | 10.48894 | 5.31E-24 | 2.61E-23 | 42.64422 | UP |
| RP4-655J12.4 | 2.734101 | -2.79239 | 10.46712 | 6.48E-24 | 3.17E-23 | 42.4464 | UP |
| AC061992.2 | 2.679826 | -4.21401 | 10.38669 | 1.35E-23 | 6.53E-23 | 41.7202 | UP |
| RP11-428G5.5 | 2.828145 | -6.08065 | 10.36108 | 1.70E-23 | 8.21E-23 | 41.48979 | UP |
| CTB-118N6.2 | 2.933903 | -5.68143 | 10.35854 | 1.74E-23 | 8.39E-23 | 41.46696 | UP |
| AC012506.3 | 2.486682 | -8.19264 | 10.34829 | 1.91E-23 | 9.18E-23 | 41.37486 | UP |
| BSN-AS2 | 2.066979 | -7.2886 | 10.33582 | 2.14E-23 | 1.03E-22 | 41.26297 | UP |
| AC016710.1 | 2.729536 | -8.14611 | 10.32769 | 2.30E-23 | 1.10E-22 | 41.18997 | UP |
| RP11-94B19.5 | 2.443199 | -8.11128 | 10.32615 | 2.33E-23 | 1.12E-22 | 41.17621 | UP |
| RP11-19N8.4 | 2.839041 | -4.49911 | 10.31149 | 2.66E-23 | 1.27E-22 | 41.04482 | UP |
| CTD-2078B5.2 | 3.103241 | -3.20719 | 10.29619 | 3.06E-23 | 1.46E-22 | 40.90787 | UP |
| RP1-65P5.5 | 2.753339 | -7.54564 | 10.24189 | 4.98E-23 | 2.37E-22 | 40.42299 | UP |
| KB-1592A4.14 | 2.048039 | -8.60044 | 10.21675 | 6.24E-23 | 2.96E-22 | 40.19918 | UP |
| RP11-89C3.3 | 3.03703 | -6.67076 | 10.21568 | 6.30E-23 | 2.99E-22 | 40.18963 | UP |
| LINC01393 | 2.315398 | -4.27319 | 10.21497 | 6.34E-23 | 3.00E-22 | 40.18329 | UP |
| RP11-408N14.1 | 2.884793 | -7.98007 | 10.21394 | 6.40E-23 | 3.03E-22 | 40.17418 | UP |
| RP11-117D22.2 | 2.664185 | -6.03414 | 10.20215 | 7.12E-23 | 3.36E-22 | 40.06933 | UP |
| RP11-220I1.5 | 2.19832 | -7.39823 | 10.17653 | 8.95E-23 | 4.22E-22 | 39.84184 | UP |
| RP11-25I15.3 | 2.223718 | -7.9411 | 10.14296 | 1.21E-22 | 5.68E-22 | 39.54442 | UP |
| RP11-807H7.2 | 3.068286 | -6.93183 | 10.13189 | 1.33E-22 | 6.25E-22 | 39.44651 | UP |
| RP11-316A16.1 | 2.070495 | -8.55689 | 10.09456 | 1.86E-22 | 8.70E-22 | 39.117 | UP |
| LINC00862 | 2.442282 | -5.82181 | 10.09162 | 1.91E-22 | 8.93E-22 | 39.09106 | UP |
| AP000479.1 | 2.433334 | -8.11039 | 10.08274 | 2.07E-22 | 9.66E-22 | 39.0128 | UP |
| AC008175.1 | 2.094495 | -8.49244 | 10.07406 | 2.23E-22 | 1.04E-21 | 38.9364 | UP |
| AC007359.6 | 2.07008 | -8.50871 | 10.05713 | 2.59E-22 | 1.21E-21 | 38.78741 | UP |
| LINC01055 | 2.078268 | -7.81794 | 10.04519 | 2.88E-22 | 1.34E-21 | 38.68248 | UP |
| AC093590.1 | 2.460589 | -8.00763 | 10.04343 | 2.93E-22 | 1.36E-21 | 38.66705 | UP |
| RP11-701P16.5 | 2.700174 | -5.28935 | 10.04196 | 2.97E-22 | 1.38E-21 | 38.65412 | UP |
| RP11-366H4.1 | 3.114808 | -7.60767 | 10.04113 | 2.99E-22 | 1.39E-21 | 38.64679 | UP |
| LINC01098 | 2.442825 | -8.14162 | 10.01077 | 3.91E-22 | 1.82E-21 | 38.3805 | UP |
| RP11-1114I9.1 | 2.257622 | -8.32785 | 9.989821 | 4.70E-22 | 2.18E-21 | 38.19709 | UP |
| RP11-753N8.1 | 2.748532 | -7.81453 | 9.969171 | 5.64E-22 | 2.61E-21 | 38.01658 | UP |
| CTA-390C10.9 | 2.72249 | -6.81865 | 9.935348 | 7.60E-22 | 3.51E-21 | 37.72152 | UP |
| RP11-479J7.2 | 2.50331 | -7.94278 | 9.910409 | 9.46E-22 | 4.36E-21 | 37.50445 | UP |
| CTC-480C2.1 | 2.24947 | -8.31318 | 9.891454 | 1.12E-21 | 5.13E-21 | 37.33973 | UP |
| RP11-160N1.10 | 2.525745 | -7.35951 | 9.881076 | 1.22E-21 | 5.61E-21 | 37.24965 | UP |
| AC018742.1 | 2.03645 | -8.31147 | 9.865412 | 1.40E-21 | 6.42E-21 | 37.11382 | UP |
| CTD-2228A4.1 | 2.783988 | -7.30624 | 9.853921 | 1.55E-21 | 7.09E-21 | 37.01428 | UP |
| RP11-563N12.2 | 2.705231 | -7.51133 | 9.836367 | 1.81E-21 | 8.25E-21 | 36.86239 | UP |
| RP5-1166F10.1 | 2.242318 | -7.87905 | 9.809832 | 2.28E-21 | 1.04E-20 | 36.63318 | UP |
| AL109763.2 | 2.42508 | -8.32187 | 9.791036 | 2.68E-21 | 1.22E-20 | 36.4711 | UP |
| RP4-550H1.7 | 3.012899 | -6.76494 | 9.769538 | 3.24E-21 | 1.46E-20 | 36.28601 | UP |
| RP11-655G22.1 | 2.494332 | -8.30291 | 9.766826 | 3.31E-21 | 1.50E-20 | 36.26268 | UP |
| FAM215A | 2.355078 | -7.38992 | 9.764173 | 3.39E-21 | 1.53E-20 | 36.23987 | UP |
| RP11-430H10.1 | 2.606533 | -2.81768 | 9.761166 | 3.48E-21 | 1.57E-20 | 36.21402 | UP |
| RP11-431M3.1 | 2.555059 | -8.17162 | 9.747806 | 3.91E-21 | 1.76E-20 | 36.09922 | UP |
| RP11-128P17.4 | 2.789294 | -8.10627 | 9.741998 | 4.11E-21 | 1.85E-20 | 36.04936 | UP |
| RP11-768F21.1 | 2.246986 | -3.3318 | 9.711955 | 5.33E-21 | 2.39E-20 | 35.79176 | UP |
| RP11-156K13.3 | 2.184653 | -8.12891 | 9.709602 | 5.44E-21 | 2.44E-20 | 35.77162 | UP |
| LINC00243 | 2.197235 | -5.31653 | 9.706237 | 5.60E-21 | 2.51E-20 | 35.74281 | UP |
| RP11-342K6.4 | 2.128317 | -3.80112 | 9.66722 | 7.84E-21 | 3.50E-20 | 35.40933 | UP |
| RP11-567G11.1 | 2.936783 | -7.84225 | 9.666512 | 7.89E-21 | 3.52E-20 | 35.4033 | UP |
| AC007391.2 | 2.536284 | -7.06635 | 9.666152 | 7.91E-21 | 3.53E-20 | 35.40022 | UP |
| RP11-545M17.1 | 2.516953 | -7.05574 | 9.658688 | 8.44E-21 | 3.76E-20 | 35.33655 | UP |
| CTB-60B18.10 | 2.609818 | -2.51651 | 9.607175 | 1.31E-20 | 5.83E-20 | 34.89815 | UP |
| CTD-2134P3.1 | 2.971673 | -7.88926 | 9.586901 | 1.56E-20 | 6.93E-20 | 34.7261 | UP |
| DPH6-AS1 | 2.305253 | -3.84109 | 9.585433 | 1.58E-20 | 7.02E-20 | 34.71365 | UP |
| RP11-337A23.6 | 2.401844 | -8.32988 | 9.571324 | 1.79E-20 | 7.90E-20 | 34.5941 | UP |
| MIR2052HG | 2.459989 | -7.54011 | 9.566019 | 1.87E-20 | 8.26E-20 | 34.54918 | UP |
| LA16c-325D7.2 | 2.286952 | -3.33384 | 9.559993 | 1.97E-20 | 8.69E-20 | 34.49818 | UP |
| RP11-180C1.1 | 2.823476 | -7.99586 | 9.553658 | 2.08E-20 | 9.17E-20 | 34.44459 | UP |
| AB015752.3 | 2.379034 | -7.29351 | 9.54414 | 2.25E-20 | 9.92E-20 | 34.36413 | UP |
| AC073409.1 | 2.705305 | -8.12318 | 9.524029 | 2.67E-20 | 1.18E-19 | 34.19433 | UP |
| CTD-2532N20.1 | 2.464537 | -4.96335 | 9.518826 | 2.79E-20 | 1.23E-19 | 34.15044 | UP |
| RP11-809H16.5 | 2.580916 | -6.8971 | 9.502203 | 3.22E-20 | 1.41E-19 | 34.01035 | UP |
| LINC01320 | 3.176623 | -4.09044 | 9.499634 | 3.29E-20 | 1.44E-19 | 33.98872 | UP |
| RP11-79O8.1 | 2.449933 | -4.84395 | 9.478623 | 3.93E-20 | 1.72E-19 | 33.81196 | UP |
| AC092580.3 | 2.396289 | -7.88919 | 9.471539 | 4.18E-20 | 1.83E-19 | 33.75243 | UP |
| LINC01150 | 3.101678 | -5.98682 | 9.471136 | 4.19E-20 | 1.83E-19 | 33.74905 | UP |
| RP11-335E6.4 | 2.445208 | -7.55029 | 9.458019 | 4.68E-20 | 2.05E-19 | 33.63891 | UP |
| RP11-318E3.9 | 3.24223 | -4.64592 | 9.428337 | 6.02E-20 | 2.63E-19 | 33.39014 | UP |
| XXyac-YM21GA2.4 | 2.573433 | -8.09461 | 9.420438 | 6.44E-20 | 2.81E-19 | 33.32404 | UP |
| LINC01611 | 2.801594 | -7.44472 | 9.404181 | 7.38E-20 | 3.21E-19 | 33.18813 | UP |
| RP5-1102E8.3 | 2.585089 | -6.97001 | 9.373797 | 9.54E-20 | 4.14E-19 | 32.93461 | UP |
| RP11-202G18.1 | 2.270034 | -7.74433 | 9.357664 | 1.09E-19 | 4.72E-19 | 32.80025 | UP |
| RP11-455B3.1 | 2.684829 | -6.53977 | 9.356488 | 1.10E-19 | 4.76E-19 | 32.79046 | UP |
| AC009495.4 | 3.073941 | -3.18461 | 9.316284 | 1.55E-19 | 6.65E-19 | 32.45646 | UP |
| MIR137HG | 2.54389 | -7.18068 | 9.315069 | 1.56E-19 | 6.71E-19 | 32.44639 | UP |
| RP11-138M12.1 | 2.93372 | -7.40615 | 9.313696 | 1.58E-19 | 6.79E-19 | 32.435 | UP |
| CTD-2591A6.2 | 2.326984 | -8.41448 | 9.295359 | 1.84E-19 | 7.90E-19 | 32.28307 | UP |
| CTC-565M22.1 | 2.313997 | -8.38944 | 9.253007 | 2.62E-19 | 1.12E-18 | 31.93305 | UP |
| UNQ6494 | 2.332684 | -5.35911 | 9.244147 | 2.83E-19 | 1.20E-18 | 31.85998 | UP |
| CTD-2130F23.2 | 2.356287 | -7.8386 | 9.237116 | 3.00E-19 | 1.28E-18 | 31.80203 | UP |
| RP11-774D14.1 | 2.206804 | -8.29472 | 9.221132 | 3.42E-19 | 1.45E-18 | 31.67044 | UP |
| RP11-110I1.14 | 2.375011 | -2.89218 | 9.216071 | 3.57E-19 | 1.52E-18 | 31.62881 | UP |
| RP1-232L24.3 | 2.188257 | -8.25856 | 9.211078 | 3.72E-19 | 1.58E-18 | 31.58775 | UP |
| RP11-838N2.3 | 2.142991 | -8.33711 | 9.2089 | 3.79E-19 | 1.61E-18 | 31.56985 | UP |
| LINC00463 | 2.140469 | -8.08363 | 9.153184 | 6.01E-19 | 2.53E-18 | 31.11301 | UP |
| RP11-292E2.2 | 2.74925 | -6.99794 | 9.136825 | 6.88E-19 | 2.89E-18 | 30.97928 | UP |
| C10orf126 | 2.569536 | -7.25671 | 9.108783 | 8.67E-19 | 3.63E-18 | 30.7505 | UP |
| RP11-960L18.1 | 2.644344 | -5.22339 | 9.10708 | 8.79E-19 | 3.68E-18 | 30.73662 | UP |
| RP11-546K22.3 | 2.504323 | -3.29064 | 9.10654 | 8.83E-19 | 3.70E-18 | 30.73222 | UP |
| LINC01486 | 2.018909 | -8.40496 | 9.097298 | 9.53E-19 | 3.98E-18 | 30.65696 | UP |
| RP11-90K6.1 | 2.623916 | -4.21319 | 9.088203 | 1.03E-18 | 4.29E-18 | 30.58295 | UP |
| LINC00861 | 2.034829 | -2.78581 | 9.059422 | 1.30E-18 | 5.41E-18 | 30.34913 | UP |
| RP11-704J17.5 | 2.481734 | -7.82999 | 9.05556 | 1.34E-18 | 5.58E-18 | 30.3178 | UP |
| RP4-777L9.3 | 2.163088 | -8.27637 | 9.04698 | 1.44E-18 | 5.98E-18 | 30.24823 | UP |
| RP11-13A1.1 | 2.072732 | -5.66147 | 9.035809 | 1.58E-18 | 6.55E-18 | 30.15773 | UP |
| RP11-445P19.3 | 3.041714 | -6.31345 | 8.944573 | 3.32E-18 | 1.36E-17 | 29.42193 | UP |
| DLX2-AS1 | 2.010075 | -7.79766 | 8.923121 | 3.95E-18 | 1.62E-17 | 29.24978 | UP |
| RP4-610C12.3 | 2.803433 | -2.85458 | 8.911788 | 4.33E-18 | 1.77E-17 | 29.15897 | UP |
| CTD-2023N9.3 | 2.301423 | -6.9805 | 8.882633 | 5.49E-18 | 2.23E-17 | 28.92577 | UP |
| RP4-580N22.2 | 3.35841 | -6.46856 | 8.880744 | 5.57E-18 | 2.26E-17 | 28.91069 | UP |
| RP11-374M1.5 | 2.883584 | -4.64673 | 8.850512 | 7.11E-18 | 2.88E-17 | 28.66956 | UP |
| RP11-398H6.1 | 2.15961 | -4.69401 | 8.835081 | 8.05E-18 | 3.26E-17 | 28.54674 | UP |
| AC003005.2 | 2.557115 | -6.56755 | 8.812818 | 9.63E-18 | 3.89E-17 | 28.36984 | UP |
| CTA-126B4.7 | 2.011991 | -8.14475 | 8.81163 | 9.72E-18 | 3.93E-17 | 28.36041 | UP |
| RP11-348J12.5 | 2.089841 | -8.19385 | 8.80691 | 1.01E-17 | 4.07E-17 | 28.32295 | UP |
| RP11-346D14.1 | 2.115551 | -4.65182 | 8.778837 | 1.26E-17 | 5.08E-17 | 28.10051 | UP |
| AL928768.3 | 3.256613 | -2.53988 | 8.762282 | 1.44E-17 | 5.79E-17 | 27.9696 | UP |
| RP11-521C20.5 | 2.402096 | -6.73101 | 8.731712 | 1.84E-17 | 7.37E-17 | 27.7284 | UP |
| AC000067.1 | 2.249115 | -8.20131 | 8.730356 | 1.86E-17 | 7.45E-17 | 27.71771 | UP |
| AC011524.1 | 2.945056 | -4.80159 | 8.711947 | 2.16E-17 | 8.60E-17 | 27.5728 | UP |
| RP1-156L9.1 | 2.343326 | -8.30194 | 8.711233 | 2.17E-17 | 8.65E-17 | 27.56719 | UP |
| RP11-320G24.1 | 2.141903 | -6.38828 | 8.695078 | 2.47E-17 | 9.81E-17 | 27.44023 | UP |
| RP11-30J20.1 | 3.50148 | -4.36888 | 8.635861 | 3.94E-17 | 1.56E-16 | 26.97649 | UP |
| RP11-369C8.1 | 3.567223 | -4.38544 | 8.60363 | 5.08E-17 | 2.00E-16 | 26.72516 | UP |
| RP5-1125N11.1 | 2.02203 | -8.61778 | 8.599912 | 5.23E-17 | 2.06E-16 | 26.69621 | UP |
| RP3-527G5.1 | 3.344603 | -5.80449 | 8.569998 | 6.62E-17 | 2.59E-16 | 26.4637 | UP |
| RP11-98D18.17 | 2.372722 | -5.41248 | 8.566363 | 6.82E-17 | 2.67E-16 | 26.4355 | UP |
| RP11-353N4.6 | 2.2496 | -5.70052 | 8.521132 | 9.72E-17 | 3.79E-16 | 26.08531 | UP |
| FAM230A | 2.092067 | -7.98111 | 8.51067 | 1.05E-16 | 4.11E-16 | 26.00453 | UP |
| LINC00870 | 2.047701 | -6.89289 | 8.496092 | 1.18E-16 | 4.59E-16 | 25.89209 | UP |
| LL22NC03-N95F10.1 | 2.523248 | -6.80507 | 8.494872 | 1.19E-16 | 4.63E-16 | 25.88269 | UP |
| RP11-344P13.4 | 2.222391 | -8.23004 | 8.492092 | 1.22E-16 | 4.73E-16 | 25.86126 | UP |
| AP001058.3 | 2.499349 | -6.77955 | 8.475639 | 1.39E-16 | 5.37E-16 | 25.73461 | UP |
| LINC01506 | 2.763881 | -5.9136 | 8.474787 | 1.39E-16 | 5.40E-16 | 25.72806 | UP |
| AC003088.1 | 2.14905 | -7.87173 | 8.413678 | 2.24E-16 | 8.61E-16 | 25.25942 | UP |
| RP11-354I10.1 | 2.06836 | -7.95715 | 8.395545 | 2.58E-16 | 9.88E-16 | 25.12089 | UP |
| RP11-133F8.2 | 2.006119 | -8.62839 | 8.379939 | 2.91E-16 | 1.11E-15 | 25.00186 | UP |
| RP11-105C19.1 | 2.089544 | -8.00374 | 8.348449 | 3.71E-16 | 1.42E-15 | 24.76224 | UP |
| LINC01096 | 2.253627 | -5.69435 | 8.347552 | 3.74E-16 | 1.42E-15 | 24.75543 | UP |
| CTD-3224K15.3 | 2.318523 | -4.81713 | 8.338637 | 4.00E-16 | 1.52E-15 | 24.68773 | UP |
| CTB-61M7.2 | 2.226976 | -4.31522 | 8.30431 | 5.21E-16 | 1.98E-15 | 24.42761 | UP |
| CTD-2384A14.1 | 2.437456 | -7.87346 | 8.29028 | 5.80E-16 | 2.20E-15 | 24.32155 | UP |
| CTD-2144E22.8 | 2.463019 | -8.10071 | 8.264203 | 7.08E-16 | 2.68E-15 | 24.12481 | UP |
| CTD-2240J17.1 | 2.287459 | -7.33975 | 8.251884 | 7.77E-16 | 2.94E-15 | 24.03205 | UP |
| RP1-46F2.3 | 2.176682 | -8.34563 | 8.251104 | 7.82E-16 | 2.95E-15 | 24.02617 | UP |
| RP11-493L12.4 | 2.625243 | -7.05968 | 8.236309 | 8.75E-16 | 3.30E-15 | 23.91492 | UP |
| LINC01580 | 2.322508 | -7.89457 | 8.181415 | 1.33E-15 | 4.97E-15 | 23.50358 | UP |
| LINC00398 | 2.134314 | -5.65837 | 8.151111 | 1.67E-15 | 6.22E-15 | 23.27748 | UP |
| RP11-573G6.10 | 2.440949 | -7.75142 | 8.138583 | 1.84E-15 | 6.82E-15 | 23.1842 | UP |
| RP11-81H3.2 | 2.268932 | -7.95354 | 8.122056 | 2.08E-15 | 7.70E-15 | 23.06134 | UP |
| RP11-269F21.3 | 2.042889 | -8.45377 | 8.102315 | 2.41E-15 | 8.90E-15 | 22.91486 | UP |
| RP5-942I16.1 | 2.255485 | -6.1516 | 8.075395 | 2.95E-15 | 1.08E-14 | 22.71558 | UP |
| CTD-2023M8.1 | 2.054616 | -8.55675 | 8.070702 | 3.06E-15 | 1.12E-14 | 22.68089 | UP |
| RP5-1172A22.1 | 2.315209 | -4.87086 | 8.050069 | 3.57E-15 | 1.30E-14 | 22.5286 | UP |
| RP11-412P11.1 | 2.017961 | -8.26335 | 7.963595 | 6.79E-15 | 2.45E-14 | 21.89385 | UP |
| RP11-260A9.6 | 2.371646 | -8.0722 | 7.920323 | 9.35E-15 | 3.36E-14 | 21.57837 | UP |
| CTD-2036P10.3 | 2.112826 | -3.32159 | 7.89151 | 1.16E-14 | 4.14E-14 | 21.36909 | UP |
| RP11-359I18.5 | 2.176104 | -3.42074 | 7.881246 | 1.25E-14 | 4.46E-14 | 21.2947 | UP |
| RP13-49I15.6 | 2.406165 | -5.19475 | 7.861766 | 1.44E-14 | 5.13E-14 | 21.15373 | UP |
| AC069277.2 | 2.285698 | -7.27541 | 7.859631 | 1.46E-14 | 5.21E-14 | 21.13829 | UP |
| RP1-315G1.3 | 2.238651 | -2.42067 | 7.850992 | 1.56E-14 | 5.54E-14 | 21.07588 | UP |
| RP11-338H14.1 | 2.167462 | -6.34349 | 7.826337 | 1.87E-14 | 6.62E-14 | 20.89809 | UP |
| RP11-185E8.1 | 2.310955 | -8.03726 | 7.813152 | 2.05E-14 | 7.29E-14 | 20.8032 | UP |
| RP11-80F22.14 | 2.362398 | -8.09391 | 7.798426 | 2.29E-14 | 8.09E-14 | 20.69738 | UP |
| AC106706.1 | 2.542914 | -6.76927 | 7.724799 | 3.90E-14 | 1.37E-13 | 20.17081 | UP |
| RP11-72M17.1 | 2.181374 | -2.53312 | 7.648686 | 6.75E-14 | 2.34E-13 | 19.63089 | UP |
| CH507-145C22.4 | 2.084776 | -6.53722 | 7.592092 | 1.01E-13 | 3.49E-13 | 19.23236 | UP |
| KB-1980E6.3 | 2.18789 | -5.72622 | 7.450663 | 2.75E-13 | 9.29E-13 | 18.24743 | UP |
| CTC-350I8.1 | 2.033779 | -7.26867 | 7.413938 | 3.56E-13 | 1.20E-12 | 17.99425 | UP |
| CTC-273B12.10 | 2.191108 | -5.87408 | 7.280435 | 9.00E-13 | 2.97E-12 | 17.08291 | UP |
| RP11-488I20.8 | 2.486689 | -6.93858 | 7.206871 | 1.49E-12 | 4.89E-12 | 16.58681 | UP |
| CTD-2291D10.4 | 2.42841 | -5.11876 | 7.185044 | 1.73E-12 | 5.65E-12 | 16.44045 | UP |
| RP1-223B1.1 | 2.045884 | -6.49808 | 7.164905 | 1.98E-12 | 6.46E-12 | 16.30575 | UP |
| RP11-95P2.3 | 2.203925 | -2.89308 | 7.083483 | 3.44E-12 | 1.11E-11 | 15.76448 | UP |
| XX-CR54.1 | 2.513959 | -7.51594 | 7.070548 | 3.75E-12 | 1.21E-11 | 15.67899 | UP |
| RP11-781A6.1 | 2.224856 | -5.69516 | 7.038254 | 4.66E-12 | 1.50E-11 | 15.46612 | UP |
| LINC01033 | 2.073309 | -7.30331 | 7.013586 | 5.50E-12 | 1.76E-11 | 15.3041 | UP |
| CTC-498J12.1 | 2.177639 | -6.24905 | 7.011156 | 5.59E-12 | 1.79E-11 | 15.28816 | UP |
| RP11-542A14.2 | 2.160691 | -8.16273 | 6.958133 | 7.96E-12 | 2.54E-11 | 14.94168 | UP |
| RP11-419C23.1 | 2.046274 | -5.24912 | 6.897453 | 1.19E-11 | 3.76E-11 | 14.54796 | UP |
| RP11-348F1.2 | 2.019898 | -7.1259 | 6.884657 | 1.29E-11 | 4.08E-11 | 14.46532 | UP |
| CTC-246B18.10 | 2.846659 | -0.71044 | 6.851212 | 1.61E-11 | 5.06E-11 | 14.24995 | UP |
| RP11-680H20.2 | 2.020232 | -6.43264 | 6.81786 | 2.00E-11 | 6.27E-11 | 14.03609 | UP |
| RP11-675F6.3 | 2.197008 | -6.7716 | 6.69967 | 4.30E-11 | 1.33E-10 | 13.28562 | UP |
| RP11-680G10.1 | 2.071867 | -6.72377 | 6.36264 | 3.59E-10 | 1.07E-09 | 11.20923 | UP |
| CTC-338M12.9 | 2.033726 | -7.11445 | 6.326972 | 4.47E-10 | 1.32E-09 | 10.99506 | UP |
| AC092580.4 | 2.057677 | -0.73848 | 5.60822 | 2.95E-08 | 7.90E-08 | 6.910705 | UP |
| RMRP | 2.093841 | -6.64807 | 4.769405 | 2.25E-06 | 5.41E-06 | 2.716709 | UP |
| CTD-2626G11.2 | -10.302 | -5.92659 | -88.6332 | 0 | 0 | 865.5865 | DOWN |
| RP11-145E17.2 | -10.6017 | -5.77988 | -76.2522 | 0 | 0 | 770.7282 | DOWN |
| KB-1991G8.1 | -9.84811 | -6.19652 | -75.6084 | 0 | 0 | 765.4626 | DOWN |
| RP11-297D21.2 | -10.2983 | -6.08588 | -74.5548 | 0 | 0 | 756.7694 | DOWN |
| RP11-372M18.2 | -11.4157 | -5.83847 | -69.8609 | 3.27142998252423e-317 | 5.01641073510384e-314 | 716.8366 | DOWN |
| LSINCT5 | -6.65446 | -7.37033 | -64.7545 | 2.80E-297 | 3.58E-294 | 671.0275 | DOWN |
| AC008074.4 | -10.703 | -5.47674 | -62.4999 | 4.08E-288 | 4.46E-285 | 649.964 | DOWN |
| LINC01514 | -7.15521 | -7.27724 | -60.4318 | 1.66E-279 | 1.59E-276 | 630.1683 | DOWN |
| RP11-4K16.2 | -8.6489 | -6.43027 | -59.6953 | 2.16E-276 | 1.84E-273 | 623.0064 | DOWN |
| RP11-386B13.4 | -10.6834 | -5.24828 | -57.6191 | 1.81E-267 | 1.38E-264 | 602.4909 | DOWN |
| WI2-85898F10.1 | -9.48307 | -6.15581 | -57.4594 | 8.94E-267 | 5.71E-264 | 600.8927 | DOWN |
| RP1-140J1.1 | -7.64785 | -6.86647 | -57.405 | 1.54E-266 | 9.10E-264 | 600.3479 | DOWN |
| RP5-1056H1.2 | -9.71157 | -5.31529 | -56.9651 | 1.29E-264 | 7.06E-262 | 595.9281 | DOWN |
| CTD-2008P7.9 | -8.89815 | -5.46851 | -56.3733 | 5.15E-262 | 2.63E-259 | 589.9457 | DOWN |
| AC006262.4 | -10.726 | -5.40249 | -56.1157 | 7.06E-261 | 3.38E-258 | 587.3302 | DOWN |
| RP11-259O2.1 | -9.03428 | -6.01473 | -55.6307 | 1.00E-258 | 4.51E-256 | 582.3826 | DOWN |
| GATA3-AS1 | -9.01907 | -5.27461 | -54.734 | 1.02E-254 | 4.34E-252 | 573.1643 | DOWN |
| RP11-60A8.1 | -9.35634 | -5.90345 | -54.2283 | 1.93E-252 | 7.80E-250 | 567.9244 | DOWN |
| RP4-737E23.2 | -12.1632 | -3.10605 | -53.7473 | 2.92E-250 | 1.12E-247 | 562.9115 | DOWN |
| RP11-734K21.5 | -6.62616 | -7.22632 | -53.7406 | 3.14E-250 | 1.14E-247 | 562.8408 | DOWN |
| RP11-646E18.4 | -7.66785 | -6.70235 | -53.7007 | 4.76E-250 | 1.66E-247 | 562.4238 | DOWN |
| RP11-465B22.8 | -10.6242 | -4.46065 | -52.1265 | 7.90E-243 | 2.63E-240 | 545.8157 | DOWN |
| CTA-398F10.2 | -9.2943 | -5.95318 | -51.6406 | 1.42E-240 | 4.54E-238 | 540.6283 | DOWN |
| RP11-255G12.2 | -8.43139 | -6.05432 | -50.2689 | 3.85E-234 | 1.18E-231 | 525.8296 | DOWN |
| RP1-124C6.1 | -9.92247 | -5.49244 | -50.0686 | 3.41E-233 | 1.01E-230 | 523.6495 | DOWN |
| LINC01254 | -6.66407 | -7.22122 | -50.0228 | 5.63E-233 | 1.60E-230 | 523.1505 | DOWN |
| RP11-464C19.3 | -8.95609 | -6.01154 | -49.8333 | 4.46E-232 | 1.22E-229 | 521.0817 | DOWN |
| RP11-3P17.4 | -7.10853 | -6.24647 | -49.2449 | 2.84E-229 | 7.51E-227 | 514.6309 | DOWN |
| LINC01348 | -7.52648 | -5.55255 | -49.0532 | 2.35E-228 | 6.00E-226 | 512.5204 | DOWN |
| AC131097.4 | -8.93482 | -5.72163 | -48.8185 | 3.14E-227 | 7.77E-225 | 509.9289 | DOWN |
| TPTEP1 | -5.35277 | 1.09411 | -48.6503 | 2.02E-226 | 4.84E-224 | 508.0689 | DOWN |
| CH17-360D5.3 | -10.6928 | -4.55458 | -47.7126 | 6.96E-222 | 1.62E-219 | 497.6299 | DOWN |
| AP000439.3 | -8.84435 | -5.93752 | -47.2988 | 7.25E-220 | 1.63E-217 | 492.988 | DOWN |
| RP11-521D12.5 | -9.05547 | -4.86304 | -47.2615 | 1.10E-219 | 2.41E-217 | 492.5695 | DOWN |
| CH17-360D5.2 | -11.4995 | -3.59484 | -46.7724 | 2.75E-217 | 5.87E-215 | 487.0525 | DOWN |
| RP11-485M7.3 | -9.84565 | -5.27669 | -45.2872 | 6.30E-210 | 1.27E-207 | 470.1184 | DOWN |
| RP11-439H13.2 | -6.76334 | -7.47166 | -45.2154 | 1.44E-209 | 2.83E-207 | 469.2931 | DOWN |
| RP11-304F15.4 | -5.43496 | -7.93107 | -45.0444 | 1.03E-208 | 1.98E-206 | 467.3241 | DOWN |
| LINC00885 | -8.32005 | -4.79428 | -44.9927 | 1.87E-208 | 3.51E-206 | 466.7286 | DOWN |
| U47924.27 | -11.3565 | -4.32277 | -44.3128 | 4.93E-205 | 9.00E-203 | 458.8588 | DOWN |
| LINC00675 | -7.87709 | -5.75558 | -44.2614 | 8.97E-205 | 1.60E-202 | 458.2612 | DOWN |
| MALAT1 | -4.2148 | 5.82072 | -44.081 | 7.32E-204 | 1.28E-201 | 456.1627 | DOWN |
| RP11-700H6.4 | -8.25319 | -6.28994 | -44.0189 | 1.51E-203 | 2.58E-201 | 455.4383 | DOWN |
| AF064858.11 | -8.60416 | -5.70078 | -43.9746 | 2.53E-203 | 4.22E-201 | 454.9226 | DOWN |
| CH17-360D5.1 | -6.98748 | -7.17237 | -43.6823 | 7.71E-202 | 1.26E-199 | 451.5094 | DOWN |
| LINC00592 | -8.10981 | -5.61487 | -43.2506 | 1.22E-199 | 1.95E-197 | 446.4482 | DOWN |
| CH507-42P11.7 | -9.51159 | -4.67031 | -43.1826 | 2.72E-199 | 4.25E-197 | 445.6484 | DOWN |
| LINC01290 | -7.20748 | -7.14317 | -42.8264 | 1.81E-197 | 2.77E-195 | 441.452 | DOWN |
| RP11-379F12.3 | -8.53213 | -5.65297 | -42.8218 | 1.91E-197 | 2.87E-195 | 441.3978 | DOWN |
| RP11-99A1.2 | -6.01301 | -7.81979 | -42.7299 | 5.66E-197 | 8.34E-195 | 440.3131 | DOWN |
| RP11-21B23.2 | -8.03055 | -6.76041 | -42.1303 | 6.93E-194 | 9.84E-192 | 433.2061 | DOWN |
| FAM95C | -9.13492 | -3.10693 | -42.0247 | 2.44E-193 | 3.40E-191 | 431.9498 | DOWN |
| SOX21-AS1 | -8.32377 | -3.93984 | -41.9048 | 1.02E-192 | 1.39E-190 | 430.5222 | DOWN |
| CTB-60B18.18 | -9.18449 | -4.47531 | -41.759 | 5.79E-192 | 7.79E-190 | 428.7833 | DOWN |
| RP11-35J10.5 | -7.43064 | -7.39048 | -41.3904 | 4.76E-190 | 6.30E-188 | 424.3763 | DOWN |
| AC004791.2 | -8.9478 | -5.76928 | -41.2518 | 2.51E-189 | 3.21E-187 | 422.7144 | DOWN |
| LINC01166 | -5.28661 | -7.90943 | -40.8893 | 1.96E-187 | 2.43E-185 | 418.3577 | DOWN |
| FAM83H-AS1 | -4.3219 | 1.774054 | -40.4976 | 2.22E-185 | 2.71E-183 | 413.6312 | DOWN |
| RP11-7K24.3 | -7.73022 | -2.52501 | -40.4952 | 2.29E-185 | 2.74E-183 | 413.6024 | DOWN |
| CTC-479C5.10 | -2.76078 | -0.72713 | -39.7093 | 3.19E-181 | 3.77E-179 | 404.0643 | DOWN |
| RP11-411K7.1 | -9.2204 | -3.27635 | -39.6353 | 7.87E-181 | 9.14E-179 | 403.1628 | DOWN |
| LINC01451 | -7.16939 | -2.94745 | -39.5528 | 2.16E-180 | 2.47E-178 | 402.1562 | DOWN |
| RP11-451G4.2 | -6.47171 | -7.34529 | -38.9106 | 5.62E-177 | 6.33E-175 | 394.295 | DOWN |
| LINC01305 | -7.12656 | -5.06295 | -38.8075 | 1.99E-176 | 2.21E-174 | 393.0291 | DOWN |
| RP11-680C21.1 | -6.17798 | -6.01896 | -38.5278 | 6.24E-175 | 6.74E-173 | 389.5871 | DOWN |
| RP11-462G2.1 | -9.89539 | -4.40239 | -37.9014 | 1.44E-171 | 1.54E-169 | 381.8454 | DOWN |
| C10orf95 | -4.68981 | -2.24 | -37.7191 | 1.39E-170 | 1.45E-168 | 379.5846 | DOWN |
| RP4-568C11.4 | -8.25974 | -2.58587 | -37.7119 | 1.52E-170 | 1.57E-168 | 379.4949 | DOWN |
| CTD-2515H24.2 | -6.59533 | -6.15173 | -37.3387 | 1.57E-168 | 1.59E-166 | 374.8539 | DOWN |
| LINC01133 | -8.43132 | -2.4555 | -36.92 | 2.94E-166 | 2.89E-164 | 369.6281 | DOWN |
| KB-1410C5.3 | -6.48729 | -7.47483 | -36.6881 | 5.36E-165 | 5.20E-163 | 366.7256 | DOWN |
| DIO3OS | -7.46807 | -1.0416 | -36.5011 | 5.60E-164 | 5.37E-162 | 364.3803 | DOWN |
| RP11-231E6.1 | -5.21428 | -7.91875 | -36.2983 | 7.16E-163 | 6.69E-161 | 361.8334 | DOWN |
| CTD-2619J13.13 | -10.867 | -2.28515 | -36.2121 | 2.12E-162 | 1.96E-160 | 360.7492 | DOWN |
| RP11-394O4.5 | -4.62073 | 0.014036 | -36.183 | 3.06E-162 | 2.79E-160 | 360.383 | DOWN |
| TINCR | -6.42887 | 1.112062 | -35.7484 | 7.34E-160 | 6.62E-158 | 354.9038 | DOWN |
| LINC00302 | -10.095 | -5.6628 | -35.3738 | 8.41E-158 | 7.50E-156 | 350.1658 | DOWN |
| RP11-677O4.6 | -9.80281 | -4.99307 | -35.3139 | 1.80E-157 | 1.58E-155 | 349.4069 | DOWN |
| MEG3 | -5.3945 | 1.990055 | -35.2282 | 5.33E-157 | 4.64E-155 | 348.3201 | DOWN |
| RP11-305L7.7 | -4.99998 | -4.65269 | -35.1423 | 1.59E-156 | 1.37E-154 | 347.2295 | DOWN |
| RP11-308D13.3 | -6.34513 | -6.89477 | -35.0899 | 3.09E-156 | 2.63E-154 | 346.5643 | DOWN |
| RP11-127L20.3 | -6.6498 | -6.07812 | -35.022 | 7.31E-156 | 6.16E-154 | 345.703 | DOWN |
| AF064858.8 | -6.03807 | -3.54955 | -35.0105 | 8.46E-156 | 7.05E-154 | 345.5568 | DOWN |
| SCGB1B2P | -6.89996 | 0.735797 | -34.9622 | 1.56E-155 | 1.29E-153 | 344.9425 | DOWN |
| AC087491.2 | -7.49719 | -5.55117 | -34.96 | 1.61E-155 | 1.31E-153 | 344.9148 | DOWN |
| RP11-129I19.2 | -6.11627 | -7.55225 | -34.8119 | 1.06E-154 | 8.55E-153 | 343.0306 | DOWN |
| RP11-582E3.6 | -2.28932 | 1.66858 | -34.6495 | 8.38E-154 | 6.69E-152 | 340.9636 | DOWN |
| C3orf35 | -3.65566 | -1.0034 | -34.6426 | 9.15E-154 | 7.23E-152 | 340.8758 | DOWN |
| RP11-1275H24.1 | -3.66252 | -2.14473 | -34.4662 | 8.69E-153 | 6.79E-151 | 338.6265 | DOWN |
| LINC00640 | -8.11477 | -2.43882 | -34.4586 | 9.57E-153 | 7.41E-151 | 338.5299 | DOWN |
| AC007193.6 | -8.26492 | -5.77136 | -34.2881 | 8.45E-152 | 6.48E-150 | 336.353 | DOWN |
| RP11-211G3.2 | -7.85968 | -4.32185 | -33.6799 | 2.05E-148 | 1.52E-146 | 328.5652 | DOWN |
| RP11-321G12.1 | -7.21439 | -5.01118 | -33.5037 | 1.97E-147 | 1.45E-145 | 326.3026 | DOWN |
| RP1-288H2.2 | -5.77404 | -8.0001 | -33.4282 | 5.19E-147 | 3.79E-145 | 325.3325 | DOWN |
| CTD-3032H12.1 | -5.95789 | -7.67658 | -33.2174 | 7.84E-146 | 5.62E-144 | 322.6195 | DOWN |
| CH507-42P11.6 | -6.75717 | -5.52488 | -33.1997 | 9.84E-146 | 6.98E-144 | 322.3926 | DOWN |
| LINC00969 | -2.25754 | 4.477505 | -33.1826 | 1.23E-145 | 8.62E-144 | 322.1726 | DOWN |
| RP11-681H18.2 | -2.18761 | 3.8119 | -33.1388 | 2.16E-145 | 1.49E-143 | 321.6083 | DOWN |
| MIR143HG | -4.45899 | -1.47858 | -33.0864 | 4.24E-145 | 2.90E-143 | 320.9331 | DOWN |
| RP1-159G19.1 | -6.66672 | -7.14605 | -33.0392 | 7.79E-145 | 5.29E-143 | 320.3243 | DOWN |
| LINC01554 | -5.79783 | -2.01859 | -32.9545 | 2.32E-144 | 1.56E-142 | 319.2324 | DOWN |
| MIR210HG | -3.52207 | 3.051605 | -32.8591 | 7.96E-144 | 5.31E-142 | 318.0013 | DOWN |
| LINC00992 | -7.34565 | -5.41198 | -32.8374 | 1.05E-143 | 6.96E-142 | 317.7222 | DOWN |
| AP001626.1 | -6.05004 | -6.21136 | -32.8044 | 1.61E-143 | 1.06E-141 | 317.2963 | DOWN |
| RP11-88H9.2 | -6.28477 | -4.85587 | -32.7434 | 3.55E-143 | 2.30E-141 | 316.5083 | DOWN |
| RP11-774O3.3 | -2.95626 | 0.01291 | -32.7205 | 4.77E-143 | 3.07E-141 | 316.212 | DOWN |
| AC144831.1 | -3.5841 | -0.46691 | -32.7132 | 5.24E-143 | 3.35E-141 | 316.118 | DOWN |
| AC138647.1 | -6.11951 | -7.87787 | -32.5874 | 2.67E-142 | 1.68E-140 | 314.4919 | DOWN |
| RP11-167B3.2 | -6.8911 | -6.3815 | -32.5433 | 4.72E-142 | 2.94E-140 | 313.9221 | DOWN |
| C1orf132 | -4.59097 | -3.15467 | -32.5129 | 6.99E-142 | 4.32E-140 | 313.529 | DOWN |
| AC008268.1 | -6.3906 | -7.19301 | -32.3775 | 4.03E-141 | 2.47E-139 | 311.7776 | DOWN |
| LINC01219 | -6.70554 | -6.29857 | -32.3236 | 8.11E-141 | 4.93E-139 | 311.0791 | DOWN |
| RP11-1263C18.1 | -7.15881 | -6.68104 | -32.1715 | 5.83E-140 | 3.52E-138 | 309.1082 | DOWN |
| XXbac-BPG27H4.8 | -6.04753 | -7.29546 | -32.0443 | 3.03E-139 | 1.82E-137 | 307.4596 | DOWN |
| KB-1930G5.4 | -5.47159 | -8.03996 | -31.9056 | 1.84E-138 | 1.08E-136 | 305.6592 | DOWN |
| RP11-480C16.1 | -4.95186 | -6.4505 | -31.7621 | 1.19E-137 | 6.94E-136 | 303.7956 | DOWN |
| RP11-731D1.4 | -6.52666 | -6.59972 | -31.7534 | 1.33E-137 | 7.71E-136 | 303.6823 | DOWN |
| RP11-497G19.7 | -4.89948 | -7.03737 | -31.6171 | 7.81E-137 | 4.47E-135 | 301.9113 | DOWN |
| LINC00102 | -5.38469 | -7.02343 | -31.4963 | 3.76E-136 | 2.12E-134 | 300.3397 | DOWN |
| LINC01176 | -3.82049 | 0.53979 | -30.9958 | 2.57E-133 | 1.41E-131 | 293.8188 | DOWN |
| LINC01503 | -2.65023 | 1.81614 | -30.9199 | 6.92E-133 | 3.76E-131 | 292.8274 | DOWN |
| CYP4F26P | -7.49148 | -5.03388 | -30.8499 | 1.73E-132 | 9.32E-131 | 291.9143 | DOWN |
| RP11-804H8.6 | -3.66071 | -0.1892 | -30.6878 | 1.44E-131 | 7.64E-130 | 289.7971 | DOWN |
| LINC01343 | -5.44143 | -6.35465 | -30.6084 | 4.06E-131 | 2.13E-129 | 288.7589 | DOWN |
| AP000473.8 | -6.23867 | -5.08166 | -30.5249 | 1.21E-130 | 6.31E-129 | 287.6667 | DOWN |
| RP1-80N2.2 | -6.75884 | -5.46459 | -30.2083 | 7.64E-129 | 3.88E-127 | 283.524 | DOWN |
| NEAT1 | -3.1693 | 8.397369 | -30.1476 | 1.69E-128 | 8.54E-127 | 282.7283 | DOWN |
| RP11-44F21.5 | -7.82466 | -4.56061 | -30.1132 | 2.66E-128 | 1.33E-126 | 282.278 | DOWN |
| RP11-1275H24.3 | -3.09195 | -0.62561 | -30.0849 | 3.85E-128 | 1.92E-126 | 281.9067 | DOWN |
| RP11-863P13.5 | -6.51018 | -6.4069 | -29.6996 | 6.05E-126 | 2.97E-124 | 276.8537 | DOWN |
| AC005042.5 | -5.64522 | -7.89803 | -29.5931 | 2.45E-125 | 1.20E-123 | 275.4555 | DOWN |
| LINC00202-1 | -4.99338 | -2.267 | -29.5482 | 4.42E-125 | 2.15E-123 | 274.8657 | DOWN |
| CTA-276F8.1 | -3.7342 | -2.31105 | -29.4411 | 1.81E-124 | 8.72E-123 | 273.4588 | DOWN |
| RP11-481J2.2 | -7.20808 | -3.51661 | -29.418 | 2.45E-124 | 1.17E-122 | 273.1557 | DOWN |
| RP11-367F23.2 | -6.70476 | -5.01201 | -29.3759 | 4.26E-124 | 2.01E-122 | 272.6026 | DOWN |
| CTD-3252C9.4 | -2.72711 | 3.259816 | -29.284 | 1.43E-123 | 6.66E-122 | 271.3949 | DOWN |
| RP11-367G18.1 | -7.38403 | -2.2383 | -29.2717 | 1.68E-123 | 7.79E-122 | 271.2335 | DOWN |
| RP11-311F12.1 | -4.82038 | -5.72998 | -29.254 | 2.11E-123 | 9.76E-122 | 271.0011 | DOWN |
| CTC-321K16.4 | -4.09358 | -8.42771 | -29.1552 | 7.77E-123 | 3.54E-121 | 269.7008 | DOWN |
| LINC00989 | -4.9184 | -4.66072 | -29.0989 | 1.63E-122 | 7.39E-121 | 268.9606 | DOWN |
| RP11-506F3.1 | -7.17205 | -4.16316 | -29.0759 | 2.20E-122 | 9.94E-121 | 268.658 | DOWN |
| CTC-281F24.1 | -5.57458 | -2.90276 | -29.0684 | 2.43E-122 | 1.09E-120 | 268.5603 | DOWN |
| LINC01552 | -6.32151 | -6.27019 | -29.0372 | 3.67E-122 | 1.64E-120 | 268.1492 | DOWN |
| AP001187.9 | -4.4259 | -6.32604 | -28.9667 | 9.28E-122 | 4.09E-120 | 267.2213 | DOWN |
| RP11-255H23.4 | -5.64353 | -6.1925 | -28.867 | 3.45E-121 | 1.51E-119 | 265.909 | DOWN |
| RP3-522J7.6 | -4.95256 | -5.86964 | -28.8486 | 4.39E-121 | 1.91E-119 | 265.6678 | DOWN |
| RP11-336A10.4 | -6.59048 | -6.54265 | -28.7909 | 9.41E-121 | 4.05E-119 | 264.907 | DOWN |
| LINC01550 | -5.36767 | -0.60506 | -28.771 | 1.22E-120 | 5.24E-119 | 264.6454 | DOWN |
| AC007381.3 | -5.45815 | -5.83839 | -28.7497 | 1.62E-120 | 6.85E-119 | 264.3655 | DOWN |
| RP11-610P16.1 | -6.34861 | -7.12022 | -28.63 | 7.84E-120 | 3.30E-118 | 262.788 | DOWN |
| RP11-219B17.3 | -7.15874 | -3.9329 | -28.5623 | 1.91E-119 | 7.98E-118 | 261.8963 | DOWN |
| C20orf203 | -4.71154 | -5.555 | -28.5599 | 1.97E-119 | 8.18E-118 | 261.8656 | DOWN |
| TUSC8 | -6.52252 | -6.75662 | -28.5419 | 2.50E-119 | 1.03E-117 | 261.6276 | DOWN |
| LINC00162 | -7.09758 | -4.75539 | -28.4692 | 6.53E-119 | 2.68E-117 | 260.6703 | DOWN |
| LINC00599 | -4.53198 | -7.91758 | -28.1854 | 2.76E-117 | 1.13E-115 | 256.9286 | DOWN |
| RP11-231E19.1 | -4.47142 | -7.07537 | -28.0483 | 1.69E-116 | 6.84E-115 | 255.1204 | DOWN |
| RP1-7G5.6 | -5.67179 | -6.87557 | -28.03 | 2.15E-116 | 8.67E-115 | 254.8785 | DOWN |
| RP11-390P24.1 | -2.12938 | 2.171054 | -27.9219 | 8.94E-116 | 3.57E-114 | 253.4529 | DOWN |
| AC008440.10 | -6.75268 | -5.89287 | -27.9038 | 1.14E-115 | 4.52E-114 | 253.2131 | DOWN |
| LINC01229 | -6.58597 | -3.35789 | -27.831 | 2.97E-115 | 1.17E-113 | 252.2534 | DOWN |
| AP000688.29 | -5.10771 | -6.24203 | -27.808 | 4.03E-115 | 1.58E-113 | 251.9486 | DOWN |
| AC019064.1 | -5.34979 | -8.13805 | -27.7556 | 8.05E-115 | 3.15E-113 | 251.2571 | DOWN |
| NHEG1 | -6.09649 | -6.52195 | -27.7237 | 1.23E-114 | 4.77E-113 | 250.8366 | DOWN |
| RP11-416I2.1 | -4.35704 | -7.44526 | -27.5842 | 7.74E-114 | 2.98E-112 | 248.9954 | DOWN |
| CTC-497E21.3 | -6.51429 | -5.61332 | -27.3677 | 1.35E-112 | 5.14E-111 | 246.1353 | DOWN |
| RP11-379F12.4 | -5.98276 | -7.55012 | -27.3597 | 1.50E-112 | 5.68E-111 | 246.0305 | DOWN |
| LINC00908 | -5.86948 | -4.73995 | -27.2873 | 3.92E-112 | 1.46E-110 | 245.0743 | DOWN |
| LINC00958 | -7.44366 | -3.11763 | -27.2871 | 3.93E-112 | 1.46E-110 | 245.0714 | DOWN |
| RP11-689P11.2 | -3.87353 | -2.73884 | -27.254 | 6.08E-112 | 2.25E-110 | 244.6342 | DOWN |
| LINC01544 | -2.95611 | -8.90619 | -27.2388 | 7.44E-112 | 2.74E-110 | 244.4335 | DOWN |
| RP5-1097P24.1 | -5.43368 | -8.04518 | -27.1546 | 2.26E-111 | 8.27E-110 | 243.3213 | DOWN |
| AC003090.1 | -5.90044 | -2.51904 | -27.1312 | 3.09E-111 | 1.12E-109 | 243.0116 | DOWN |
| CTD-2306A12.1 | -6.70387 | -4.48908 | -27.1114 | 4.01E-111 | 1.45E-109 | 242.7498 | DOWN |
| AC004471.10 | -4.30771 | -3.6419 | -27.104 | 4.42E-111 | 1.59E-109 | 242.652 | DOWN |
| RP11-689K5.3 | -7.42052 | -4.79275 | -27.0736 | 6.61E-111 | 2.37E-109 | 242.2512 | DOWN |
| RP3-406A7.7 | -7.33727 | -1.50599 | -27.0331 | 1.13E-110 | 4.03E-109 | 241.7156 | DOWN |
| AC006273.5 | -5.95913 | -4.21958 | -26.9846 | 2.14E-110 | 7.57E-109 | 241.0751 | DOWN |
| AC067959.1 | -6.42822 | -6.29099 | -26.8866 | 7.84E-110 | 2.76E-108 | 239.7795 | DOWN |
| RP11-863P13.4 | -4.64492 | -4.23663 | -26.8356 | 1.54E-109 | 5.36E-108 | 239.1058 | DOWN |
| CTD-2184D3.3 | -5.65756 | -7.66933 | -26.8342 | 1.57E-109 | 5.44E-108 | 239.0869 | DOWN |
| C12orf80 | -4.9144 | -7.93787 | -26.8143 | 2.04E-109 | 7.04E-108 | 238.8243 | DOWN |
| RP11-159D12.2 | -2.82868 | 1.763208 | -26.8085 | 2.20E-109 | 7.57E-108 | 238.7473 | DOWN |
| CTA-280A3.2 | -4.84053 | -5.82302 | -26.7008 | 9.15E-109 | 3.12E-107 | 237.3246 | DOWN |
| CTA-228A9.3 | -3.83806 | -0.64537 | -26.556 | 6.21E-108 | 2.07E-106 | 235.4102 | DOWN |
| LINC01068 | -6.4616 | -6.12776 | -26.5157 | 1.06E-107 | 3.50E-106 | 234.8775 | DOWN |
| LINC01422 | -4.2679 | -4.56354 | -26.4574 | 2.29E-107 | 7.53E-106 | 234.108 | DOWN |
| AP000473.5 | -6.615 | -4.49653 | -26.4464 | 2.65E-107 | 8.67E-106 | 233.9628 | DOWN |
| CTD-2523D13.1 | -5.03133 | -8.25115 | -26.4397 | 2.89E-107 | 9.43E-106 | 233.8741 | DOWN |
| LINC01018 | -5.15448 | -5.18533 | -26.4327 | 3.17E-107 | 1.03E-105 | 233.7816 | DOWN |
| AC138035.2 | -2.55677 | 1.707479 | -26.428 | 3.38E-107 | 1.09E-105 | 233.7184 | DOWN |
| AC016735.2 | -6.68954 | -4.1737 | -26.4131 | 4.11E-107 | 1.32E-105 | 233.522 | DOWN |
| RP11-367H1.1 | -5.54807 | -4.36564 | -26.3433 | 1.03E-106 | 3.32E-105 | 232.6002 | DOWN |
| RP11-373D23.3 | -4.28468 | -1.54751 | -26.2838 | 2.27E-106 | 7.23E-105 | 231.8134 | DOWN |
| RP11-293M10.1 | -5.76374 | -6.56553 | -26.2755 | 2.54E-106 | 8.04E-105 | 231.7032 | DOWN |
| CTD-2600O9.1 | -5.59812 | -5.39445 | -26.2445 | 3.82E-106 | 1.21E-104 | 231.2945 | DOWN |
| PART1 | -5.63386 | -3.69335 | -26.1098 | 2.27E-105 | 7.08E-104 | 229.5135 | DOWN |
| RP11-380J14.1 | -4.43532 | -7.10117 | -26.0881 | 3.03E-105 | 9.40E-104 | 229.2265 | DOWN |
| AC008592.8 | -6.60024 | -4.16224 | -26.0768 | 3.52E-105 | 1.09E-103 | 229.0769 | DOWN |
| RP4-563E14.1 | -2.20429 | 0.789616 | -25.9992 | 9.81E-105 | 3.00E-103 | 228.0515 | DOWN |
| FOXCUT | -7.15331 | -5.61003 | -25.9359 | 2.26E-104 | 6.89E-103 | 227.216 | DOWN |
| FAM157A | -5.32178 | -6.98509 | -25.9091 | 3.23E-104 | 9.75E-103 | 226.861 | DOWN |
| RP5-902P8.12 | -3.05239 | -2.97589 | -25.8418 | 7.87E-104 | 2.35E-102 | 225.972 | DOWN |
| CTD-2135D7.5 | -5.89232 | -5.21396 | -25.8365 | 8.44E-104 | 2.51E-102 | 225.9022 | DOWN |
| CTD-2015G9.2 | -5.64343 | -4.62495 | -25.8236 | 1.00E-103 | 2.96E-102 | 225.7315 | DOWN |
| RP11-403A21.1 | -5.22523 | -5.92578 | -25.764 | 2.20E-103 | 6.47E-102 | 224.9439 | DOWN |
| RP11-258C19.7 | -2.25372 | 1.894303 | -25.6987 | 5.22E-103 | 1.53E-101 | 224.082 | DOWN |
| LINC00672 | -3.14118 | -2.08662 | -25.6526 | 9.61E-103 | 2.80E-101 | 223.4718 | DOWN |
| RP11-574K11.29 | -2.45301 | 0.097769 | -25.6012 | 1.90E-102 | 5.50E-101 | 222.7928 | DOWN |
| RP11-855O10.2 | -3.91173 | -8.02907 | -25.5183 | 5.67E-102 | 1.63E-100 | 221.6979 | DOWN |
| RP11-95O2.1 | -4.49376 | -6.10748 | -25.4174 | 2.15E-101 | 6.18E-100 | 220.3661 | DOWN |
| RP11-74E22.8 | -5.25675 | -3.72842 | -25.4152 | 2.21E-101 | 6.33E-100 | 220.3371 | DOWN |
| RP11-54O7.17 | -3.87234 | -1.09338 | -25.4065 | 2.49E-101 | 7.08E-100 | 220.2216 | DOWN |
| AF064858.10 | -6.36399 | -7.17617 | -25.3911 | 3.05E-101 | 8.65E-100 | 220.0184 | DOWN |
| RP11-79N23.1 | -5.71216 | -3.99028 | -25.3519 | 5.11E-101 | 1.45E-99 | 219.5009 | DOWN |
| RP11-736N17.10 | -6.0907 | -4.4774 | -25.2678 | 1.55E-100 | 4.35E-99 | 218.3899 | DOWN |
| FAM223A | -3.08667 | -2.19513 | -25.2477 | 2.03E-100 | 5.63E-99 | 218.1251 | DOWN |
| RP11-206L10.3 | -3.34289 | -1.09371 | -25.1286 | 9.78E-100 | 2.71E-98 | 216.552 | DOWN |
| RP11-3N13.2 | -4.70504 | -8.26394 | -25.0075 | 4.84E-99 | 1.34E-97 | 214.9544 | DOWN |
| AC093159.1 | -6.76919 | -4.05202 | -24.9822 | 6.77E-99 | 1.86E-97 | 214.6198 | DOWN |
| RP11-410E4.1 | -3.38001 | -3.52988 | -24.9283 | 1.38E-98 | 3.78E-97 | 213.9088 | DOWN |
| RP11-276H19.2 | -4.61871 | -4.91306 | -24.9001 | 2.00E-98 | 5.44E-97 | 213.5367 | DOWN |
| LINC01208 | -5.69868 | -7.10486 | -24.8608 | 3.36E-98 | 9.10E-97 | 213.0187 | DOWN |
| FLJ22447 | -2.78687 | -2.08321 | -24.7875 | 8.86E-98 | 2.37E-96 | 212.0508 | DOWN |
| AC115522.3 | -6.1672 | -2.84633 | -24.7009 | 2.78E-97 | 7.39E-96 | 210.9095 | DOWN |
| RP1-193H18.3 | -6.44605 | -6.6496 | -24.65 | 5.44E-97 | 1.44E-95 | 210.2374 | DOWN |
| RP11-678G14.3 | -5.12141 | -6.76142 | -24.5906 | 1.19E-96 | 3.13E-95 | 209.4546 | DOWN |
| RP11-212I21.4 | -3.53841 | -3.05809 | -24.5493 | 2.05E-96 | 5.35E-95 | 208.9109 | DOWN |
| RP11-817J15.3 | -5.25052 | -7.25862 | -24.4455 | 8.08E-96 | 2.08E-94 | 207.5417 | DOWN |
| RP3-340B19.3 | -5.14367 | -7.6829 | -24.3661 | 2.30E-95 | 5.88E-94 | 206.4959 | DOWN |
| CTD-2515H24.4 | -4.70103 | -7.88389 | -24.3562 | 2.62E-95 | 6.68E-94 | 206.366 | DOWN |
| RP11-396B14.2 | -4.97452 | -5.51074 | -24.3459 | 3.00E-95 | 7.62E-94 | 206.2302 | DOWN |
| RP5-884C9.2 | -5.92681 | -1.87393 | -24.3346 | 3.49E-95 | 8.83E-94 | 206.0807 | DOWN |
| RP4-669L17.10 | -2.00202 | 3.598422 | -24.3067 | 5.03E-95 | 1.27E-93 | 205.714 | DOWN |
| RP11-481J13.1 | -4.27907 | -7.82581 | -24.2833 | 6.86E-95 | 1.72E-93 | 205.405 | DOWN |
| RP11-408A13.4 | -3.04692 | -1.12456 | -24.1451 | 4.24E-94 | 1.06E-92 | 203.5859 | DOWN |
| CTC-505O3.3 | -4.8504 | -7.63339 | -24.0559 | 1.37E-93 | 3.41E-92 | 202.4118 | DOWN |
| LINC00670 | -4.11964 | -7.97641 | -23.999 | 2.91E-93 | 7.16E-92 | 201.6625 | DOWN |
| C20orf197 | -3.86919 | -4.57469 | -23.9698 | 4.27E-93 | 1.05E-91 | 201.2789 | DOWN |
| RP11-776A13.3 | -5.24745 | -7.92892 | -23.9682 | 4.36E-93 | 1.07E-91 | 201.258 | DOWN |
| RP11-867G23.3 | -2.00167 | -2.86573 | -23.9583 | 4.96E-93 | 1.21E-91 | 201.1278 | DOWN |
| LINC01127 | -4.27397 | -4.08303 | -23.9201 | 8.21E-93 | 1.99E-91 | 200.6254 | DOWN |
| RP11-250B2.5 | -3.90859 | -2.99069 | -23.9124 | 9.09E-93 | 2.20E-91 | 200.5232 | DOWN |
| RP11-347I19.7 | -2.90437 | -0.64083 | -23.8943 | 1.15E-92 | 2.78E-91 | 200.2853 | DOWN |
| RP11-2N1.3 | -4.9168 | -8.20725 | -23.8844 | 1.31E-92 | 3.16E-91 | 200.1549 | DOWN |
| LINC01089 | -2.00112 | 3.065667 | -23.8597 | 1.82E-92 | 4.36E-91 | 199.8309 | DOWN |
| MIR22HG | -2.00592 | 3.035621 | -23.7624 | 6.54E-92 | 1.56E-90 | 198.5515 | DOWN |
| RP11-475A13.1 | -3.75448 | -8.49534 | -23.7492 | 7.78E-92 | 1.85E-90 | 198.3788 | DOWN |
| AC093609.1 | -4.5363 | -3.78519 | -23.7002 | 1.48E-91 | 3.52E-90 | 197.7336 | DOWN |
| LINC00222 | -4.54776 | -5.72451 | -23.6958 | 1.57E-91 | 3.72E-90 | 197.6765 | DOWN |
| AC007563.1 | -2.92383 | -8.8652 | -23.6511 | 2.83E-91 | 6.65E-90 | 197.0892 | DOWN |
| RP11-672A2.1 | -4.61697 | -8.04279 | -23.6493 | 2.90E-91 | 6.80E-90 | 197.065 | DOWN |
| RP1-78B3.1 | -3.34302 | -6.10522 | -23.5977 | 5.71E-91 | 1.34E-89 | 196.3874 | DOWN |
| LINC01214 | -6.66483 | -6.57148 | -23.5867 | 6.60E-91 | 1.54E-89 | 196.2436 | DOWN |
| RP11-180N14.1 | -4.46301 | -3.63292 | -23.5804 | 7.17E-91 | 1.67E-89 | 196.1604 | DOWN |
| RP11-286B14.1 | -5.5341 | -5.65584 | -23.5021 | 2.01E-90 | 4.65E-89 | 195.1323 | DOWN |
| RP11-324E6.6 | -5.53292 | -6.43896 | -23.4915 | 2.31E-90 | 5.33E-89 | 194.9926 | DOWN |
| LINC00950 | -2.95605 | -2.62425 | -23.4379 | 4.66E-90 | 1.07E-88 | 194.2898 | DOWN |
| RP11-420A23.1 | -2.82095 | 0.073388 | -23.3214 | 2.15E-89 | 4.95E-88 | 192.7611 | DOWN |
| FLJ33360 | -4.92191 | -4.31913 | -23.3102 | 2.50E-89 | 5.71E-88 | 192.6144 | DOWN |
| LINC00968 | -4.47632 | -3.46473 | -23.2789 | 3.77E-89 | 8.59E-88 | 192.2035 | DOWN |
| ERVH48-1 | -3.72008 | -5.78313 | -23.2148 | 8.73E-89 | 1.99E-87 | 191.3632 | DOWN |
| RP11-1134I14.8 | -4.16089 | -5.4453 | -23.1892 | 1.22E-88 | 2.77E-87 | 191.0279 | DOWN |
| LINC00964 | -6.07504 | -3.04169 | -23.1891 | 1.22E-88 | 2.77E-87 | 191.0263 | DOWN |
| RP11-798L4.1 | -3.59294 | -8.50799 | -23.1547 | 1.92E-88 | 4.33E-87 | 190.5763 | DOWN |
| XXbac-BPGBPG55C20.2 | -2.95 | -0.53078 | -23.1435 | 2.22E-88 | 5.00E-87 | 190.4294 | DOWN |
| RP11-563J2.3 | -3.38247 | -3.66288 | -22.9808 | 1.88E-87 | 4.20E-86 | 188.2981 | DOWN |
| AL022344.7 | -4.96527 | -5.75745 | -22.9322 | 3.55E-87 | 7.90E-86 | 187.6615 | DOWN |
| RP11-434B12.1 | -2.03404 | -0.31515 | -22.9277 | 3.77E-87 | 8.35E-86 | 187.6028 | DOWN |
| LINC01395 | -4.11531 | -8.12956 | -22.8854 | 6.55E-87 | 1.45E-85 | 187.0502 | DOWN |
| RP11-295M3.4 | -3.55206 | -0.29072 | -22.8792 | 7.11E-87 | 1.57E-85 | 186.9682 | DOWN |
| RP3-429O6.1 | -4.88251 | -8.17271 | -22.8472 | 1.08E-86 | 2.37E-85 | 186.5502 | DOWN |
| LINC00342 | -2.67875 | 1.56186 | -22.8388 | 1.21E-86 | 2.64E-85 | 186.4401 | DOWN |
| RP11-64C1.1 | -4.42191 | -8.05326 | -22.8357 | 1.26E-86 | 2.74E-85 | 186.3991 | DOWN |
| RP11-543C4.1 | -3.86963 | -2.93079 | -22.8212 | 1.52E-86 | 3.30E-85 | 186.2094 | DOWN |
| LINC00163 | -4.47019 | -5.75866 | -22.8158 | 1.63E-86 | 3.53E-85 | 186.1396 | DOWN |
| FLJ12825 | -3.06795 | -3.47648 | -22.7986 | 2.04E-86 | 4.41E-85 | 185.915 | DOWN |
| RP11-102K13.5 | -3.90286 | -4.28232 | -22.7673 | 3.08E-86 | 6.63E-85 | 185.5054 | DOWN |
| RP11-285E9.6 | -5.17406 | -6.6066 | -22.747 | 4.02E-86 | 8.62E-85 | 185.2397 | DOWN |
| RP11-354A14.1 | -5.5053 | -6.5637 | -22.7306 | 4.97E-86 | 1.07E-84 | 185.026 | DOWN |
| RP1-97J1.2 | -4.81869 | -8.18209 | -22.7086 | 6.64E-86 | 1.41E-84 | 184.7374 | DOWN |
| AC068831.16 | -5.68952 | -2.83158 | -22.6961 | 7.82E-86 | 1.66E-84 | 184.5741 | DOWN |
| AC002511.2 | -5.26454 | -7.33718 | -22.6785 | 9.84E-86 | 2.08E-84 | 184.3448 | DOWN |
| GAS6-AS2 | -2.85026 | -1.81177 | -22.5819 | 3.48E-85 | 7.29E-84 | 183.0826 | DOWN |
| PGM5P2 | -3.3879 | -3.47811 | -22.5618 | 4.53E-85 | 9.46E-84 | 182.8201 | DOWN |
| PCAT19 | -2.01865 | -0.34579 | -22.5483 | 5.40E-85 | 1.12E-83 | 182.6442 | DOWN |
| LINC01267 | -3.97017 | -7.37401 | -22.506 | 9.38E-85 | 1.95E-83 | 182.0922 | DOWN |
| RP11-395I14.2 | -5.44417 | -2.99657 | -22.4792 | 1.33E-84 | 2.76E-83 | 181.7425 | DOWN |
| SNHG24 | -4.19508 | -7.41689 | -22.4459 | 2.06E-84 | 4.24E-83 | 181.3083 | DOWN |
| LINC01167 | -4.19375 | -8.39818 | -22.3814 | 4.78E-84 | 9.82E-83 | 180.4672 | DOWN |
| RP4-614O4.12 | -3.41313 | -2.40124 | -22.3574 | 6.53E-84 | 1.34E-82 | 180.1539 | DOWN |
| RP11-95I16.6 | -4.12718 | -8.43817 | -22.3032 | 1.33E-83 | 2.69E-82 | 179.4476 | DOWN |
| ADAM20P1 | -3.00988 | -3.58135 | -22.2624 | 2.26E-83 | 4.54E-82 | 178.9155 | DOWN |
| RP6-201G10.2 | -2.90355 | -1.47639 | -22.247 | 2.76E-83 | 5.54E-82 | 178.7153 | DOWN |
| LINC01106 | -3.78062 | -0.41192 | -22.2028 | 4.91E-83 | 9.82E-82 | 178.1402 | DOWN |
| RP11-20D14.6 | -4.16101 | -1.77401 | -22.1819 | 6.44E-83 | 1.28E-81 | 177.8681 | DOWN |
| RP4-813D12.3 | -4.82236 | -6.33852 | -22.142 | 1.08E-82 | 2.15E-81 | 177.3489 | DOWN |
| RP11-348P10.2 | -2.48914 | -0.87809 | -22.1042 | 1.77E-82 | 3.51E-81 | 176.8564 | DOWN |
| RP13-631K18.3 | -4.59989 | -7.35418 | -22.0446 | 3.86E-82 | 7.57E-81 | 176.0807 | DOWN |
| RP11-543D5.2 | -6.13326 | -5.54733 | -22.0191 | 5.37E-82 | 1.05E-80 | 175.75 | DOWN |
| AC005537.2 | -5.69133 | -4.53117 | -22.013 | 5.82E-82 | 1.14E-80 | 175.6703 | DOWN |
| AC139099.4 | -4.99296 | -7.35837 | -21.9389 | 1.53E-81 | 2.95E-80 | 174.7076 | DOWN |
| RP11-731K22.1 | -3.45924 | -8.25525 | -21.9296 | 1.72E-81 | 3.33E-80 | 174.5863 | DOWN |
| CTD-3032H12.2 | -5.76967 | -6.47304 | -21.9008 | 2.51E-81 | 4.82E-80 | 174.2121 | DOWN |
| LINC01310 | -3.73464 | -7.85104 | -21.8741 | 3.54E-81 | 6.79E-80 | 173.866 | DOWN |
| RP11-276H19.1 | -2.54739 | -2.74415 | -21.8639 | 4.05E-81 | 7.74E-80 | 173.7331 | DOWN |
| RP11-707A18.1 | -4.99892 | -5.80217 | -21.8599 | 4.27E-81 | 8.13E-80 | 173.6812 | DOWN |
| RP11-324D17.1 | -5.02882 | -7.93701 | -21.8377 | 5.69E-81 | 1.08E-79 | 173.393 | DOWN |
| LINC00330 | -3.71689 | -8.1846 | -21.8062 | 8.57E-81 | 1.62E-79 | 172.9839 | DOWN |
| RP4-798A10.2 | -2.02067 | -1.71143 | -21.7284 | 2.35E-80 | 4.40E-79 | 171.975 | DOWN |
| RP11-567M16.2 | -5.4239 | -6.39136 | -21.7049 | 3.19E-80 | 5.95E-79 | 171.6703 | DOWN |
| RP11-245D16.4 | -2.05869 | 0.503705 | -21.6857 | 4.10E-80 | 7.61E-79 | 171.4213 | DOWN |
| RP11-13K12.2 | -3.61195 | -8.3065 | -21.5177 | 3.63E-79 | 6.69E-78 | 169.2439 | DOWN |
| MEG9 | -4.81377 | -4.54367 | -21.4999 | 4.57E-79 | 8.40E-78 | 169.014 | DOWN |
| RP11-728K20.2 | -3.2268 | -8.01859 | -21.4925 | 5.03E-79 | 9.20E-78 | 168.9181 | DOWN |
| CASC18 | -3.88123 | -6.32224 | -21.4925 | 5.03E-79 | 9.20E-78 | 168.9176 | DOWN |
| RP11-434H6.7 | -2.13808 | 1.194296 | -21.4564 | 8.02E-79 | 1.46E-77 | 168.4514 | DOWN |
| MLLT4-AS1 | -3.50132 | -2.48643 | -21.429 | 1.14E-78 | 2.08E-77 | 168.0969 | DOWN |
| RP11-834C11.11 | -3.50589 | -3.78061 | -21.4237 | 1.23E-78 | 2.22E-77 | 168.0287 | DOWN |
| RP11-79H23.3 | -3.82935 | -4.00929 | -21.3277 | 4.25E-78 | 7.62E-77 | 166.7876 | DOWN |
| RP11-378A12.1 | -3.14317 | -8.65336 | -21.2823 | 7.64E-78 | 1.37E-76 | 166.2009 | DOWN |
| RP1-93H18.1 | -4.57097 | -5.12214 | -21.2698 | 8.98E-78 | 1.61E-76 | 166.0394 | DOWN |
| RP11-2C24.7 | -4.66358 | -3.38062 | -21.2389 | 1.34E-77 | 2.39E-76 | 165.6401 | DOWN |
| RP1-122P22.4 | -3.49271 | -3.23919 | -21.2081 | 2.00E-77 | 3.55E-76 | 165.2422 | DOWN |
| RP11-13K12.1 | -4.16733 | -5.34298 | -21.1894 | 2.54E-77 | 4.51E-76 | 165.0015 | DOWN |
| RP1-278O22.1 | -5.82652 | -3.60922 | -21.1845 | 2.71E-77 | 4.79E-76 | 164.9376 | DOWN |
| LINC01224 | -4.3775 | -5.67128 | -21.1605 | 3.69E-77 | 6.50E-76 | 164.6283 | DOWN |
| RP11-259O2.2 | -4.07229 | -8.46532 | -21.0927 | 8.86E-77 | 1.55E-75 | 163.7537 | DOWN |
| RP11-54H7.4 | -4.40735 | -4.82978 | -21.0821 | 1.02E-76 | 1.78E-75 | 163.6174 | DOWN |
| AC093375.1 | -5.26266 | -4.88081 | -21.0278 | 2.05E-76 | 3.57E-75 | 162.9175 | DOWN |
| AC002511.3 | -4.14956 | -7.53205 | -21.0037 | 2.79E-76 | 4.86E-75 | 162.6069 | DOWN |
| LINC00319 | -4.5892 | -5.41349 | -20.9968 | 3.05E-76 | 5.28E-75 | 162.5186 | DOWN |
| RP11-167N5.5 | -4.90998 | -3.11397 | -20.9941 | 3.16E-76 | 5.46E-75 | 162.4833 | DOWN |
| CTD-2587M2.1 | -3.19032 | -4.04573 | -20.9854 | 3.53E-76 | 6.09E-75 | 162.3722 | DOWN |
| AF064858.7 | -3.61697 | -8.08436 | -20.9524 | 5.41E-76 | 9.28E-75 | 161.9468 | DOWN |
| RP11-473M20.9 | -2.67193 | -0.64569 | -20.8364 | 2.41E-75 | 4.11E-74 | 160.4543 | DOWN |
| RP6-74O6.2 | -2.40937 | -2.55723 | -20.7835 | 4.77E-75 | 8.10E-74 | 159.7748 | DOWN |
| AC016730.1 | -4.2739 | -8.01447 | -20.7786 | 5.07E-75 | 8.60E-74 | 159.7124 | DOWN |
| RP11-676J12.4 | -2.52104 | -2.52926 | -20.7511 | 7.23E-75 | 1.22E-73 | 159.3582 | DOWN |
| AC004947.2 | -4.17068 | -6.18294 | -20.7007 | 1.38E-74 | 2.33E-73 | 158.7112 | DOWN |
| C15orf59-AS1 | -4.58173 | -6.04177 | -20.6399 | 3.02E-74 | 5.08E-73 | 157.9314 | DOWN |
| AC006372.4 | -2.72896 | -8.58389 | -20.5989 | 5.11E-74 | 8.57E-73 | 157.406 | DOWN |
| RP11-147L13.8 | -3.34268 | -4.00249 | -20.5758 | 6.88E-74 | 1.15E-72 | 157.1099 | DOWN |
| RP5-1057I20.2 | -4.90853 | -7.61837 | -20.5619 | 8.22E-74 | 1.37E-72 | 156.9317 | DOWN |
| AC098823.3 | -3.24081 | -6.06688 | -20.538 | 1.12E-73 | 1.86E-72 | 156.6252 | DOWN |
| KB-208E9.1 | -4.22451 | -3.6684 | -20.4065 | 6.03E-73 | 1.00E-71 | 154.9422 | DOWN |
| AP000344.3 | -5.16308 | -5.83923 | -20.3882 | 7.62E-73 | 1.26E-71 | 154.7085 | DOWN |
| RP11-1096D5.2 | -4.97073 | -8.06899 | -20.3295 | 1.62E-72 | 2.67E-71 | 153.9577 | DOWN |
| AC093642.3 | -3.90703 | -1.42447 | -20.2182 | 6.71E-72 | 1.09E-70 | 152.5366 | DOWN |
| AC005152.3 | -6.93675 | -2.88067 | -20.2016 | 8.29E-72 | 1.35E-70 | 152.3251 | DOWN |
| PCAT18 | -4.15238 | -6.94146 | -20.1961 | 8.90E-72 | 1.44E-70 | 152.2549 | DOWN |
| RP11-73G16.2 | -5.17222 | -7.10429 | -20.1896 | 9.66E-72 | 1.57E-70 | 152.1722 | DOWN |
| RP4-669P10.16 | -4.80793 | -5.79544 | -20.1792 | 1.10E-71 | 1.79E-70 | 152.0388 | DOWN |
| CTC-542B22.2 | -3.60448 | -0.97492 | -20.1646 | 1.33E-71 | 2.15E-70 | 151.853 | DOWN |
| RP11-175K6.1 | -2.26182 | -0.1827 | -20.1185 | 2.40E-71 | 3.84E-70 | 151.2654 | DOWN |
| RP11-490M8.1 | -4.31005 | -0.67215 | -20.1142 | 2.53E-71 | 4.05E-70 | 151.2108 | DOWN |
| CTA-992D9.11 | -2.25347 | -9.0102 | -20.095 | 3.24E-71 | 5.17E-70 | 150.9658 | DOWN |
| RP11-429J17.7 | -3.7198 | -4.19359 | -20.0886 | 3.51E-71 | 5.59E-70 | 150.8846 | DOWN |
| RP1-137D17.1 | -4.54962 | -5.07806 | -20.0687 | 4.52E-71 | 7.20E-70 | 150.6311 | DOWN |
| LINC00844 | -5.98545 | -5.12295 | -20.0311 | 7.31E-71 | 1.16E-69 | 150.1518 | DOWN |
| RP11-94C24.6 | -5.34508 | -6.9975 | -19.9928 | 1.19E-70 | 1.88E-69 | 149.6647 | DOWN |
| FAM157C | -2.46327 | 0.567752 | -19.9604 | 1.80E-70 | 2.83E-69 | 149.2533 | DOWN |
| AC006262.5 | -6.60398 | -1.74297 | -19.9537 | 1.96E-70 | 3.08E-69 | 149.1677 | DOWN |
| RP5-1057J7.7 | -4.40424 | -3.00062 | -19.8874 | 4.56E-70 | 7.14E-69 | 148.3243 | DOWN |
| CTB-43E15.1 | -4.57968 | -7.68065 | -19.8636 | 6.17E-70 | 9.62E-69 | 148.0224 | DOWN |
| RP11-168F9.2 | -5.38374 | -6.25765 | -19.8115 | 1.20E-69 | 1.86E-68 | 147.3617 | DOWN |
| AC144525.1 | -3.79207 | -8.47835 | -19.8019 | 1.35E-69 | 2.09E-68 | 147.2393 | DOWN |
| RP11-196G11.2 | -4.95209 | -2.74074 | -19.729 | 3.42E-69 | 5.25E-68 | 146.3146 | DOWN |
| RP11-13K12.5 | -4.14622 | -6.19101 | -19.6948 | 5.27E-69 | 8.07E-68 | 145.8816 | DOWN |
| RP11-234K19.1 | -4.19815 | -8.38357 | -19.677 | 6.61E-69 | 1.01E-67 | 145.6559 | DOWN |
| SNHG23 | -5.20276 | -6.02548 | -19.6643 | 7.76E-69 | 1.18E-67 | 145.495 | DOWN |
| RP11-65J21.3 | -4.10077 | -0.84074 | -19.6635 | 7.84E-69 | 1.19E-67 | 145.4855 | DOWN |
| LINC01355 | -2.31668 | 0.012651 | -19.6288 | 1.22E-68 | 1.85E-67 | 145.0461 | DOWN |
| RP11-1006G14.1 | -3.25463 | -6.02112 | -19.6078 | 1.59E-68 | 2.41E-67 | 144.7797 | DOWN |
| RP11-375I20.6 | -3.4362 | -3.50329 | -19.5617 | 2.85E-68 | 4.29E-67 | 144.1969 | DOWN |
| RP11-497E19.1 | -3.7492 | -4.77345 | -19.5509 | 3.27E-68 | 4.91E-67 | 144.0603 | DOWN |
| CTD-2541J13.1 | -3.29003 | -5.51224 | -19.5277 | 4.38E-68 | 6.58E-67 | 143.767 | DOWN |
| RP11-526I2.5 | -2.40533 | -0.12093 | -19.5247 | 4.55E-68 | 6.82E-67 | 143.7292 | DOWN |
| RP11-6L6.2 | -5.52999 | -5.43099 | -19.5182 | 4.94E-68 | 7.39E-67 | 143.6472 | DOWN |
| CTD-2007L18.5 | -4.20928 | -2.00002 | -19.4536 | 1.12E-67 | 1.67E-66 | 142.8319 | DOWN |
| RP11-1057B6.1 | -5.00986 | -6.06302 | -19.4383 | 1.36E-67 | 2.02E-66 | 142.6388 | DOWN |
| RP11-433C9.2 | -3.55019 | -8.34619 | -19.4123 | 1.89E-67 | 2.79E-66 | 142.3107 | DOWN |
| CTC-526N19.1 | -2.69417 | 0.807654 | -19.4047 | 2.08E-67 | 3.07E-66 | 142.2144 | DOWN |
| RP11-44F14.8 | -3.79147 | -6.34499 | -19.3964 | 2.31E-67 | 3.40E-66 | 142.1101 | DOWN |
| RP11-497H17.1 | -4.44993 | -2.24312 | -19.3434 | 4.51E-67 | 6.63E-66 | 141.4412 | DOWN |
| PRR31 | -3.47078 | -8.66493 | -19.3385 | 4.79E-67 | 7.04E-66 | 141.3793 | DOWN |
| AF131217.1 | -3.39966 | -5.39121 | -19.2325 | 1.83E-66 | 2.67E-65 | 140.0448 | DOWN |
| RP11-456H18.2 | -4.54068 | -4.02207 | -19.214 | 2.30E-66 | 3.35E-65 | 139.8122 | DOWN |
| AC007365.1 | -3.21446 | -8.21016 | -19.201 | 2.71E-66 | 3.93E-65 | 139.6487 | DOWN |
| RP11-563J2.2 | -2.40713 | -4.00675 | -19.1329 | 6.40E-66 | 9.22E-65 | 138.7927 | DOWN |
| CTD-2540B15.9 | -4.85362 | -7.15708 | -19.1119 | 8.33E-66 | 1.20E-64 | 138.5292 | DOWN |
| LINC01449 | -4.09567 | -6.93236 | -19.095 | 1.03E-65 | 1.48E-64 | 138.3169 | DOWN |
| AC144831.3 | -2.57347 | -0.32739 | -19.0681 | 1.44E-65 | 2.07E-64 | 137.9802 | DOWN |
| RP11-507B12.2 | -3.08968 | -8.6136 | -18.9783 | 4.46E-65 | 6.34E-64 | 136.8542 | DOWN |
| C9orf106 | -3.18369 | -6.41573 | -18.966 | 5.21E-65 | 7.38E-64 | 136.7 | DOWN |
| RP11-508N22.12 | -2.43074 | -4.22481 | -18.9373 | 7.47E-65 | 1.06E-63 | 136.34 | DOWN |
| RP11-44N12.5 | -5.0981 | -5.08989 | -18.8934 | 1.29E-64 | 1.82E-63 | 135.7913 | DOWN |
| RP4-781K5.4 | -3.91143 | -7.76122 | -18.8683 | 1.77E-64 | 2.49E-63 | 135.4775 | DOWN |
| AC005082.12 | -2.9887 | -3.41247 | -18.8594 | 1.98E-64 | 2.77E-63 | 135.3663 | DOWN |
| RP11-333E1.2 | -3.59192 | -1.43903 | -18.8561 | 2.07E-64 | 2.89E-63 | 135.3248 | DOWN |
| AC092620.3 | -4.58208 | -6.70293 | -18.8106 | 3.65E-64 | 5.09E-63 | 134.7563 | DOWN |
| MIRLET7BHG | -2.3511 | 1.147443 | -18.7902 | 4.72E-64 | 6.56E-63 | 134.5012 | DOWN |
| RP11-237N19.3 | -3.90098 | -8.31889 | -18.7553 | 7.29E-64 | 1.01E-62 | 134.066 | DOWN |
| RP11-502M1.2 | -3.08399 | -8.70678 | -18.7519 | 7.61E-64 | 1.05E-62 | 134.0234 | DOWN |
| RP5-897D18.1 | -4.86475 | -7.11792 | -18.7282 | 1.02E-63 | 1.41E-62 | 133.7269 | DOWN |
| RP11-143E21.3 | -3.25835 | -8.47858 | -18.7267 | 1.04E-63 | 1.43E-62 | 133.7084 | DOWN |
| RP11-379P15.1 | -2.15312 | -9.11247 | -18.7243 | 1.08E-63 | 1.47E-62 | 133.6788 | DOWN |
| RP5-1159O4.1 | -2.52773 | -0.28755 | -18.6933 | 1.58E-63 | 2.17E-62 | 133.2916 | DOWN |
| LINC01168 | -2.23426 | -8.99029 | -18.6741 | 2.01E-63 | 2.73E-62 | 133.0529 | DOWN |
| AC009299.3 | -4.00701 | -2.9088 | -18.6578 | 2.47E-63 | 3.34E-62 | 132.8503 | DOWN |
| RP11-1060G2.2 | -3.09223 | -5.86488 | -18.6546 | 2.57E-63 | 3.47E-62 | 132.8097 | DOWN |
| RP5-1063M23.2 | -3.47143 | -7.02516 | -18.6134 | 4.29E-63 | 5.79E-62 | 132.2968 | DOWN |
| RP11-429A20.4 | -4.08155 | -7.78298 | -18.5592 | 8.44E-63 | 1.13E-61 | 131.6219 | DOWN |
| RP11-434P11.2 | -3.83532 | -2.691 | -18.5543 | 8.97E-63 | 1.20E-61 | 131.5618 | DOWN |
| RP11-452H21.4 | -3.03647 | -2.7999 | -18.5131 | 1.50E-62 | 2.00E-61 | 131.0498 | DOWN |
| RP11-546K22.1 | -4.21751 | -3.67262 | -18.4929 | 1.93E-62 | 2.56E-61 | 130.7989 | DOWN |
| RP11-798G7.8 | -2.59734 | -1.8318 | -18.4902 | 1.99E-62 | 2.65E-61 | 130.7654 | DOWN |
| MIR222HG | -2.43193 | 1.323838 | -18.4892 | 2.02E-62 | 2.68E-61 | 130.753 | DOWN |
| RP5-866L20.1 | -3.77352 | -8.21738 | -18.4258 | 4.44E-62 | 5.87E-61 | 129.9658 | DOWN |
| LINC01354 | -3.21612 | -5.00243 | -18.403 | 5.90E-62 | 7.78E-61 | 129.6821 | DOWN |
| RP11-250B2.3 | -3.30439 | -1.28199 | -18.397 | 6.36E-62 | 8.37E-61 | 129.6077 | DOWN |
| LINC00614 | -4.04108 | -7.74641 | -18.3885 | 7.06E-62 | 9.27E-61 | 129.5028 | DOWN |
| RP11-277B15.3 | -2.52949 | -1.62886 | -18.377 | 8.14E-62 | 1.07E-60 | 129.3607 | DOWN |
| RP11-981G7.6 | -2.43265 | -2.09224 | -18.364 | 9.58E-62 | 1.25E-60 | 129.1987 | DOWN |
| RP11-130L8.1 | -3.01998 | -1.08155 | -18.3438 | 1.23E-61 | 1.61E-60 | 128.9494 | DOWN |
| XXbac-BPGBPG55C20.1 | -5.26933 | -4.20193 | -18.3369 | 1.34E-61 | 1.75E-60 | 128.8632 | DOWN |
| RP11-227G15.11 | -3.98972 | -2.26963 | -18.2887 | 2.44E-61 | 3.16E-60 | 128.2667 | DOWN |
| RP11-562A8.5 | -5.21962 | -6.78158 | -18.2677 | 3.16E-61 | 4.09E-60 | 128.0076 | DOWN |
| CTD-3051D23.1 | -2.78443 | -4.71526 | -18.2276 | 5.19E-61 | 6.69E-60 | 127.5113 | DOWN |
| RP11-244G12.1 | -2.72364 | -8.52188 | -18.2186 | 5.81E-61 | 7.46E-60 | 127.4004 | DOWN |
| RP11-732A21.2 | -2.82427 | -8.39585 | -18.2177 | 5.87E-61 | 7.53E-60 | 127.3888 | DOWN |
| RP4-781K5.9 | -3.68009 | -8.43523 | -18.2077 | 6.65E-61 | 8.51E-60 | 127.2654 | DOWN |
| RP5-903G2.2 | -4.21417 | -5.23066 | -18.1924 | 8.03E-61 | 1.02E-59 | 127.0767 | DOWN |
| AC128709.3 | -4.37962 | -7.27084 | -18.1765 | 9.78E-61 | 1.24E-59 | 126.88 | DOWN |
| AC012363.13 | -3.49301 | -8.59066 | -18.1751 | 9.95E-61 | 1.26E-59 | 126.8625 | DOWN |
| RP11-244M2.1 | -4.14395 | -4.69154 | -18.1639 | 1.14E-60 | 1.45E-59 | 126.7242 | DOWN |
| RP13-714J12.1 | -4.46767 | -6.22105 | -18.1621 | 1.17E-60 | 1.48E-59 | 126.7028 | DOWN |
| RP11-831A10.1 | -3.69506 | -8.6486 | -18.1421 | 1.50E-60 | 1.89E-59 | 126.4559 | DOWN |
| RP1-224A6.9 | -3.78042 | -3.86539 | -18.1099 | 2.23E-60 | 2.80E-59 | 126.0584 | DOWN |
| AF011889.2 | -4.33374 | -2.63543 | -18.107 | 2.31E-60 | 2.89E-59 | 126.0226 | DOWN |
| CMP21-97G8.2 | -4.43358 | -7.24342 | -18.0459 | 4.91E-60 | 6.13E-59 | 125.2699 | DOWN |
| LINC00392 | -3.5026 | -8.61324 | -18.0426 | 5.12E-60 | 6.38E-59 | 125.2287 | DOWN |
| RP11-20G13.5 | -4.45279 | -4.42193 | -18.0004 | 8.61E-60 | 1.07E-58 | 124.7094 | DOWN |
| AF064858.6 | -4.02415 | -4.51387 | -17.9878 | 1.01E-59 | 1.25E-58 | 124.5548 | DOWN |
| RP4-669P10.20 | -2.66831 | -1.47168 | -17.9208 | 2.29E-59 | 2.82E-58 | 123.7315 | DOWN |
| AL161668.5 | -4.65086 | -6.63356 | -17.8998 | 2.97E-59 | 3.64E-58 | 123.4732 | DOWN |
| RP11-19E11.1 | -5.25509 | -4.10731 | -17.869 | 4.34E-59 | 5.28E-58 | 123.0954 | DOWN |
| RP11-105N14.1 | -3.93194 | -2.69666 | -17.8489 | 5.56E-59 | 6.76E-58 | 122.8482 | DOWN |
| AC116035.1 | -4.78283 | -6.56804 | -17.8482 | 5.61E-59 | 6.80E-58 | 122.8398 | DOWN |
| RP5-1159O4.2 | -3.0977 | -2.89895 | -17.806 | 9.42E-59 | 1.13E-57 | 122.3223 | DOWN |
| DGCR9 | -3.1711 | -3.42931 | -17.7506 | 1.86E-58 | 2.23E-57 | 121.6447 | DOWN |
| RP11-452L6.1 | -2.14864 | -0.04096 | -17.7076 | 3.15E-58 | 3.76E-57 | 121.1182 | DOWN |
| RP6-65G23.3 | -3.66763 | -0.5583 | -17.6661 | 5.24E-58 | 6.22E-57 | 120.6107 | DOWN |
| RP1-56K13.5 | -5.04276 | -4.93966 | -17.6508 | 6.31E-58 | 7.48E-57 | 120.4246 | DOWN |
| PGM5P4-AS1 | -4.81432 | -5.4363 | -17.6212 | 9.08E-58 | 1.07E-56 | 120.0625 | DOWN |
| CTD-2378E12.1 | -3.393 | -5.87976 | -17.5621 | 1.87E-57 | 2.20E-56 | 119.3413 | DOWN |
| RP11-101E5.1 | -3.19394 | -8.6296 | -17.5528 | 2.09E-57 | 2.46E-56 | 119.228 | DOWN |
| LINC01515 | -4.53778 | -1.24158 | -17.5091 | 3.57E-57 | 4.17E-56 | 118.6966 | DOWN |
| AC006273.4 | -3.91143 | -6.6429 | -17.4965 | 4.16E-57 | 4.85E-56 | 118.5432 | DOWN |
| LINC00671 | -3.40465 | -7.25795 | -17.4951 | 4.23E-57 | 4.92E-56 | 118.5264 | DOWN |
| LINC01558 | -3.29823 | -4.00552 | -17.4923 | 4.38E-57 | 5.09E-56 | 118.4913 | DOWN |
| RP11-115J16.1 | -4.23117 | -5.79825 | -17.4853 | 4.77E-57 | 5.53E-56 | 118.4069 | DOWN |
| RP11-109J4.1 | -3.72581 | -6.758 | -17.4487 | 7.45E-57 | 8.60E-56 | 117.9616 | DOWN |
| RP11-392O17.1 | -4.45041 | -3.67274 | -17.4453 | 7.77E-57 | 8.94E-56 | 117.9204 | DOWN |
| CTD-3116E22.7 | -4.27467 | -7.03135 | -17.4127 | 1.16E-56 | 1.33E-55 | 117.5237 | DOWN |
| RP11-538D16.3 | -3.60465 | -7.11066 | -17.3771 | 1.78E-56 | 2.04E-55 | 117.0921 | DOWN |
| RP11-97N19.3 | -2.44732 | -9.13853 | -17.3745 | 1.84E-56 | 2.10E-55 | 117.0604 | DOWN |
| RP4-625H18.2 | -4.02964 | -2.4883 | -17.2995 | 4.58E-56 | 5.20E-55 | 116.1506 | DOWN |
| LINC01265 | -4.13069 | -6.33077 | -17.2597 | 7.43E-56 | 8.37E-55 | 115.6683 | DOWN |
| RP13-870H17.3 | -3.5857 | -6.22191 | -17.2169 | 1.25E-55 | 1.40E-54 | 115.151 | DOWN |
| ZNF833P | -2.46627 | -2.99723 | -17.2018 | 1.50E-55 | 1.68E-54 | 114.9673 | DOWN |
| RP11-932O9.9 | -2.69867 | 0.18241 | -17.1821 | 1.90E-55 | 2.12E-54 | 114.7299 | DOWN |
| RP11-338N10.1 | -3.5068 | -6.65828 | -17.151 | 2.77E-55 | 3.08E-54 | 114.3538 | DOWN |
| RP11-368L12.1 | -4.94751 | -4.15631 | -17.1455 | 2.96E-55 | 3.29E-54 | 114.2879 | DOWN |
| RP11-144A16.8 | -4.2418 | -7.74471 | -17.1383 | 3.23E-55 | 3.58E-54 | 114.201 | DOWN |
| RP5-965F6.2 | -3.66456 | -8.11612 | -17.1139 | 4.34E-55 | 4.80E-54 | 113.9066 | DOWN |
| RP11-388C12.5 | -4.98445 | -3.1104 | -17.0913 | 5.71E-55 | 6.29E-54 | 113.6336 | DOWN |
| CTD-2554C21.3 | -3.39702 | -4.40184 | -17.0777 | 6.73E-55 | 7.37E-54 | 113.47 | DOWN |
| MAFTRR | -3.29728 | -1.94496 | -17.0614 | 8.19E-55 | 8.95E-54 | 113.2735 | DOWN |
| RP11-468E2.5 | -2.40025 | -1.25008 | -17.0546 | 8.90E-55 | 9.71E-54 | 113.1907 | DOWN |
| AF131215.8 | -4.13761 | -3.90608 | -17.0478 | 9.66E-55 | 1.05E-53 | 113.1091 | DOWN |
| LA16c-OS12.2 | -2.81602 | -1.34911 | -17.0359 | 1.12E-54 | 1.21E-53 | 112.966 | DOWN |
| RP11-171I2.4 | -4.01267 | -1.7981 | -17.0087 | 1.55E-54 | 1.67E-53 | 112.6383 | DOWN |
| RP11-758N13.1 | -3.80587 | -6.52832 | -16.9854 | 2.05E-54 | 2.21E-53 | 112.3577 | DOWN |
| RP11-406H23.2 | -4.39271 | -6.20518 | -16.9806 | 2.17E-54 | 2.34E-53 | 112.3009 | DOWN |
| RP1-269M15.3 | -2.97861 | -8.27053 | -16.9599 | 2.79E-54 | 3.00E-53 | 112.0517 | DOWN |
| RP11-440I14.2 | -3.68217 | -5.92075 | -16.946 | 3.30E-54 | 3.54E-53 | 111.8841 | DOWN |
| RP11-1094H24.4 | -2.95836 | -3.32301 | -16.9438 | 3.39E-54 | 3.63E-53 | 111.8579 | DOWN |
| CH17-174L20.1 | -3.26675 | -8.73644 | -16.9305 | 3.98E-54 | 4.25E-53 | 111.6983 | DOWN |
| RP11-111F5.3 | -3.97054 | -2.2414 | -16.9289 | 4.05E-54 | 4.32E-53 | 111.6796 | DOWN |
| CTD-2192J16.26 | -3.43844 | -3.09558 | -16.9219 | 4.41E-54 | 4.69E-53 | 111.5953 | DOWN |
| FLJ41941 | -3.40786 | -7.71306 | -16.904 | 5.47E-54 | 5.80E-53 | 111.3806 | DOWN |
| FAM138B | -3.58567 | -7.99363 | -16.8938 | 6.18E-54 | 6.54E-53 | 111.2579 | DOWN |
| RP11-333I13.1 | -2.07101 | -3.67129 | -16.8697 | 8.26E-54 | 8.73E-53 | 110.9684 | DOWN |
| RP11-117L5.4 | -3.22721 | -8.81544 | -16.8358 | 1.24E-53 | 1.31E-52 | 110.5626 | DOWN |
| GS1-39E22.2 | -3.58233 | -8.54083 | -16.8269 | 1.38E-53 | 1.45E-52 | 110.4562 | DOWN |
| RP1-302G2.5 | -3.67448 | -3.21087 | -16.8208 | 1.49E-53 | 1.56E-52 | 110.382 | DOWN |
| LINC01186 | -4.3301 | -6.40542 | -16.808 | 1.73E-53 | 1.82E-52 | 110.2291 | DOWN |
| LINC00632 | -4.36622 | -3.56362 | -16.8044 | 1.81E-53 | 1.90E-52 | 110.1855 | DOWN |
| RP1-149A16.3 | -2.14198 | -3.21122 | -16.7965 | 1.99E-53 | 2.08E-52 | 110.0915 | DOWN |
| OSMR-AS1 | -4.00321 | -3.38485 | -16.7255 | 4.66E-53 | 4.86E-52 | 109.2425 | DOWN |
| LINC01123 | -3.18768 | -0.78627 | -16.7036 | 6.07E-53 | 6.31E-52 | 108.9802 | DOWN |
| LINC00843 | -4.36269 | -4.08218 | -16.6963 | 6.61E-53 | 6.87E-52 | 108.894 | DOWN |
| AC008746.12 | -2.08017 | -0.94298 | -16.6838 | 7.69E-53 | 7.97E-52 | 108.7442 | DOWN |
| LINC00403 | -4.50342 | -6.93518 | -16.6692 | 9.16E-53 | 9.49E-52 | 108.5696 | DOWN |
| RP11-504A18.1 | -3.57821 | -5.66781 | -16.6194 | 1.66E-52 | 1.71E-51 | 107.9763 | DOWN |
| RNU12 | -4.10134 | -4.92092 | -16.6033 | 2.01E-52 | 2.07E-51 | 107.7843 | DOWN |
| LINC00165 | -4.02086 | -6.67246 | -16.5581 | 3.45E-52 | 3.53E-51 | 107.2462 | DOWN |
| RP11-1007O24.2 | -2.54849 | -2.45619 | -16.5327 | 4.67E-52 | 4.76E-51 | 106.9448 | DOWN |
| RP11-38L15.2 | -2.55633 | -1.38643 | -16.5321 | 4.71E-52 | 4.79E-51 | 106.9371 | DOWN |
| CTC-254B4.1 | -3.44693 | -8.29217 | -16.5115 | 6.01E-52 | 6.10E-51 | 106.6925 | DOWN |
| LINC00930 | -4.0003 | -3.80942 | -16.4998 | 6.92E-52 | 7.00E-51 | 106.5529 | DOWN |
| RP11-54O7.3 | -2.57754 | -2.89438 | -16.4759 | 9.19E-52 | 9.26E-51 | 106.2696 | DOWN |
| PGM5P3-AS1 | -4.41118 | -4.87997 | -16.457 | 1.15E-51 | 1.16E-50 | 106.0448 | DOWN |
| AC019117.1 | -3.8683 | -6.87036 | -16.4327 | 1.54E-51 | 1.54E-50 | 105.7574 | DOWN |
| RP11-347I19.8 | -2.37289 | 0.057053 | -16.4274 | 1.64E-51 | 1.63E-50 | 105.6945 | DOWN |
| RP11-316M20.1 | -2.40571 | -8.76994 | -16.4059 | 2.11E-51 | 2.11E-50 | 105.4387 | DOWN |
| RP4-734C18.1 | -2.99715 | -8.68448 | -16.395 | 2.41E-51 | 2.39E-50 | 105.3103 | DOWN |
| RP11-119D9.1 | -3.88569 | -5.34964 | -16.3311 | 5.13E-51 | 5.06E-50 | 104.5541 | DOWN |
| RP11-96C21.2 | -2.57664 | -5.91859 | -16.331 | 5.14E-51 | 5.06E-50 | 104.5529 | DOWN |
| RP11-290F5.2 | -3.19682 | -5.42578 | -16.2964 | 7.75E-51 | 7.61E-50 | 104.1431 | DOWN |
| RP11-850F7.7 | -2.87585 | -8.93651 | -16.2548 | 1.27E-50 | 1.24E-49 | 103.652 | DOWN |
| AC012531.25 | -2.46598 | -2.19078 | -16.2385 | 1.54E-50 | 1.50E-49 | 103.4603 | DOWN |
| FAM87B | -2.22909 | -2.82084 | -16.2341 | 1.62E-50 | 1.58E-49 | 103.4081 | DOWN |
| RP4-737E23.7 | -2.68332 | -8.97517 | -16.234 | 1.62E-50 | 1.58E-49 | 103.407 | DOWN |
| AC016582.2 | -3.45584 | -6.9873 | -16.2271 | 1.76E-50 | 1.72E-49 | 103.325 | DOWN |
| CTD-2044J15.1 | -3.03914 | -6.02736 | -16.1493 | 4.41E-50 | 4.27E-49 | 102.4091 | DOWN |
| RP11-236L14.2 | -2.84288 | -3.61802 | -16.1358 | 5.18E-50 | 5.00E-49 | 102.2503 | DOWN |
| RP11-475B2.1 | -2.97728 | -8.52597 | -16.131 | 5.48E-50 | 5.29E-49 | 102.1934 | DOWN |
| LA16c-385E7.1 | -3.84139 | -5.52525 | -16.1273 | 5.72E-50 | 5.51E-49 | 102.1501 | DOWN |
| RP11-794G24.1 | -3.26645 | -5.95195 | -16.1214 | 6.13E-50 | 5.90E-49 | 102.0815 | DOWN |
| RP11-401P9.4 | -2.7962 | -0.84008 | -16.1119 | 6.86E-50 | 6.58E-49 | 101.9698 | DOWN |
| RP11-330M19.1 | -3.10889 | -7.2083 | -16.0732 | 1.08E-49 | 1.04E-48 | 101.515 | DOWN |
| RP5-1158E12.3 | -4.38348 | -7.10073 | -16.0645 | 1.20E-49 | 1.15E-48 | 101.4122 | DOWN |
| LINC01126 | -2.01999 | -2.05484 | -16.0547 | 1.35E-49 | 1.28E-48 | 101.2976 | DOWN |
| C14orf132 | -2.56903 | 1.099185 | -16.044 | 1.53E-49 | 1.45E-48 | 101.1723 | DOWN |
| RP11-274B21.10 | -2.04005 | -1.30891 | -16.0284 | 1.83E-49 | 1.74E-48 | 100.9891 | DOWN |
| RP11-536I6.2 | -3.86307 | -8.07548 | -16.0091 | 2.30E-49 | 2.18E-48 | 100.7628 | DOWN |
| RP11-624L4.1 | -3.86974 | -4.60868 | -15.9597 | 4.11E-49 | 3.88E-48 | 100.1838 | DOWN |
| RP11-734K23.9 | -2.56343 | 0.892757 | -15.9592 | 4.14E-49 | 3.90E-48 | 100.1781 | DOWN |
| RP11-147L13.14 | -3.95164 | -4.20662 | -15.9471 | 4.76E-49 | 4.48E-48 | 100.0372 | DOWN |
| LINC01124 | -3.52023 | -4.5809 | -15.938 | 5.30E-49 | 4.98E-48 | 99.93004 | DOWN |
| RP5-1063M23.1 | -2.94518 | -7.38703 | -15.9235 | 6.29E-49 | 5.89E-48 | 99.76094 | DOWN |
| RP11-83M16.5 | -2.9489 | -8.74399 | -15.9226 | 6.35E-49 | 5.95E-48 | 99.75008 | DOWN |
| AC128709.2 | -3.14507 | -8.54744 | -15.9198 | 6.57E-49 | 6.14E-48 | 99.71715 | DOWN |
| RP11-10L7.1 | -3.27019 | -5.44072 | -15.9022 | 8.06E-49 | 7.52E-48 | 99.51253 | DOWN |
| LINC00365 | -3.37225 | -4.83879 | -15.8544 | 1.41E-48 | 1.31E-47 | 98.95387 | DOWN |
| CH507-396I9.3 | -3.12793 | -6.46731 | -15.8493 | 1.50E-48 | 1.39E-47 | 98.89478 | DOWN |
| RP11-121M22.1 | -3.51641 | -4.30072 | -15.8468 | 1.54E-48 | 1.43E-47 | 98.8651 | DOWN |
| RP13-616I3.1 | -2.12433 | -2.13279 | -15.8453 | 1.57E-48 | 1.45E-47 | 98.84759 | DOWN |
| RP11-663N22.1 | -3.20163 | -8.47656 | -15.8132 | 2.29E-48 | 2.10E-47 | 98.474 | DOWN |
| RP11-673E11.2 | -2.26075 | -9.03175 | -15.7951 | 2.82E-48 | 2.59E-47 | 98.26294 | DOWN |
| RP11-245J9.5 | -2.10205 | -0.98763 | -15.7845 | 3.20E-48 | 2.92E-47 | 98.13987 | DOWN |
| RP11-218F10.3 | -2.56145 | -0.99239 | -15.7799 | 3.37E-48 | 3.08E-47 | 98.08535 | DOWN |
| RP6-74O6.6 | -4.08807 | -3.19824 | -15.7781 | 3.45E-48 | 3.15E-47 | 98.06449 | DOWN |
| AC099552.3 | -3.05265 | -8.93367 | -15.7612 | 4.20E-48 | 3.83E-47 | 97.86793 | DOWN |
| CTD-2081C10.7 | -2.12232 | -1.36409 | -15.7142 | 7.26E-48 | 6.59E-47 | 97.32168 | DOWN |
| RP11-433J8.1 | -3.22808 | -6.34907 | -15.7101 | 7.61E-48 | 6.91E-47 | 97.27458 | DOWN |
| CCAT2 | -2.74643 | -8.06494 | -15.6939 | 9.19E-48 | 8.32E-47 | 97.08624 | DOWN |
| RP11-475I24.3 | -2.78378 | -1.45544 | -15.685 | 1.02E-47 | 9.22E-47 | 96.98264 | DOWN |
| MIR99AHG | -3.01971 | 0.444042 | -15.6752 | 1.14E-47 | 1.03E-46 | 96.86924 | DOWN |
| LINC00886 | -2.38428 | -1.70822 | -15.6674 | 1.25E-47 | 1.13E-46 | 96.77852 | DOWN |
| RP11-83B20.1 | -2.64992 | -3.7254 | -15.6558 | 1.43E-47 | 1.29E-46 | 96.6436 | DOWN |
| RP11-106M7.1 | -2.61622 | -8.19231 | -15.6296 | 1.94E-47 | 1.74E-46 | 96.34082 | DOWN |
| RP6-109B7.2 | -2.50499 | -3.37416 | -15.6283 | 1.97E-47 | 1.77E-46 | 96.32489 | DOWN |
| CTD-2012K14.6 | -3.50787 | -2.91394 | -15.6199 | 2.17E-47 | 1.95E-46 | 96.22827 | DOWN |
| RP11-61L19.2 | -3.57981 | -3.49079 | -15.5449 | 5.19E-47 | 4.63E-46 | 95.36059 | DOWN |
| AC009229.5 | -2.90859 | -8.71843 | -15.5402 | 5.49E-47 | 4.88E-46 | 95.30558 | DOWN |
| CTD-2542L18.1 | -2.32371 | -7.92348 | -15.5215 | 6.81E-47 | 6.04E-46 | 95.08972 | DOWN |
| RP4-794I6.4 | -2.15702 | -2.61281 | -15.5215 | 6.82E-47 | 6.04E-46 | 95.08926 | DOWN |
| AL132709.8 | -3.58092 | -7.72231 | -15.5112 | 7.67E-47 | 6.79E-46 | 94.97122 | DOWN |
| RP11-12L8.1 | -2.7172 | -8.39885 | -15.4689 | 1.25E-46 | 1.11E-45 | 94.48232 | DOWN |
| RP11-305L7.6 | -4.6968 | -5.52894 | -15.4472 | 1.61E-46 | 1.42E-45 | 94.23265 | DOWN |
| RP1-137D17.2 | -3.09714 | -2.7071 | -15.4388 | 1.77E-46 | 1.56E-45 | 94.13565 | DOWN |
| RP5-1065P14.2 | -3.83072 | -7.42133 | -15.4379 | 1.79E-46 | 1.58E-45 | 94.12596 | DOWN |
| RP13-188A5.1 | -2.09359 | -1.30084 | -15.3858 | 3.27E-46 | 2.86E-45 | 93.52645 | DOWN |
| LINC00926 | -2.01039 | 0.139421 | -15.3544 | 4.70E-46 | 4.09E-45 | 93.16563 | DOWN |
| RP11-461L13.3 | -3.65868 | -3.09184 | -15.3493 | 4.98E-46 | 4.33E-45 | 93.10642 | DOWN |
| RP11-84A19.4 | -4.00947 | -5.38812 | -15.3374 | 5.72E-46 | 4.96E-45 | 92.96982 | DOWN |
| RP11-100E5.2 | -4.84809 | -6.76935 | -15.3304 | 6.20E-46 | 5.38E-45 | 92.88907 | DOWN |
| RP11-131L12.4 | -2.90083 | -1.71168 | -15.3131 | 7.56E-46 | 6.55E-45 | 92.69051 | DOWN |
| CTC-523E23.11 | -3.2378 | -1.38016 | -15.2571 | 1.44E-45 | 1.23E-44 | 92.04973 | DOWN |
| LINC01497 | -3.21599 | -8.4857 | -15.2469 | 1.62E-45 | 1.39E-44 | 91.93232 | DOWN |
| RP11-211G23.2 | -4.77204 | -3.21803 | -15.2436 | 1.68E-45 | 1.44E-44 | 91.89492 | DOWN |
| RP11-394I13.1 | -2.75227 | -5.068 | -15.2223 | 2.15E-45 | 1.83E-44 | 91.65066 | DOWN |
| AF064860.7 | -3.57393 | -6.90618 | -15.1992 | 2.80E-45 | 2.38E-44 | 91.3863 | DOWN |
| AC096579.13 | -4.25923 | -4.23549 | -15.1593 | 4.42E-45 | 3.76E-44 | 90.93138 | DOWN |
| RP11-467I17.1 | -3.91118 | -7.64185 | -15.1281 | 6.32E-45 | 5.36E-44 | 90.57523 | DOWN |
| RP11-831A10.2 | -3.04138 | -8.76227 | -15.0973 | 8.99E-45 | 7.60E-44 | 90.22364 | DOWN |
| RP11-58E21.4 | -2.93217 | -7.38054 | -15.0954 | 9.18E-45 | 7.75E-44 | 90.20255 | DOWN |
| KB-1184D12.1 | -3.72703 | -7.38125 | -15.0925 | 9.49E-45 | 8.00E-44 | 90.16958 | DOWN |
| RP11-356O9.1 | -3.59193 | -7.27597 | -15.0727 | 1.19E-44 | 1.00E-43 | 89.94464 | DOWN |
| LINC00664 | -2.90821 | -4.78743 | -15.0636 | 1.32E-44 | 1.11E-43 | 89.84002 | DOWN |
| RP11-315F22.1 | -3.82027 | -7.97142 | -15.0626 | 1.34E-44 | 1.12E-43 | 89.82913 | DOWN |
| RP11-62L18.3 | -3.24005 | -8.74521 | -15.0238 | 2.08E-44 | 1.73E-43 | 89.3882 | DOWN |
| AC004448.2 | -3.24723 | -8.27482 | -15.0134 | 2.34E-44 | 1.94E-43 | 89.26992 | DOWN |
| RP11-457M11.5 | -2.49821 | -0.65723 | -14.982 | 3.35E-44 | 2.76E-43 | 88.91379 | DOWN |
| RP11-266K4.14 | -2.04701 | -2.22749 | -14.9419 | 5.28E-44 | 4.35E-43 | 88.45868 | DOWN |
| RP11-392O17.2 | -2.17607 | -2.37456 | -14.9265 | 6.29E-44 | 5.18E-43 | 88.28455 | DOWN |
| LINC01415 | -2.69637 | -4.62062 | -14.8857 | 1.00E-43 | 8.21E-43 | 87.82248 | DOWN |
| LINC00032 | -2.44272 | -7.92039 | -14.8762 | 1.11E-43 | 9.14E-43 | 87.71471 | DOWN |
| CTD-2066L21.3 | -4.21988 | -6.86495 | -14.8463 | 1.56E-43 | 1.28E-42 | 87.37708 | DOWN |
| RP11-63M22.1 | -3.56254 | -7.75878 | -14.8414 | 1.65E-43 | 1.35E-42 | 87.32177 | DOWN |
| RP11-615I2.6 | -2.3895 | -6.28435 | -14.83 | 1.88E-43 | 1.54E-42 | 87.19319 | DOWN |
| RP11-1228E12.2 | -4.04048 | -3.41809 | -14.8285 | 1.91E-43 | 1.56E-42 | 87.17693 | DOWN |
| AC006272.1 | -4.22348 | -6.0753 | -14.8143 | 2.25E-43 | 1.83E-42 | 87.01638 | DOWN |
| RP11-462B18.2 | -3.77326 | -7.00842 | -14.8119 | 2.31E-43 | 1.88E-42 | 86.98963 | DOWN |
| RP11-626G11.6 | -2.7852 | -1.25557 | -14.7867 | 3.07E-43 | 2.49E-42 | 86.70546 | DOWN |
| RP11-385D13.3 | -4.12712 | -7.31799 | -14.7861 | 3.09E-43 | 2.51E-42 | 86.698 | DOWN |
| RP11-299L17.3 | -3.7025 | -7.06869 | -14.7471 | 4.80E-43 | 3.87E-42 | 86.25918 | DOWN |
| LINC01028 | -2.70263 | -8.7279 | -14.7316 | 5.72E-43 | 4.60E-42 | 86.0847 | DOWN |
| RP11-218C14.5 | -3.14555 | -8.48413 | -14.7199 | 6.53E-43 | 5.24E-42 | 85.95299 | DOWN |
| RP11-584P21.2 | -3.25854 | -4.94985 | -14.7147 | 6.93E-43 | 5.55E-42 | 85.89436 | DOWN |
| RP11-377D9.3 | -2.49596 | -6.09986 | -14.6591 | 1.30E-42 | 1.04E-41 | 85.27042 | DOWN |
| RP11-430C7.5 | -2.55929 | -2.99377 | -14.6425 | 1.56E-42 | 1.25E-41 | 85.08374 | DOWN |
| RP11-673E1.1 | -3.6243 | -6.30363 | -14.5408 | 4.89E-42 | 3.88E-41 | 83.94643 | DOWN |
| AC067960.1 | -2.47182 | -8.90963 | -14.5295 | 5.56E-42 | 4.40E-41 | 83.8199 | DOWN |
| RP11-357P18.2 | -2.83942 | -1.37946 | -14.5188 | 6.27E-42 | 4.96E-41 | 83.69996 | DOWN |
| LINC00840 | -2.81796 | -5.31778 | -14.5184 | 6.29E-42 | 4.97E-41 | 83.69656 | DOWN |
| RP11-304F15.6 | -2.71665 | -8.95375 | -14.4977 | 7.93E-42 | 6.26E-41 | 83.46522 | DOWN |
| RP11-408A13.3 | -3.89845 | -5.1361 | -14.4822 | 9.44E-42 | 7.43E-41 | 83.29183 | DOWN |
| IBA57-AS1 | -3.58732 | -3.62011 | -14.4695 | 1.09E-41 | 8.55E-41 | 83.15063 | DOWN |
| LA16c-380A1.1 | -4.05582 | -4.82265 | -14.4228 | 1.83E-41 | 1.44E-40 | 82.6313 | DOWN |
| RP5-1023B21.1 | -2.75736 | -8.68337 | -14.3535 | 3.97E-41 | 3.09E-40 | 81.86121 | DOWN |
| RP11-489G11.3 | -2.64635 | -7.36825 | -14.3255 | 5.42E-41 | 4.22E-40 | 81.55048 | DOWN |
| LINC01359 | -2.59155 | -2.99582 | -14.3159 | 6.03E-41 | 4.69E-40 | 81.44454 | DOWN |
| RP11-321A17.6 | -2.29578 | -0.85749 | -14.3099 | 6.44E-41 | 5.01E-40 | 81.37862 | DOWN |
| CTA-221G9.7 | -3.26133 | -8.17191 | -14.3084 | 6.56E-41 | 5.09E-40 | 81.3616 | DOWN |
| CTD-3051D23.4 | -3.29252 | -5.75705 | -14.3083 | 6.56E-41 | 5.09E-40 | 81.3609 | DOWN |
| AC073284.4 | -2.7402 | -8.91502 | -14.2977 | 7.38E-41 | 5.71E-40 | 81.2432 | DOWN |
| RP11-297M9.1 | -2.30142 | -9.1256 | -14.2897 | 8.07E-41 | 6.22E-40 | 81.15495 | DOWN |
| AC091801.1 | -3.49709 | -7.50552 | -14.262 | 1.10E-40 | 8.44E-40 | 80.84812 | DOWN |
| RP11-259O2.3 | -2.49906 | -9.0564 | -14.2416 | 1.38E-40 | 1.06E-39 | 80.62309 | DOWN |
| AC007317.1 | -2.28557 | -9.17668 | -14.2387 | 1.42E-40 | 1.09E-39 | 80.59092 | DOWN |
| RP11-463O9.9 | -2.06622 | -4.50099 | -14.2273 | 1.61E-40 | 1.23E-39 | 80.46494 | DOWN |
| RP11-554I8.2 | -3.30289 | -7.48381 | -14.2128 | 1.89E-40 | 1.45E-39 | 80.30474 | DOWN |
| C16orf82 | -2.51414 | -8.4054 | -14.2074 | 2.01E-40 | 1.53E-39 | 80.24499 | DOWN |
| RP11-578F21.6 | -3.63818 | -6.68273 | -14.1944 | 2.32E-40 | 1.77E-39 | 80.10156 | DOWN |
| RP11-44F14.2 | -3.70052 | -6.54295 | -14.1858 | 2.55E-40 | 1.94E-39 | 80.00738 | DOWN |
| RP11-43F13.4 | -2.58813 | -2.92393 | -14.1832 | 2.63E-40 | 2.00E-39 | 79.97826 | DOWN |
| PCAT14 | -3.47243 | -6.41249 | -14.1471 | 3.92E-40 | 2.96E-39 | 79.5815 | DOWN |
| FAM66B | -2.06484 | -1.55333 | -14.1241 | 5.05E-40 | 3.81E-39 | 79.32803 | DOWN |
| RP11-285E9.5 | -2.53959 | -8.88626 | -14.063 | 9.91E-40 | 7.42E-39 | 78.65656 | DOWN |
| XX-DJ76P10__A.2 | -2.6006 | -8.88783 | -14.037 | 1.32E-39 | 9.87E-39 | 78.37171 | DOWN |
| CTD-2501M5.1 | -2.70474 | -8.63615 | -14.0296 | 1.43E-39 | 1.07E-38 | 78.29135 | DOWN |
| RP11-723O4.9 | -2.00726 | -1.47255 | -14.0204 | 1.58E-39 | 1.18E-38 | 78.19012 | DOWN |
| AC092192.1 | -3.16646 | -6.2705 | -14.011 | 1.76E-39 | 1.31E-38 | 78.08741 | DOWN |
| FAM157B | -3.83951 | -3.45038 | -14.0109 | 1.76E-39 | 1.31E-38 | 78.08577 | DOWN |
| AC024560.2 | -2.89288 | -3.86468 | -14.0087 | 1.80E-39 | 1.34E-38 | 78.06237 | DOWN |
| RP11-131L12.3 | -2.05115 | -0.90244 | -13.9361 | 4.00E-39 | 2.94E-38 | 77.2683 | DOWN |
| RP11-179A10.1 | -2.91735 | -6.82543 | -13.9209 | 4.72E-39 | 3.46E-38 | 77.10253 | DOWN |
| AC105339.1 | -2.77656 | -7.28995 | -13.9025 | 5.77E-39 | 4.23E-38 | 76.90286 | DOWN |
| RP11-481G8.2 | -2.06499 | -8.72901 | -13.8785 | 7.50E-39 | 5.48E-38 | 76.6415 | DOWN |
| CTC-523E23.4 | -2.80054 | -4.59076 | -13.8767 | 7.65E-39 | 5.58E-38 | 76.62172 | DOWN |
| RP11-796E10.1 | -4.28516 | -7.01679 | -13.8737 | 7.90E-39 | 5.76E-38 | 76.58927 | DOWN |
| RP11-395N3.2 | -3.62397 | -4.08502 | -13.8715 | 8.10E-39 | 5.90E-38 | 76.56458 | DOWN |
| RP11-454K7.1 | -3.36364 | -7.44112 | -13.8309 | 1.26E-38 | 9.14E-38 | 76.12349 | DOWN |
| RP11-131N11.4 | -3.63457 | -7.48393 | -13.787 | 2.04E-38 | 1.47E-37 | 75.64629 | DOWN |
| RP11-94C24.13 | -2.34657 | -5.54309 | -13.7752 | 2.31E-38 | 1.67E-37 | 75.51914 | DOWN |
| RP11-495K9.5 | -3.37855 | -3.53054 | -13.7569 | 2.82E-38 | 2.03E-37 | 75.32089 | DOWN |
| CTC-448F2.4 | -2.90499 | -5.50155 | -13.7292 | 3.82E-38 | 2.75E-37 | 75.02021 | DOWN |
| AC012074.2 | -2.02427 | -2.2569 | -13.7232 | 4.08E-38 | 2.93E-37 | 74.95548 | DOWN |
| RP1-78O14.1 | -2.86549 | -5.04375 | -13.7169 | 4.36E-38 | 3.13E-37 | 74.88765 | DOWN |
| RP11-278L15.2 | -2.65144 | -8.34755 | -13.6936 | 5.62E-38 | 4.02E-37 | 74.63531 | DOWN |
| RP5-881P19.7 | -2.2907 | -6.85256 | -13.6624 | 7.88E-38 | 5.64E-37 | 74.29855 | DOWN |
| RP11-323C15.2 | -3.36315 | -8.07235 | -13.6513 | 8.89E-38 | 6.34E-37 | 74.17961 | DOWN |
| LINCR-0001 | -3.41888 | -3.76597 | -13.6504 | 8.97E-38 | 6.40E-37 | 74.17005 | DOWN |
| RP11-364P22.2 | -2.02039 | -9.21532 | -13.649 | 9.11E-38 | 6.49E-37 | 74.1548 | DOWN |
| CTD-2536I1.3 | -3.67212 | -6.87652 | -13.6468 | 9.33E-38 | 6.64E-37 | 74.13115 | DOWN |
| RP11-452C8.1 | -2.67483 | -8.51221 | -13.6415 | 9.89E-38 | 7.03E-37 | 74.07309 | DOWN |
| RP5-1119O21.2 | -2.06258 | -9.16011 | -13.6307 | 1.11E-37 | 7.89E-37 | 73.95681 | DOWN |
| LINC00313 | -3.46618 | -3.98968 | -13.5985 | 1.57E-37 | 1.11E-36 | 73.61024 | DOWN |
| RP11-428C19.5 | -3.59223 | -6.99638 | -13.597 | 1.60E-37 | 1.13E-36 | 73.59446 | DOWN |
| RP11-119J18.1 | -4.49929 | -4.87339 | -13.5886 | 1.75E-37 | 1.24E-36 | 73.50392 | DOWN |
| AC026904.1 | -2.51729 | -6.24299 | -13.5844 | 1.83E-37 | 1.29E-36 | 73.4589 | DOWN |
| RP11-266K4.13 | -2.79574 | -5.09701 | -13.5654 | 2.25E-37 | 1.58E-36 | 73.25448 | DOWN |
| CTC-1337H24.4 | -3.21398 | -2.85645 | -13.5643 | 2.28E-37 | 1.60E-36 | 73.24327 | DOWN |
| RP11-160E2.6 | -4.2997 | -4.44861 | -13.5618 | 2.34E-37 | 1.64E-36 | 73.21619 | DOWN |
| RP11-227G15.10 | -3.8395 | -1.90862 | -13.4591 | 7.07E-37 | 4.93E-36 | 72.11487 | DOWN |
| CTC-429P9.2 | -2.11255 | 0.436019 | -13.4523 | 7.61E-37 | 5.30E-36 | 72.04211 | DOWN |
| RP11-45M22.5 | -3.40666 | -6.80675 | -13.4425 | 8.44E-37 | 5.87E-36 | 71.938 | DOWN |
| RP4-781K5.6 | -2.27157 | -7.87938 | -13.421 | 1.06E-36 | 7.38E-36 | 71.70816 | DOWN |
| CCAT1 | -2.40544 | -8.37869 | -13.3917 | 1.46E-36 | 1.01E-35 | 71.3951 | DOWN |
| RP11-227G15.8 | -3.38504 | -6.89487 | -13.3846 | 1.57E-36 | 1.09E-35 | 71.31948 | DOWN |
| KB-68A7.1 | -3.18174 | -1.70903 | -13.3813 | 1.63E-36 | 1.12E-35 | 71.28489 | DOWN |
| RP4-529N6.2 | -3.86444 | -6.70613 | -13.3797 | 1.66E-36 | 1.14E-35 | 71.2676 | DOWN |
| RP11-468H14.2 | -2.60943 | -6.20565 | -13.349 | 2.30E-36 | 1.58E-35 | 70.94072 | DOWN |
| LINC00377 | -3.83759 | -5.64411 | -13.3413 | 2.50E-36 | 1.72E-35 | 70.85863 | DOWN |
| RP11-20B7.1 | -3.25128 | -7.32542 | -13.3163 | 3.26E-36 | 2.24E-35 | 70.59258 | DOWN |
| RP11-111J6.2 | -2.32604 | -2.56583 | -13.3145 | 3.33E-36 | 2.28E-35 | 70.57344 | DOWN |
| LINC01107 | -2.17061 | -8.46894 | -13.314 | 3.34E-36 | 2.29E-35 | 70.56823 | DOWN |
| LINC00939 | -2.1848 | -8.73856 | -13.2977 | 3.98E-36 | 2.72E-35 | 70.3953 | DOWN |
| LINC01537 | -2.58354 | -2.78984 | -13.2929 | 4.19E-36 | 2.86E-35 | 70.3437 | DOWN |
| RP11-83M16.6 | -2.977 | -8.25034 | -13.2851 | 4.55E-36 | 3.10E-35 | 70.26175 | DOWN |
| AC098828.2 | -2.41983 | -8.79508 | -13.275 | 5.07E-36 | 3.45E-35 | 70.15387 | DOWN |
| RP11-554D15.3 | -3.38753 | -7.33798 | -13.2704 | 5.32E-36 | 3.62E-35 | 70.10533 | DOWN |
| RP11-571I18.4 | -4.25467 | -3.62638 | -13.263 | 5.76E-36 | 3.91E-35 | 70.02635 | DOWN |
| RP11-1038A11.1 | -3.56423 | -6.31049 | -13.1948 | 1.19E-35 | 8.06E-35 | 69.30499 | DOWN |
| AC092162.1 | -2.45168 | -7.81845 | -13.1902 | 1.25E-35 | 8.46E-35 | 69.25582 | DOWN |
| LINC01330 | -3.19255 | -6.26525 | -13.1734 | 1.49E-35 | 1.01E-34 | 69.07834 | DOWN |
| TTTY2 | -2.50757 | -8.22776 | -13.1689 | 1.57E-35 | 1.06E-34 | 69.0314 | DOWN |
| RP11-352G9.1 | -3.30218 | -4.49028 | -13.1673 | 1.59E-35 | 1.08E-34 | 69.01424 | DOWN |
| CTD-2331H12.7 | -3.51912 | -4.66958 | -13.1592 | 1.74E-35 | 1.17E-34 | 68.92874 | DOWN |
| RP11-690P14.4 | -2.86458 | -5.37484 | -13.1486 | 1.94E-35 | 1.31E-34 | 68.81743 | DOWN |
| RP11-761E20.1 | -2.52092 | -7.06205 | -13.1478 | 1.96E-35 | 1.32E-34 | 68.80872 | DOWN |
| RP11-246A10.1 | -3.07541 | -7.93535 | -13.1425 | 2.07E-35 | 1.39E-34 | 68.75276 | DOWN |
| FAM41C | -2.74336 | -2.73396 | -13.1372 | 2.19E-35 | 1.47E-34 | 68.69721 | DOWN |
| AC007461.2 | -2.8364 | -6.45704 | -13.135 | 2.24E-35 | 1.50E-34 | 68.67318 | DOWN |
| RP11-599B13.3 | -3.31631 | -7.83005 | -13.126 | 2.47E-35 | 1.65E-34 | 68.57906 | DOWN |
| RP11-129K12.1 | -3.37693 | -4.89489 | -13.1202 | 2.62E-35 | 1.76E-34 | 68.5175 | DOWN |
| RP11-54O7.1 | -2.81438 | -5.33024 | -13.1106 | 2.90E-35 | 1.94E-34 | 68.41701 | DOWN |
| AP001046.6 | -2.23517 | -5.12444 | -13.0865 | 3.75E-35 | 2.50E-34 | 68.16246 | DOWN |
| RP11-323I15.5 | -2.75562 | -7.23289 | -13.0727 | 4.34E-35 | 2.88E-34 | 68.0179 | DOWN |
| RP11-322E11.2 | -2.27589 | -7.35798 | -13.0521 | 5.39E-35 | 3.57E-34 | 67.80139 | DOWN |
| RP11-253I19.3 | -3.69757 | -4.81773 | -13.0479 | 5.63E-35 | 3.73E-34 | 67.75704 | DOWN |
| MEG8 | -3.5948 | -6.33611 | -13.0162 | 7.87E-35 | 5.19E-34 | 67.42443 | DOWN |
| RP11-655M14.13 | -3.14771 | -4.07696 | -13.0145 | 8.01E-35 | 5.28E-34 | 67.40729 | DOWN |
| AP000962.2 | -2.28616 | -8.89937 | -13.0084 | 8.55E-35 | 5.63E-34 | 67.34259 | DOWN |
| LINC00445 | -2.2517 | -9.08074 | -12.9934 | 1.00E-34 | 6.58E-34 | 67.18602 | DOWN |
| RP11-545G3.1 | -3.2852 | -7.80265 | -12.9809 | 1.14E-34 | 7.49E-34 | 67.0551 | DOWN |
| LINC01093 | -2.50289 | -8.75502 | -12.9641 | 1.36E-34 | 8.92E-34 | 66.87957 | DOWN |
| AC007557.3 | -2.01249 | -9.2447 | -12.9605 | 1.41E-34 | 9.25E-34 | 66.84156 | DOWN |
| LINC00160 | -2.37309 | -8.84689 | -12.9601 | 1.42E-34 | 9.27E-34 | 66.83796 | DOWN |
| CTC-523E23.5 | -2.53361 | -4.96593 | -12.9574 | 1.46E-34 | 9.53E-34 | 66.80951 | DOWN |
| RP11-359J14.2 | -2.92299 | -3.51874 | -12.9209 | 2.14E-34 | 1.39E-33 | 66.4283 | DOWN |
| LL22NC03-75H12.2 | -3.03505 | -7.6018 | -12.9134 | 2.32E-34 | 1.51E-33 | 66.34953 | DOWN |
| XXbac-BPGBPG55C20.3 | -3.42379 | -6.80128 | -12.9062 | 2.50E-34 | 1.62E-33 | 66.27451 | DOWN |
| RP11-713P17.3 | -2.27751 | -2.06401 | -12.8713 | 3.60E-34 | 2.33E-33 | 65.91136 | DOWN |
| RP11-710E1.2 | -2.75781 | -8.52209 | -12.8398 | 5.01E-34 | 3.23E-33 | 65.5837 | DOWN |
| LINC00993 | -2.70381 | -8.8149 | -12.8314 | 5.47E-34 | 3.53E-33 | 65.49581 | DOWN |
| AC004012.1 | -3.59631 | -6.84889 | -12.8095 | 6.87E-34 | 4.42E-33 | 65.26866 | DOWN |
| CTD-3234P18.6 | -2.08458 | -3.41452 | -12.8065 | 7.09E-34 | 4.55E-33 | 65.23773 | DOWN |
| RP11-191L9.4 | -3.66735 | -2.28607 | -12.7973 | 7.80E-34 | 5.01E-33 | 65.14179 | DOWN |
| RP11-1000B6.3 | -2.74032 | -0.77185 | -12.7914 | 8.30E-34 | 5.32E-33 | 65.08084 | DOWN |
| RP1-278O22.2 | -2.2104 | -8.92013 | -12.7877 | 8.63E-34 | 5.52E-33 | 65.04223 | DOWN |
| LINC01508 | -2.43632 | -8.572 | -12.7776 | 9.58E-34 | 6.12E-33 | 64.93796 | DOWN |
| RP11-435O5.2 | -2.99446 | -3.56636 | -12.7624 | 1.12E-33 | 7.16E-33 | 64.77962 | DOWN |
| RP13-20L14.1 | -3.49263 | -7.45485 | -12.7313 | 1.55E-33 | 9.86E-33 | 64.45817 | DOWN |
| RP11-789C1.1 | -3.72322 | -7.65174 | -12.7288 | 1.59E-33 | 1.01E-32 | 64.43283 | DOWN |
| AC004448.5 | -2.99818 | -7.40386 | -12.6656 | 3.07E-33 | 1.93E-32 | 63.78015 | DOWN |
| AC008592.4 | -3.14242 | -7.83957 | -12.6581 | 3.31E-33 | 2.08E-32 | 63.70316 | DOWN |
| RP11-338N10.2 | -3.55196 | -5.54534 | -12.639 | 4.04E-33 | 2.54E-32 | 63.50612 | DOWN |
| AP000253.1 | -2.08834 | -5.22198 | -12.6114 | 5.38E-33 | 3.37E-32 | 63.22188 | DOWN |
| AC005754.8 | -2.60233 | -4.08092 | -12.5857 | 7.01E-33 | 4.39E-32 | 62.95776 | DOWN |
| CTD-3193K9.11 | -3.6691 | -2.57981 | -12.5839 | 7.14E-33 | 4.47E-32 | 62.93997 | DOWN |
| RP11-434D9.1 | -3.34693 | -4.69129 | -12.5628 | 8.88E-33 | 5.54E-32 | 62.72311 | DOWN |
| RP4-535B20.4 | -3.20913 | -4.96221 | -12.5605 | 9.09E-33 | 5.67E-32 | 62.69916 | DOWN |
| RP11-693J15.6 | -2.94464 | -7.91238 | -12.5302 | 1.24E-32 | 7.74E-32 | 62.38883 | DOWN |
| CTC-523E23.1 | -2.01882 | -3.98483 | -12.5018 | 1.66E-32 | 1.03E-31 | 62.09884 | DOWN |
| RP11-114F10.2 | -2.15708 | -9.03328 | -12.4744 | 2.20E-32 | 1.37E-31 | 61.81841 | DOWN |
| RP11-989E6.3 | -2.43639 | -8.28055 | -12.4698 | 2.31E-32 | 1.43E-31 | 61.77108 | DOWN |
| RP11-70D24.4 | -3.23408 | -6.35286 | -12.4579 | 2.61E-32 | 1.62E-31 | 61.64962 | DOWN |
| RP11-20J15.3 | -2.95406 | -3.97445 | -12.4226 | 3.75E-32 | 2.32E-31 | 61.29015 | DOWN |
| RP11-350F16.1 | -3.03771 | -8.20551 | -12.4031 | 4.57E-32 | 2.83E-31 | 61.09212 | DOWN |
| RP11-263K4.3 | -2.10274 | -8.18541 | -12.3901 | 5.23E-32 | 3.22E-31 | 60.95957 | DOWN |
| AC016995.3 | -2.95111 | -1.73361 | -12.3865 | 5.42E-32 | 3.34E-31 | 60.92304 | DOWN |
| RP11-554F20.1 | -2.2656 | -8.06726 | -12.3521 | 7.70E-32 | 4.73E-31 | 60.57363 | DOWN |
| RP5-839B4.8 | -3.48361 | -6.49927 | -12.3505 | 7.83E-32 | 4.80E-31 | 60.55798 | DOWN |
| RP11-646E18.2 | -2.26247 | -8.94163 | -12.3291 | 9.74E-32 | 5.94E-31 | 60.34076 | DOWN |
| RP11-834C11.10 | -3.05005 | -5.67124 | -12.2623 | 1.92E-31 | 1.16E-30 | 59.66512 | DOWN |
| RP11-724N1.1 | -2.64906 | -7.95384 | -12.2597 | 1.97E-31 | 1.19E-30 | 59.63857 | DOWN |
| RP11-134K13.4 | -3.23882 | -4.02877 | -12.2234 | 2.85E-31 | 1.72E-30 | 59.27248 | DOWN |
| RP11-534L20.5 | -3.42339 | -4.25795 | -12.21 | 3.26E-31 | 1.96E-30 | 59.13781 | DOWN |
| RP11-107N15.1 | -2.66862 | -2.35806 | -12.1823 | 4.32E-31 | 2.59E-30 | 58.85867 | DOWN |
| CTC-501O10.1 | -3.26829 | -6.99629 | -12.1494 | 6.03E-31 | 3.60E-30 | 58.52771 | DOWN |
| PKIA-AS1 | -2.92827 | -6.24213 | -12.0963 | 1.03E-30 | 6.13E-30 | 57.99619 | DOWN |
| LINC01546 | -3.35703 | -5.47586 | -12.086 | 1.14E-30 | 6.79E-30 | 57.89248 | DOWN |
| RP6-99M1.3 | -3.46057 | -5.84245 | -12.0819 | 1.19E-30 | 7.07E-30 | 57.85189 | DOWN |
| LINC01136 | -2.55488 | -2.78669 | -12.0812 | 1.20E-30 | 7.11E-30 | 57.8445 | DOWN |
| RP1-15D23.2 | -2.10634 | -8.89536 | -12.0596 | 1.49E-30 | 8.80E-30 | 57.62914 | DOWN |
| LINC01115 | -2.30652 | -8.64494 | -12.0579 | 1.51E-30 | 8.95E-30 | 57.61205 | DOWN |
| RP11-177B4.2 | -2.10662 | -8.24975 | -12.0155 | 2.32E-30 | 1.37E-29 | 57.18897 | DOWN |
| RP11-327J17.9 | -2.80149 | -3.97334 | -11.9954 | 2.84E-30 | 1.67E-29 | 56.98847 | DOWN |
| RP11-958F21.1 | -3.07906 | -7.88243 | -11.9952 | 2.84E-30 | 1.67E-29 | 56.98697 | DOWN |
| RP4-534N18.2 | -2.59001 | -8.4371 | -11.9904 | 2.98E-30 | 1.75E-29 | 56.93895 | DOWN |
| MGC45922 | -2.4208 | -3.37766 | -11.9358 | 5.14E-30 | 3.01E-29 | 56.39697 | DOWN |
| RP11-255P5.3 | -2.58936 | -8.09525 | -11.9072 | 6.84E-30 | 3.99E-29 | 56.11269 | DOWN |
| RP11-227G15.9 | -3.14084 | -6.79051 | -11.9037 | 7.08E-30 | 4.13E-29 | 56.07846 | DOWN |
| RP11-429A20.3 | -2.21463 | -8.91049 | -11.8771 | 9.24E-30 | 5.36E-29 | 55.81469 | DOWN |
| LINC00954 | -2.04822 | -3.5677 | -11.8717 | 9.74E-30 | 5.65E-29 | 55.76192 | DOWN |
| AC011747.4 | -2.15619 | -8.32962 | -11.8662 | 1.03E-29 | 5.96E-29 | 55.70724 | DOWN |
| RP11-325L12.7 | -3.08232 | -4.87972 | -11.8405 | 1.33E-29 | 7.67E-29 | 55.45349 | DOWN |
| LINC00960 | -2.31977 | -1.13854 | -11.8307 | 1.46E-29 | 8.44E-29 | 55.35728 | DOWN |
| LINC00536 | -2.03981 | -8.2058 | -11.8272 | 1.52E-29 | 8.72E-29 | 55.32274 | DOWN |
| WI2-87327B8.2 | -3.28559 | -3.96211 | -11.8099 | 1.80E-29 | 1.03E-28 | 55.152 | DOWN |
| LINC01230 | -2.31661 | -7.30551 | -11.7927 | 2.13E-29 | 1.22E-28 | 54.98279 | DOWN |
| RP4-781K5.5 | -2.58501 | -7.83003 | -11.7868 | 2.26E-29 | 1.30E-28 | 54.92438 | DOWN |
| RP11-428L9.2 | -2.07269 | -9.00235 | -11.7764 | 2.51E-29 | 1.44E-28 | 54.82222 | DOWN |
| RP11-130L8.2 | -2.56884 | -1.9086 | -11.7261 | 4.12E-29 | 2.35E-28 | 54.32776 | DOWN |
| RP11-109A6.3 | -3.12963 | -7.02745 | -11.7225 | 4.27E-29 | 2.43E-28 | 54.29332 | DOWN |
| RP11-495K9.3 | -2.66287 | -7.09128 | -11.7091 | 4.87E-29 | 2.77E-28 | 54.16165 | DOWN |
| RP11-92K15.3 | -2.26968 | -2.14584 | -11.6962 | 5.53E-29 | 3.13E-28 | 54.03527 | DOWN |
| AL133245.2 | -2.4564 | -6.38098 | -11.6905 | 5.85E-29 | 3.31E-28 | 53.97925 | DOWN |
| TTTY2B | -2.28219 | -8.19793 | -11.6314 | 1.05E-28 | 5.88E-28 | 53.40255 | DOWN |
| RP11-439C15.4 | -3.5288 | -5.08213 | -11.6282 | 1.08E-28 | 6.07E-28 | 53.37095 | DOWN |
| FLJ44511 | -2.25288 | -3.72442 | -11.6246 | 1.12E-28 | 6.28E-28 | 53.33546 | DOWN |
| FLJ43879 | -2.01103 | -7.86464 | -11.5772 | 1.78E-28 | 9.93E-28 | 52.87447 | DOWN |
| RP11-618L22.1 | -2.94331 | -6.11427 | -11.574 | 1.84E-28 | 1.02E-27 | 52.84311 | DOWN |
| AF038458.5 | -3.0258 | -6.61727 | -11.5635 | 2.03E-28 | 1.13E-27 | 52.74138 | DOWN |
| AC159540.2 | -3.18879 | -4.57412 | -11.5402 | 2.56E-28 | 1.42E-27 | 52.5149 | DOWN |
| RP11-390F4.3 | -2.28142 | -3.00082 | -11.5265 | 2.92E-28 | 1.62E-27 | 52.38242 | DOWN |
| RP11-148L24.1 | -2.92785 | -7.38837 | -11.5178 | 3.18E-28 | 1.76E-27 | 52.29789 | DOWN |
| RP11-803P9.1 | -2.03961 | -2.61388 | -11.4963 | 3.92E-28 | 2.16E-27 | 52.08973 | DOWN |
| LINC01481 | -2.75754 | -4.2844 | -11.4924 | 4.07E-28 | 2.25E-27 | 52.05187 | DOWN |
| AC073283.4 | -2.76446 | -4.64692 | -11.4879 | 4.25E-28 | 2.34E-27 | 52.0087 | DOWN |
| LA16c-352F7.1 | -2.69834 | -6.96908 | -11.4709 | 5.02E-28 | 2.76E-27 | 51.84463 | DOWN |
| LINC00694 | -2.83103 | -4.85112 | -11.4527 | 5.99E-28 | 3.29E-27 | 51.66905 | DOWN |
| LINC00304 | -2.24425 | -4.14504 | -11.4452 | 6.44E-28 | 3.54E-27 | 51.59663 | DOWN |
| XXyac-YX155B6.7 | -3.2988 | -3.88213 | -11.4322 | 7.31E-28 | 4.00E-27 | 51.47109 | DOWN |
| AC002064.5 | -2.70163 | -7.63172 | -11.4224 | 8.03E-28 | 4.39E-27 | 51.37682 | DOWN |
| RP11-243M5.1 | -2.77818 | -8.12683 | -11.4217 | 8.09E-28 | 4.42E-27 | 51.37 | DOWN |
| LINC00479 | -2.88992 | -4.05432 | -11.4132 | 8.78E-28 | 4.80E-27 | 51.28826 | DOWN |
| MIR7-3HG | -2.85075 | -7.89425 | -11.4054 | 9.48E-28 | 5.17E-27 | 51.21242 | DOWN |
| FAM224B | -2.97821 | -7.7943 | -11.3193 | 2.18E-27 | 1.18E-26 | 50.38609 | DOWN |
| RP11-289H16.1 | -2.22143 | -8.3343 | -11.3138 | 2.30E-27 | 1.25E-26 | 50.33372 | DOWN |
| LINC00371 | -2.17353 | -8.12017 | -11.2789 | 3.21E-27 | 1.74E-26 | 49.99983 | DOWN |
| LINC01485 | -3.19799 | -3.88242 | -11.2571 | 3.96E-27 | 2.14E-26 | 49.79172 | DOWN |
| CTD-3220F14.1 | -2.13835 | -2.02912 | -11.2163 | 5.86E-27 | 3.15E-26 | 49.40326 | DOWN |
| RP11-138A9.1 | -2.13003 | -6.37804 | -11.1624 | 9.81E-27 | 5.24E-26 | 48.89142 | DOWN |
| NAMA | -2.66858 | -4.83327 | -11.1068 | 1.67E-26 | 8.85E-26 | 48.3655 | DOWN |
| RP11-26L20.4 | -3.04916 | -3.88765 | -11.1049 | 1.70E-26 | 9.00E-26 | 48.34715 | DOWN |
| KB-1592A4.15 | -2.04116 | -7.8341 | -11.1033 | 1.72E-26 | 9.13E-26 | 48.3326 | DOWN |
| RP4-789D17.5 | -2.31129 | -1.85281 | -11.0852 | 2.04E-26 | 1.08E-25 | 48.16209 | DOWN |
| RP5-857K21.4 | -2.32435 | -1.81936 | -11.0838 | 2.07E-26 | 1.10E-25 | 48.14808 | DOWN |
| AC104655.3 | -3.72015 | -3.12942 | -11.083 | 2.09E-26 | 1.10E-25 | 48.14125 | DOWN |
| RP11-154D17.1 | -2.57143 | -6.89216 | -11.0708 | 2.35E-26 | 1.23E-25 | 48.02555 | DOWN |
| RP11-445N18.5 | -2.95283 | -7.59712 | -11.0487 | 2.89E-26 | 1.52E-25 | 47.81761 | DOWN |
| XXbac-BPG13B8.10 | -2.47963 | -7.95144 | -11.042 | 3.08E-26 | 1.62E-25 | 47.75507 | DOWN |
| RP5-837J1.4 | -2.98179 | -6.09365 | -11.0415 | 3.10E-26 | 1.62E-25 | 47.75022 | DOWN |
| GS1-24F4.2 | -2.63399 | -7.17202 | -11.0215 | 3.74E-26 | 1.96E-25 | 47.56196 | DOWN |
| RP11-672A2.6 | -2.58764 | -7.47079 | -11.0069 | 4.30E-26 | 2.25E-25 | 47.4249 | DOWN |
| RP11-466L17.1 | -2.13089 | -5.65648 | -10.9923 | 4.93E-26 | 2.58E-25 | 47.28736 | DOWN |
| LINC00574 | -2.12857 | -6.98747 | -10.981 | 5.49E-26 | 2.86E-25 | 47.18163 | DOWN |
| RP11-504P24.9 | -2.57534 | -2.69665 | -10.9484 | 7.46E-26 | 3.88E-25 | 46.87668 | DOWN |
| RP11-419I17.1 | -2.06078 | -2.40542 | -10.9357 | 8.41E-26 | 4.37E-25 | 46.75755 | DOWN |
| RP11-305L7.1 | -2.13509 | -2.431 | -10.9205 | 9.70E-26 | 5.02E-25 | 46.61587 | DOWN |
| RP11-473E2.4 | -2.89765 | -7.72951 | -10.8949 | 1.23E-25 | 6.37E-25 | 46.37718 | DOWN |
| LINC00106 | -2.79867 | -1.42862 | -10.8913 | 1.28E-25 | 6.58E-25 | 46.34362 | DOWN |
| RP4-758J18.10 | -2.23938 | -0.6826 | -10.8911 | 1.28E-25 | 6.59E-25 | 46.3421 | DOWN |
| RP11-255P5.2 | -2.31845 | -7.8522 | -10.8714 | 1.54E-25 | 7.90E-25 | 46.15849 | DOWN |
| CTD-2349P21.5 | -2.8134 | -6.00894 | -10.8657 | 1.62E-25 | 8.33E-25 | 46.1056 | DOWN |
| TTTY10 | -2.75319 | -6.86177 | -10.8499 | 1.88E-25 | 9.65E-25 | 45.95796 | DOWN |
| RP11-219E7.4 | -2.5982 | -8.35532 | -10.8407 | 2.05E-25 | 1.05E-24 | 45.87259 | DOWN |
| RP11-360I2.1 | -3.56217 | -6.12861 | -10.8268 | 2.34E-25 | 1.19E-24 | 45.74338 | DOWN |
| CTD-2339F6.1 | -2.33945 | -7.18553 | -10.8249 | 2.38E-25 | 1.21E-24 | 45.72652 | DOWN |
| RP11-406A9.2 | -2.99986 | -7.73528 | -10.8217 | 2.45E-25 | 1.25E-24 | 45.69659 | DOWN |
| FRMD6-AS2 | -2.06445 | -8.80685 | -10.8142 | 2.63E-25 | 1.34E-24 | 45.62684 | DOWN |
| RP11-863P13.6 | -2.93046 | -8.48252 | -10.807 | 2.81E-25 | 1.43E-24 | 45.55988 | DOWN |
| RP11-963H4.3 | -3.12381 | -5.35306 | -10.7985 | 3.04E-25 | 1.55E-24 | 45.48171 | DOWN |
| RP11-89H19.2 | -2.29372 | -4.45837 | -10.7866 | 3.40E-25 | 1.73E-24 | 45.3712 | DOWN |
| CTD-2008L17.2 | -2.705 | -6.39631 | -10.7855 | 3.43E-25 | 1.74E-24 | 45.36134 | DOWN |
| RP11-324D17.2 | -2.13165 | -8.86536 | -10.7542 | 4.60E-25 | 2.33E-24 | 45.07137 | DOWN |
| RP11-287D1.4 | -2.60785 | -4.12298 | -10.7248 | 6.04E-25 | 3.06E-24 | 44.80054 | DOWN |
| RP11-662M24.2 | -2.45434 | -8.97588 | -10.724 | 6.09E-25 | 3.08E-24 | 44.79284 | DOWN |
| AC009120.5 | -2.43207 | -2.38887 | -10.6264 | 1.50E-24 | 7.52E-24 | 43.8968 | DOWN |
| LINC00621 | -2.04443 | -8.63222 | -10.6251 | 1.52E-24 | 7.61E-24 | 43.88494 | DOWN |
| CTB-129P6.11 | -2.83728 | -7.78544 | -10.6173 | 1.63E-24 | 8.15E-24 | 43.81359 | DOWN |
| RP11-13J8.1 | -2.13682 | -8.05946 | -10.6109 | 1.73E-24 | 8.64E-24 | 43.75447 | DOWN |
| CTD-2235C13.3 | -2.51162 | -2.46718 | -10.5792 | 2.32E-24 | 1.15E-23 | 43.46542 | DOWN |
| RP11-800A3.3 | -2.5033 | -7.84206 | -10.5647 | 2.65E-24 | 1.31E-23 | 43.33297 | DOWN |
| LINC00705 | -2.07461 | -8.06237 | -10.525 | 3.82E-24 | 1.89E-23 | 42.97128 | DOWN |
| RP11-154H23.3 | -2.02547 | -5.66352 | -10.5175 | 4.09E-24 | 2.02E-23 | 42.90342 | DOWN |
| RP11-1136G11.8 | -2.67124 | -7.70694 | -10.5123 | 4.29E-24 | 2.12E-23 | 42.85633 | DOWN |
| LINC00264 | -2.49706 | -6.80733 | -10.4715 | 6.22E-24 | 3.05E-23 | 42.48656 | DOWN |
| RP11-2B6.2 | -2.8583 | -2.56924 | -10.4628 | 6.74E-24 | 3.30E-23 | 42.4072 | DOWN |
| RP11-435O5.7 | -2.84219 | -5.65124 | -10.4407 | 8.24E-24 | 4.03E-23 | 42.2074 | DOWN |
| MIR4500HG | -2.73732 | -6.38285 | -10.4141 | 1.05E-23 | 5.12E-23 | 41.96678 | DOWN |
| RP11-672A2.5 | -2.6277 | -7.49249 | -10.4129 | 1.06E-23 | 5.17E-23 | 41.95596 | DOWN |
| AC108004.2 | -2.24977 | -7.57129 | -10.3787 | 1.45E-23 | 7.01E-23 | 41.6481 | DOWN |
| CPEB2-AS1 | -2.0706 | -6.34341 | -10.3555 | 1.79E-23 | 8.62E-23 | 41.43936 | DOWN |
| LINC00393 | -3.19622 | -6.70514 | -10.3519 | 1.85E-23 | 8.89E-23 | 41.40772 | DOWN |
| RP11-270M14.5 | -2.49742 | -7.81228 | -10.3217 | 2.43E-23 | 1.16E-22 | 41.13625 | DOWN |
| CTD-2554C21.2 | -2.81157 | -3.86884 | -10.3163 | 2.55E-23 | 1.22E-22 | 41.08797 | DOWN |
| GS1-304P7.3 | -2.72173 | -7.13833 | -10.2832 | 3.44E-23 | 1.64E-22 | 40.79141 | DOWN |
| RP11-322E11.5 | -2.1319 | -1.78835 | -10.2676 | 3.95E-23 | 1.88E-22 | 40.65268 | DOWN |
| EGOT | -2.33147 | -6.67346 | -10.2562 | 4.38E-23 | 2.09E-22 | 40.55098 | DOWN |
| RP11-28H5.2 | -2.69146 | -3.76039 | -10.2514 | 4.57E-23 | 2.18E-22 | 40.50785 | DOWN |
| RP11-444D3.1 | -2.39206 | -1.88906 | -10.1744 | 9.13E-23 | 4.30E-22 | 39.8229 | DOWN |
| RP11-12A2.3 | -2.17717 | -6.81156 | -10.1459 | 1.18E-22 | 5.54E-22 | 39.5702 | DOWN |
| RP11-56F10.3 | -2.53647 | -7.69864 | -10.1423 | 1.22E-22 | 5.71E-22 | 39.53889 | DOWN |
| CTD-3179P9.1 | -2.55442 | -7.67109 | -10.1319 | 1.33E-22 | 6.25E-22 | 39.44706 | DOWN |
| RP11-171I2.3 | -2.98703 | -5.58275 | -10.1244 | 1.43E-22 | 6.68E-22 | 39.38045 | DOWN |
| RP11-150O12.3 | -2.10478 | -4.66141 | -10.052 | 2.71E-22 | 1.27E-21 | 38.74274 | DOWN |
| CHODL-AS1 | -2.32385 | -7.59124 | -10.045 | 2.89E-22 | 1.34E-21 | 38.68054 | DOWN |
| LINC01405 | -2.79181 | -7.74373 | -9.98773 | 4.79E-22 | 2.22E-21 | 38.17878 | DOWN |
| RP11-180P8.3 | -2.47811 | -7.0923 | -9.98562 | 4.88E-22 | 2.26E-21 | 38.16038 | DOWN |
| RP11-259K5.1 | -2.20685 | -7.20023 | -9.93409 | 7.69E-22 | 3.55E-21 | 37.71052 | DOWN |
| CMP21-97G8.1 | -2.88364 | -7.04863 | -9.92709 | 8.17E-22 | 3.77E-21 | 37.64959 | DOWN |
| RP4-712E4.1 | -2.27904 | -8.15291 | -9.90838 | 9.63E-22 | 4.43E-21 | 37.48679 | DOWN |
| RP11-314C9.2 | -2.30888 | -8.74368 | -9.90801 | 9.66E-22 | 4.44E-21 | 37.48361 | DOWN |
| LINC01336 | -2.83956 | -5.96597 | -9.89685 | 1.07E-21 | 4.90E-21 | 37.38659 | DOWN |
| RP11-20G13.1 | -3.35266 | -4.28503 | -9.87268 | 1.32E-21 | 6.03E-21 | 37.1768 | DOWN |
| LINC01118 | -2.3078 | -5.47641 | -9.87216 | 1.32E-21 | 6.05E-21 | 37.17233 | DOWN |
| RP11-15E1.5 | -2.10728 | -5.0554 | -9.85156 | 1.58E-21 | 7.24E-21 | 36.99387 | DOWN |
| XXyac-YR29IB3.1 | -2.34586 | -8.63133 | -9.80206 | 2.44E-21 | 1.11E-20 | 36.56609 | DOWN |
| CTD-3010D24.3 | -2.03745 | -8.2405 | -9.78705 | 2.78E-21 | 1.26E-20 | 36.43678 | DOWN |
| RP5-999L4.2 | -3.03273 | -5.53217 | -9.78348 | 2.87E-21 | 1.30E-20 | 36.40597 | DOWN |
| LINC00605 | -2.19277 | -6.49144 | -9.75378 | 3.71E-21 | 1.67E-20 | 36.15053 | DOWN |
| GATA6-AS1 | -2.09035 | -3.27826 | -9.68914 | 6.49E-21 | 2.91E-20 | 35.59652 | DOWN |
| RP11-834C11.6 | -2.75712 | -5.96118 | -9.63814 | 1.01E-20 | 4.49E-20 | 35.16143 | DOWN |
| RP11-153K11.3 | -2.24156 | -8.37275 | -9.63623 | 1.02E-20 | 4.56E-20 | 35.14523 | DOWN |
| LINC00868 | -2.37083 | -8.36648 | -9.61637 | 1.21E-20 | 5.40E-20 | 34.97623 | DOWN |
| PGM5-AS1 | -2.56667 | -7.24871 | -9.54582 | 2.22E-20 | 9.79E-20 | 34.37831 | DOWN |
| RP11-571M6.18 | -2.32793 | -2.65526 | -9.54506 | 2.23E-20 | 9.84E-20 | 34.37189 | DOWN |
| RP11-148B18.3 | -2.69961 | -6.77242 | -9.47566 | 4.03E-20 | 1.77E-19 | 33.78705 | DOWN |
| AC005606.15 | -3.20048 | -4.03026 | -9.44152 | 5.39E-20 | 2.35E-19 | 33.50052 | DOWN |
| RP11-389O22.1 | -2.93126 | -5.24765 | -9.43324 | 5.78E-20 | 2.52E-19 | 33.43116 | DOWN |
| RP11-973F15.2 | -2.1834 | -8.37727 | -9.41583 | 6.69E-20 | 2.92E-19 | 33.28553 | DOWN |
| RP13-1039J1.2 | -2.39901 | -7.24466 | -9.39576 | 7.93E-20 | 3.45E-19 | 33.11782 | DOWN |
| RP11-1152H14.1 | -2.23845 | -3.61249 | -9.3948 | 7.99E-20 | 3.47E-19 | 33.10976 | DOWN |
| RP11-575H3.1 | -2.91608 | -4.86082 | -9.37611 | 9.36E-20 | 4.06E-19 | 32.95387 | DOWN |
| MEOX2-AS1 | -2.81892 | -4.65927 | -9.35044 | 1.16E-19 | 5.00E-19 | 32.74016 | DOWN |
| RP5-851M4.1 | -2.61129 | -7.26976 | -9.34066 | 1.26E-19 | 5.43E-19 | 32.65882 | DOWN |
| MIMT1 | -2.02292 | -8.01928 | -9.31548 | 1.56E-19 | 6.69E-19 | 32.44979 | DOWN |
| CH507-42P11.5 | -2.59126 | -6.58748 | -9.30976 | 1.63E-19 | 7.01E-19 | 32.40241 | DOWN |
| RP11-180P8.1 | -2.39178 | -7.63518 | -9.29394 | 1.86E-19 | 7.99E-19 | 32.27128 | DOWN |
| AP000350.5 | -2.28191 | -4.01892 | -9.28528 | 2.00E-19 | 8.58E-19 | 32.19964 | DOWN |
| RP11-89N17.1 | -2.16138 | -6.28677 | -9.26977 | 2.28E-19 | 9.75E-19 | 32.07141 | DOWN |
| RP11-120K24.3 | -2.36319 | -3.94938 | -9.24568 | 2.79E-19 | 1.19E-18 | 31.8726 | DOWN |
| AC073257.2 | -2.59496 | -6.13525 | -9.22345 | 3.36E-19 | 1.43E-18 | 31.68953 | DOWN |
| RP11-121E16.1 | -2.15306 | -7.38633 | -9.1782 | 4.89E-19 | 2.07E-18 | 31.31787 | DOWN |
| LINC00202-2 | -2.05924 | -8.4803 | -9.10063 | 9.27E-19 | 3.88E-18 | 30.68411 | DOWN |
| BACH1-AS1 | -2.14216 | -7.69741 | -9.06259 | 1.27E-18 | 5.28E-18 | 30.37487 | DOWN |
| LINC00266-1 | -2.69218 | -5.27576 | -9.04886 | 1.42E-18 | 5.89E-18 | 30.26343 | DOWN |
| RP11-108M9.3 | -2.13126 | -5.16351 | -9.03767 | 1.55E-18 | 6.45E-18 | 30.17278 | DOWN |
| RP11-95P13.1 | -2.8983 | -5.26605 | -9.00106 | 2.10E-18 | 8.65E-18 | 29.87678 | DOWN |
| AC073133.2 | -2.05846 | -8.50392 | -8.98756 | 2.34E-18 | 9.65E-18 | 29.76784 | DOWN |
| RP11-38L15.8 | -2.10964 | -2.87472 | -8.97404 | 2.61E-18 | 1.08E-17 | 29.65896 | DOWN |
| KC6 | -2.91785 | -5.23633 | -8.97272 | 2.64E-18 | 1.09E-17 | 29.64833 | DOWN |
| RP11-696F12.1 | -2.12681 | -8.40858 | -8.96468 | 2.82E-18 | 1.16E-17 | 29.58358 | DOWN |
| RP11-440I14.3 | -2.54595 | -4.93468 | -8.96338 | 2.85E-18 | 1.17E-17 | 29.57312 | DOWN |
| AC007879.2 | -2.3415 | -4.65207 | -8.90854 | 4.45E-18 | 1.82E-17 | 29.13299 | DOWN |
| RP11-326C3.15 | -2.50248 | -4.31806 | -8.87621 | 5.78E-18 | 2.35E-17 | 28.87446 | DOWN |
| RP11-424G14.1 | -3.09317 | -4.1236 | -8.87147 | 6.00E-18 | 2.44E-17 | 28.83663 | DOWN |
| RP5-1116H23.4 | -2.98204 | -5.38775 | -8.80941 | 9.89E-18 | 3.99E-17 | 28.34282 | DOWN |
| RP11-247A12.7 | -2.40716 | -4.08946 | -8.6757 | 2.88E-17 | 1.14E-16 | 27.28817 | DOWN |
| RP11-686O6.1 | -2.55975 | -5.29655 | -8.65884 | 3.29E-17 | 1.30E-16 | 27.15614 | DOWN |
| RP11-254I22.1 | -2.09422 | -7.37673 | -8.63823 | 3.87E-17 | 1.53E-16 | 26.99499 | DOWN |
| RP11-100L22.1 | -2.15273 | -7.74831 | -8.59138 | 5.60E-17 | 2.19E-16 | 26.6298 | DOWN |
| LINC00545 | -2.73255 | -5.62116 | -8.56551 | 6.86E-17 | 2.68E-16 | 26.42886 | DOWN |
| LINC00683 | -2.00282 | -6.04325 | -8.54915 | 7.80E-17 | 3.05E-16 | 26.30205 | DOWN |
| AC019172.2 | -2.47304 | -5.93371 | -8.48551 | 1.28E-16 | 4.98E-16 | 25.81061 | DOWN |
| RP11-454C18.1 | -2.38046 | -7.54919 | -8.43928 | 1.84E-16 | 7.09E-16 | 25.45539 | DOWN |
| LINC01549 | -2.47145 | -6.18887 | -8.42297 | 2.09E-16 | 8.03E-16 | 25.3305 | DOWN |
| RP11-136C24.2 | -2.03839 | -8.12558 | -8.41592 | 2.20E-16 | 8.47E-16 | 25.27657 | DOWN |
| RP11-340I6.8 | -2.10676 | -7.41677 | -8.36885 | 3.17E-16 | 1.21E-15 | 24.9174 | DOWN |
| RP11-200A1.1 | -2.45663 | -7.71505 | -8.36352 | 3.30E-16 | 1.26E-15 | 24.87681 | DOWN |
| LINC00887 | -2.1173 | -4.04344 | -8.3505 | 3.65E-16 | 1.39E-15 | 24.77781 | DOWN |
| RP11-70D24.3 | -2.27096 | -6.00622 | -8.34623 | 3.77E-16 | 1.44E-15 | 24.74535 | DOWN |
| RP11-15I11.3 | -2.42447 | -1.44946 | -8.30584 | 5.15E-16 | 1.96E-15 | 24.43917 | DOWN |
| RP5-1116H23.5 | -2.40372 | -6.34069 | -8.26225 | 7.18E-16 | 2.72E-15 | 24.11012 | DOWN |
| CTC-344H19.4 | -2.33817 | -5.09204 | -8.2443 | 8.24E-16 | 3.11E-15 | 23.97497 | DOWN |
| RP11-573G6.6 | -2.07209 | -4.48639 | -8.23736 | 8.68E-16 | 3.27E-15 | 23.92279 | DOWN |
| RP5-857K21.1 | -2.19162 | -6.68492 | -8.18864 | 1.26E-15 | 4.71E-15 | 23.55758 | DOWN |
| RP13-455A7.1 | -2.1489 | -8.61188 | -8.1766 | 1.38E-15 | 5.15E-15 | 23.46758 | DOWN |
| RP11-77H9.8 | -2.39828 | -5.41462 | -8.14996 | 1.68E-15 | 6.28E-15 | 23.26891 | DOWN |
| RP5-1007H16.1 | -2.21396 | -5.41527 | -8.14732 | 1.72E-15 | 6.40E-15 | 23.24924 | DOWN |
| RP13-726E6.2 | -2.06756 | -5.5854 | -8.09752 | 2.50E-15 | 9.21E-15 | 22.87936 | DOWN |
| TPRG1-AS1 | -2.02358 | -3.74395 | -8.09741 | 2.50E-15 | 9.22E-15 | 22.87852 | DOWN |
| AP001048.4 | -2.17557 | -8.23481 | -8.07799 | 2.90E-15 | 1.06E-14 | 22.73477 | DOWN |
| CTB-32O4.3 | -2.02265 | -5.76334 | -8.04672 | 3.66E-15 | 1.34E-14 | 22.50388 | DOWN |
| RP11-77E14.2 | -2.2416 | -5.84846 | -8.0462 | 3.67E-15 | 1.34E-14 | 22.50006 | DOWN |
| RP11-1100L3.8 | -2.46027 | 0.212705 | -8.00862 | 4.86E-15 | 1.77E-14 | 22.22364 | DOWN |
| RP11-258F22.1 | -2.14019 | -5.72396 | -7.97201 | 6.38E-15 | 2.31E-14 | 21.95534 | DOWN |
| RP11-552D4.1 | -2.18241 | -7.05908 | -7.91648 | 9.62E-15 | 3.45E-14 | 21.55044 | DOWN |
| RP11-440G5.2 | -2.29183 | -4.8706 | -7.88724 | 1.19E-14 | 4.27E-14 | 21.33814 | DOWN |
| RP11-338N10.3 | -2.78032 | -6.26498 | -7.84569 | 1.62E-14 | 5.76E-14 | 21.03765 | DOWN |
| LINC01152 | -2.03928 | -0.63903 | -7.82382 | 1.90E-14 | 6.74E-14 | 20.87998 | DOWN |
| RP11-134O21.1 | -2.22242 | -6.37785 | -7.80102 | 2.24E-14 | 7.95E-14 | 20.716 | DOWN |
| AP001059.7 | -2.37698 | -5.80501 | -7.77852 | 2.64E-14 | 9.33E-14 | 20.55457 | DOWN |
| XIST | -3.43895 | -0.5515 | -7.76883 | 2.84E-14 | 9.98E-14 | 20.48523 | DOWN |
| TTTY14 | -3.30793 | -2.97146 | -7.69371 | 4.88E-14 | 1.70E-13 | 19.9497 | DOWN |
| RP11-112J3.16 | -2.26486 | -3.77238 | -7.61291 | 8.72E-14 | 3.02E-13 | 19.37866 | DOWN |
| RP11-414C23.1 | -2.64718 | -5.62113 | -7.58123 | 1.09E-13 | 3.77E-13 | 19.15619 | DOWN |
| SCARNA2 | -2.46903 | -8.13359 | -7.57217 | 1.17E-13 | 4.01E-13 | 19.0927 | DOWN |
| RP13-463N16.6 | -2.25094 | -5.4633 | -7.56036 | 1.27E-13 | 4.35E-13 | 19.00998 | DOWN |
| XX-C2158C6.1 | -2.40441 | -6.47287 | -7.48974 | 2.09E-13 | 7.11E-13 | 18.51798 | DOWN |
| RP11-11C20.3 | -2.07669 | -6.03762 | -7.48963 | 2.09E-13 | 7.11E-13 | 18.51723 | DOWN |
| U47924.32 | -2.04148 | -3.94787 | -7.4511 | 2.74E-13 | 9.27E-13 | 18.25043 | DOWN |
| RP11-415J8.7 | -2.00084 | -4.15687 | -7.44197 | 2.93E-13 | 9.87E-13 | 18.18739 | DOWN |
| XX-C2158C6.3 | -2.1968 | -7.01607 | -7.39131 | 4.17E-13 | 1.40E-12 | 17.83876 | DOWN |
| RP5-1142J19.1 | -2.01219 | -4.01048 | -7.34309 | 5.83E-13 | 1.95E-12 | 17.50884 | DOWN |
| RP11-284F21.11 | -2.10679 | -6.76864 | -7.33605 | 6.13E-13 | 2.04E-12 | 17.4608 | DOWN |
| CTC-559E9.4 | -2.16383 | -5.04281 | -7.31329 | 7.17E-13 | 2.38E-12 | 17.30587 | DOWN |
| RP11-527N22.2 | -2.14036 | -5.24389 | -7.2909 | 8.37E-13 | 2.77E-12 | 17.15383 | DOWN |
| CH17-373J23.1 | -2.56692 | -4.51776 | -7.27871 | 9.10E-13 | 3.01E-12 | 17.07125 | DOWN |
| RP11-430G17.3 | -2.18575 | -4.39612 | -7.13199 | 2.48E-12 | 8.06E-12 | 16.08626 | DOWN |
| RP11-370I10.11 | -2.09652 | -5.63668 | -7.10554 | 2.97E-12 | 9.61E-12 | 15.91061 | DOWN |
| RNU11 | -2.78541 | -7.56912 | -6.8159 | 2.03E-11 | 6.34E-11 | 14.02357 | DOWN |
| RP3-324O17.8 | -2.16408 | -6.21046 | -6.61752 | 7.28E-11 | 2.22E-10 | 12.77081 | DOWN |
| AP006621.6 | -2.13355 | -6.23186 | -6.12632 | 1.50E-09 | 4.33E-09 | 9.810246 | DOWN |
