# Supplementary material for: Tumor-infiltrating macrophage associated lncRNA signature in cutaneous melanoma: implications for diagnosis, prognosis, and immunotherapy
Source: Aging (Albany NY). 2024 Mar 13;16(5):4518–40. doi: 10.18632/aging.205606 (PMC10968696; doi:10.18632/aging.205606)
Supplement: Supplementary Table 3 [file aging-16-205606-s004.pdf]

SUPPLEMENTARY TABLE

Supplementary Table 3. Univariate regression analysis for the six selected DEMlncRNAs.

lncRNA_id	lncRNA_symbol	HR	z-value	*p*-value	Lower	Upper
ENSG00000152931	PART1	1.075214	2.727958	0.006373	1.020626	1.132721
ENSG00000228784	LINC00954	0.924471	−2.23804	0.025218	0.863028	0.99029
ENSG00000246430	LINC00968	0.931976	−2.38642	0.017013	0.879584	0.98749
ENSG00000183674	LINC00518	1.098574	2.486129	0.012914	1.020096	1.18309
ENSG00000256128	LINC00944	0.940095	−2.88669	0.003893	0.90148	0.980364
ENSG00000176659	C20orf197	0.920155	−2.21562	0.026718	0.854853	0.990444
