# Supplementary material for: Tumor-infiltrating macrophage associated lncRNA signature in cutaneous melanoma: implications for diagnosis, prognosis, and immunotherapy
Source: Aging (Albany NY). 2024 Mar 13;16(5):4518–40. doi: 10.18632/aging.205606 (PMC10968696; doi:10.18632/aging.205606)
Supplement: Supplementary Table 4 [file aging-16-205606-s005.docx]

Supplementary Table 4. The pathway analysis of the six selected DEMlncRNAs.

| **Category** | **Term** | **Description** | **LogP** | **Log(q-value)** | **InTerm_InList** | **Symbols** |  |
| --- | --- | --- | --- | --- | --- | --- | --- |
| KEGG Pathway | hsa03040 | Spliceosome | -21.5268 | -18.438 | 15/134 | HNRNPA1,HNRNPC,HNRNPK,HNRNPU,HNRNPM,SRSF1,SRSF3,SRSF7,U2AF1,SRSF9,EIF4A3,PRPF8,SRSF10,SF3A3,U2AF2 | |
| KEGG Pathway | hsa05168 | Herpes simplex infection | -4.29211 | -2.014 | 5/185 | HNRNPK,SRSF1,SRSF3,SRSF7,SRSF9 | |
| KEGG Pathway | hsa05202 | Transcriptional misregulation in cancer | -2.14976 | -0.024 | 3/180 | EWSR1,FUS,TAF15 | |
| KEGG Pathway | hsa03008 | Ribosome biogenesis in eukaryotes | -4.09135 | -1.825 | 4/105 | DKC1,FBL,NOP56,NOP58 | |
| KEGG Pathway | hsa03013 | RNA transport | -3.2803 | -1.072 | 4/171 | UPF1,FXR1,FXR2,EIF4A3 | |
| KEGG Pathway | hsa03015 | mRNA surveillance pathway | -4.33409 | -2.048 | 4/91 | UPF1,EIF4A3,CSTF2T,MSI2 | |
| KEGG Pathway | hsa03015 | mRNA surveillance pathway | -4.33409 | -2.048 | 4/91 | UPF1,EIF4A3,CSTF2T,MSI2 | |
| GO Biological Processes | GO:0006397 | mRNA processing | -43.7144 | -39.395 | 34/508 | ADAR,ELAVL1,FUS,GTF2F1,HNRNPA1,HNRNPC,HNRNPK,HNRNPL,HNRNPU,HNRNPM,PCBP2,PTBP1,SRSF1,SRSF3,SRSF7,TIA1,U2AF1,FXR1,SRSF9,FXR2,EIF4A3,MBNL2,RBM5,PRPF8,SRSF10,SF3A3,U2AF2,CSTF2T,TARDBP,RBFOX2,SLTM,BUD13,YTHDC1,LSM11,TAF15,DKC1,FBL,UPF1,NOP56,IGF2BP1,IGF2BP3,IGF2BP2,NOP58 | |
| GO Biological Processes | GO:1903311 | regulation of mRNA metabolic process | -42.0385 | -38.183 | 30/328 | ELAVL1,FUS,HNRNPA1,HNRNPC,HNRNPK,HNRNPL,HNRNPU,MOV10,HNRNPM,PTBP1,UPF1,SRSF1,SRSF3,SRSF7,TIA1,FXR1,TAF15,SRSF9,FXR2,MBNL2,RBM5,IGF2BP1,IGF2BP3,IGF2BP2,SRSF10,U2AF2,TARDBP,RBFOX2,SLTM,YTHDC1 | |
| GO Biological Processes | GO:0006403 | RNA localization | -21.1141 | -18.050 | 17/236 | DKC1,FBL,HNRNPA1,HNRNPU,UPF1,SRSF1,SRSF3,SRSF7,U2AF1,SRSF9,EIF4A3,IGF2BP1,IGF2BP3,IGF2BP2,U2AF2,NOP58,YTHDC1,MOV10,PRPF8,SF3A3,BUD13,CSTF2T,LSM11,ELAVL1,TARDBP,SRSF10,HNRNPK | |
| GO Biological Processes | GO:0043487 | regulation of RNA stability | -19.2867 | -16.246 | 15/187 | DKC1,ELAVL1,FUS,HNRNPC,HNRNPU,MOV10,HNRNPM,UPF1,FXR1,TAF15,FXR2,IGF2BP1,IGF2BP3,IGF2BP2,TARDBP,EIF4A3,HNRNPL,PTBP1,TIA1,HNRNPA1 | |
| GO Biological Processes | GO:0022613 | ribonucleoprotein complex biogenesis | -10.8353 | -8.272 | 13/462 | ADAR,DKC1,FBL,SRSF1,SRSF9,EIF4A3,RBM5,NOP56,PRPF8,SRSF10,SF3A3,NOP58,YTHDC1 | |
| GO Biological Processes | GO:0032204 | regulation of telomere maintenance | -6.04242 | -3.648 | 5/81 | DKC1,HNRNPA1,HNRNPC,HNRNPU,UPF1,YTHDC1,FUS | |
| GO Biological Processes | GO:0061158 | 3'-UTR-mediated mRNA destabilization | -5.18936 | -2.824 | 3/17 | MOV10,UPF1,TARDBP | |
| GO Biological Processes | GO:0045727 | positive regulation of translation | -4.99697 | -2.644 | 5/132 | ELAVL1,HNRNPL,FXR1,FXR2,EIF4A3,UPF1,FUS,HNRNPK | |
| GO Biological Processes | GO:0040029 | regulation of gene expression, epigenetic | -4.30032 | -2.019 | 8/613 | ADAR,ELAVL1,HNRNPU,MOV10,UPF1,FXR1,DGCR8,YTHDC1 | |
| GO Biological Processes | GO:0009451 | RNA modification | -3.39952 | -1.184 | 4/159 | ADAR,DKC1,FBL,RBM47,HNRNPC,HNRNPU | |
| GO Biological Processes | GO:0019080 | viral gene expression | -3.07523 | -0.873 | 4/194 | GTF2F1,PCBP2,PTBP1,TARDBP,ADAR,MOV10 | |
|  |  |  |  |  |  |  |  |
|  |  |  |  |  |  |  |  |
|  |  |  |  |  |  |  |  |
| cancer_type | lncRNA_id | lncRNA_symbol | immune_pathway | p_value | p_adjust | ES | score |
| SKCM | ENSG00000152931 | PART1 | Cytokine Receptors | 0.002051 | 0.034872 | 0.441087 | 0.995897 |
| SKCM | ENSG00000176659 | C20orf197 | Cytokines | 0.002018 | 0.034309 | 0.375738 | 0.995964 |
| SKCM | ENSG00000183674 | LINC00518 | Antigen Processing and Presentation | 0.0016 | 0.018438 | 0.544095 | 0.9968 |
| SKCM | ENSG00000183674 | LINC00518 | TGFb Family Member | 0.002169 | 0.018438 | -0.74057 | -0.99566 |
| SKCM | ENSG00000246430 | LINC00968 | Cytokine Receptors | 0.001135 | 0.018378 | 0.411311 | 0.99773 |
| SKCM | ENSG00000256128 | LINC00944 | Antigen Processing and Presentation | 0.001172 | 0.003279 | 0.541138 | 0.997655 |
| SKCM | ENSG00000256128 | LINC00944 | Antimicrobials | 0.001022 | 0.003279 | 0.51748 | 0.997955 |
| SKCM | ENSG00000256128 | LINC00944 | Chemokine Receptors | 0.001357 | 0.003279 | 0.639346 | 0.997286 |
| SKCM | ENSG00000256128 | LINC00944 | Interleukins Receptor | 0.001381 | 0.003279 | 0.71051 | 0.997238 |
| SKCM | ENSG00000256128 | LINC00944 | Natural Killer Cell Cytotoxicity | 0.001179 | 0.003279 | 0.529671 | 0.997642 |
| SKCM | ENSG00000256128 | LINC00944 | TNF Family Members | 0.001543 | 0.003279 | 0.803357 | 0.996914 |
